# HCV incidence and prevalence trends among people who use drugs in France 2004 to 2023

**DOI:** 10.1038/s41467-026-78598-3

**Published:** 2026-10-11

**Authors:** Chiara E. Sabbatini, Yann Le Strat, Christophe Bonaldi, Cécile Brouard, Martin Audran, Leïla Saboni, Jean-Baptiste Richard, Amélie David, Catherine Gaudy-Graffin, Stéphane Chevaliez, Francis Barin, Karl Stefic, Marie Jauffret-Roustide

**Affiliations:** 1 Santé publique France, French National Public Health Agency, Saint-Maurice, France; 2 Centre d’étude des Mouvements Sociaux (Inserm U1276/CNRS UMR 8044/EHESS), Paris, France; 3 Université de Tours, Inserm U1259 MAVIVH, and CHU de Tours, Laboratoire associé au CNR du VIH, Tours, France; 4 Department of Virology, French National Reference Centre for hepatitis B, C and delta viruses, Hôpital Henri Mondor (AP-HP), Université Paris-Est, Créteil, France; 5 Centre Hospitalier du Vinatier, Lyon, France; 6 Institut Universitaire sur les dépendances, Montréal, Québec Canada

**Keywords:** Hepatitis C, Statistics, Epidemiology

## Abstract

Hepatitis C virus (HCV) remains a key public health challenge, particularly among people who use and inject drugs (PWUD, PWID). While direct-acting antivirals (DAA) have revolutionized HCV treatment, meeting the WHO’s 2030 elimination target requires accurate estimation of incidence and prevalence to guide prevention and care. We analyze cross-sectional surveys conducted in France in 2004, 2013, and 2023, recruiting PWUD at harm reduction services and collecting behavioral and biological data. We estimate age-and time-dependent prevalence of HCV antibodies and HCV RNA using multivariate regression models while incidence was estimated from two-state transmission models. The demographic profile of PWUD has aged over time (mean age 35 years in 2004 vs. 44 in 2023) while injection prevalence remained stable (61.7% in 2023). Between 2013 and 2023, HCV burden declined substantially: HCV RNA prevalence fell from 19.4% (95% CI 16.3–23.0) to 9.5% (7.2–12.3) among all PWUD, and from 29.6% to 14.5% among PWID. Incidence among PWID decreased from 6.5 (4.1–8.0) to 2.4 (1.4–4.2) per 100 person-years. These reductions likely reflect the combined impact of DAA scale-up and harm reduction policies, marking substantial progress toward elimination targets. However, ongoing transmission highlights persistent barriers requiring targeted public health actions.

## Abstract (web summary)

Authors show in 2013-2023 France saw a substantial reduction in hepatitis C virus prevalence and incidence among people who use drugs. Widespread treatment and harm reduction drove the decline, bringing France closer to WHO 2030 elimination targets.

## Introduction

The World Health Organization (WHO) aims to eliminate hepatitis C virus (HCV) as a public health threat by 2030^1^, emphasizing the importance of reducing new infections and improving access to curative treatments. Despite the availability of direct-acting antivirals (DAA) treatments, which achieve sustained virological response rates exceeding 98%^2^, HCV remains a major global health challenge. This persistence stems from the lack of immunity following viral clearance or treatment, compounded by disparities in access to care^3^. As of 2022, an estimated 50 million people were living with chronic HCV infection (HCV RNA positive) worldwide, and approximately 240,000 deaths were attributed to the virus^4^.

While chronic HCV prevalence in the general European population is estimated at 0.5%^5^, the landscape shifts dramatically when looking at high-risk groups, especially people who inject drugs (PWID). Injecting drug use remains the primary driver of HCV transmission in high-income countries^6^, with PWID accounting for a significant proportion of chronic HCV cases—ranging from 19.4% to 70.0% across different European countries according to the last available estimates in 2019^5^. The general European population saw an estimated HCV incidence rate of around 7 per 100,000 individuals in 2019^7^. However, for PWID in Europe, a global meta-analysis estimated a much higher incidence of 9.5 per 100 person-years between 2015 and 2021^8^. Achieving WHO elimination targets requires incidence to drop below 5 per 100,000 in the general population and 2 per 100 person-years among PWID^1^.

In France, people who use drugs (PWUD) remain central to HCV control efforts^6,9^. However, up-to-date estimates of HCV prevalence and incidence are lacking, and data on long-term epidemiological trends remain limited. In the absence of longitudinal sero-epidemiological cohort studies, repeated cross-sectional surveys such as ANRS-Coquelicot surveys^10–12^ can provide critical epidemiological insights by combining behavioral data with biological testing results. Previous analyses of the 2004 and 2013 Coquelicot surveys^13^ focused solely on HCV antibody prevalence, as HCV RNA testing was not documented in 2004. The inclusion of HCV RNA testing in the 2023 survey^12^ allows for a comprehensive analysis across time points using both serological (anti-HCV) and molecular (HCV RNA) markers.

In this study, we use data from the three Coquelicot surveys (2004, 2013, and 2023), coupled with regression models and epidemic compartmental models to: (i) assess anti-HCV prevalence and incidence by age and survey edition, with a focus on recent trends in 2023; and (ii) leverage HCV RNA data to characterize active infections and infer changes in ongoing HCV transmission. Our findings contribute to evaluating progress towards HCV elimination in this key population.

## Results

### Demographics and drug use patterns

**Fig. 1:**
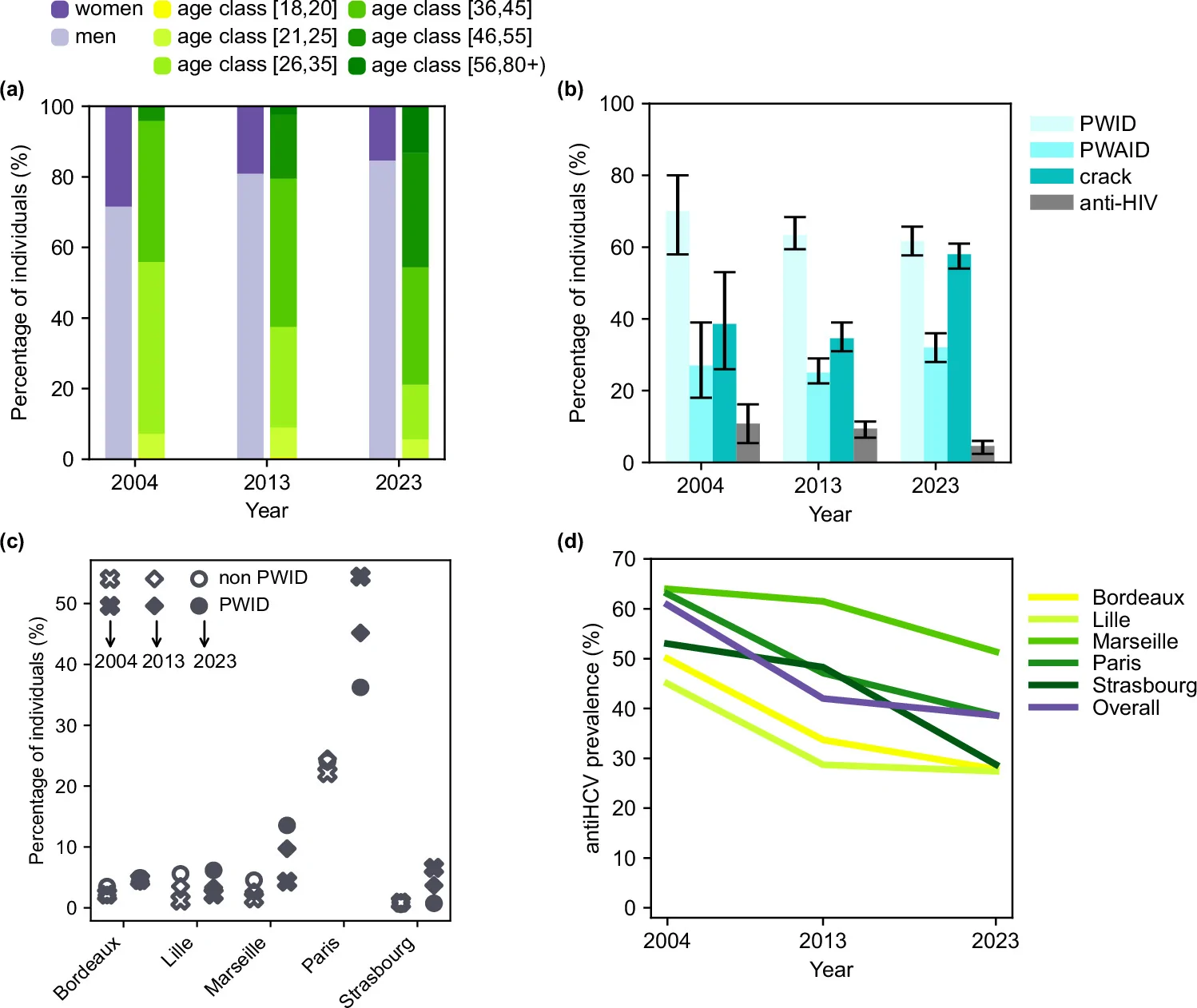
Descriptive statistics of three datasets. **a** Breakdown by gender (purple), and age groups (green). The mean values are displayed. **b** Bar plots indicating the prevalence of injecting drug users (PWID), active injecting drug users (PWAID), crack users, and anti-HIV across the years 2004, 2013, and 2023. The mean values are displayed; error bars represent 95% confidence intervals (CIs) accounting for the survey’s weighting design, based on samples of *n* = 573 (2004), *n* = 1307 (2013), and *n* = 1022 (2023) respondents. **c** Scatter plot comparing the percentage of injecting drug users (PWID) and non-PWID in five cities (Bordeaux, Lille, Marseille, Paris, and Strasbourg) for the years 2004, 2013, and 2023. The symbol shapes correspond to the year of the survey (crosses for 2004, diamonds for 2013, and circles for 2023). Injecting status is indicated by the fill color: filled symbols denote PWID, while void symbols represent non-PWID. The mean values are displayed. Percentages represent the distribution of the total sample by city and PWID status within each survey year. **d** Trends in anti-HCV prevalence (%) over time from 2004 to 2023, shown for different cities in France (Bordeaux, Lille, Marseille, Paris, Strasbourg) as well as overall (purple line). The mean values are displayed. **a**–**d** All estimations account for survey design. The 95% CIs are reported in Table S2 when not present here. PWID people who inject drugs, PWAID people who actively inject drugs, anti-HIV HIV antibodies, anti-HCV hepatitis C virus antibodies. Source data are provided as a Source Data file.

Between 2004 and 2023, the demographic profile of PWUD has continued to evolve. Men have consistently represented the majority, increasing from 71.6% [61.0, 80.2]% in 2004 to 84.6% [81.3, 87.5]% in 2023 (Fig. 1a, Table S2 of the Supplementary Information (SI)). The population has aged: while in 2004 most individuals were aged 26–35 years, the largest group shifted to 36–45 years by 2013, and by 2023, 45.6% of PWUD were aged 46 or older. Substance use patterns have evolved too, with cocaine crack use becoming more and more widespread, rising from 38.6% [26.1, 52.9]% in 2004 to 57.7% [53.4, 61.9]% in 2023 (Fig. 1b, Table S2). Injection drug use has remained the most common practice, with relatively stable prevalence: in 2013, 63.4% reported ever injecting and 25.1% had injected in the month preceding the interview; in 2023, these values were 61.7% and 31.6%, respectively. This pattern, with PWID representing the majority, has been observed across all five cities included in the analysis (Fig. 1c) and the gap between injectors and non-injectors appears more pronounced in larger cities such as Paris and Marseille.

### Estimated age- and time-dependent HCV prevalence

**Fig. 2:**
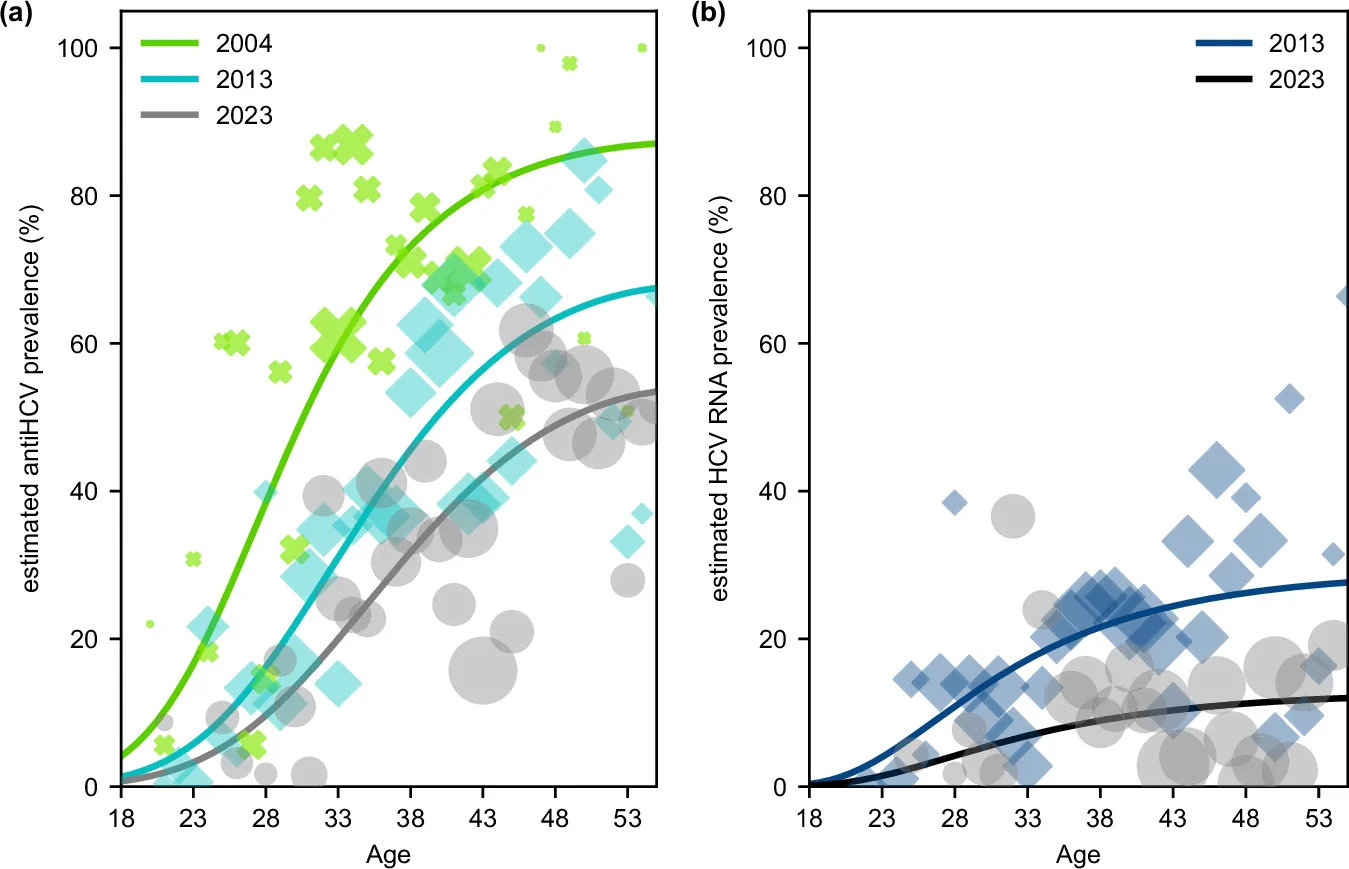
Prevalence of anti-HCV and HCV RNA in PWUD. **a** Anti-HCV prevalence by age, estimated from the regression model applied to PWUD in 2004 (green), 2013 (light blue), and 2023 (grey). Symbols indicate age-specific prevalence estimates: crosses for 2004, diamonds for 2013, and circles for 2023. **b** HCV RNA prevalence by age, estimated from the regression model applied to PWUD in 2013 (blue) and 2023 (black). Symbols indicate age-specific prevalence estimates: diamonds for 2013 and circles for 2023. **a, b** Confidence intervals are not shown for readability. Symbol size is proportional to survey weight. AntiHCV hepatitis C virus antibodies, PWUD people who use drugs, HCV hepatitis C virus. Source data are provided as a Source Data file.

Serological (anti-HCV) and molecular (HCV RNA) data were obtained via dried blood spot samples and analyzed in a central virology laboratory. After correction for HCV treatment (see “Methods” section), anti-HCV prevalence in PWUD was estimated at 60.8% in 2004 to 42.0% in 2013 and 38.6% in 2023 (Fig. 1d). City-specific data showed consistently higher prevalence in Paris and Marseille compared with the other cities throughout the study period (Fig. 1d). To estimate age- and time-dependent estimates of HCV prevalence in PWUD and PWID, we modeled anti-HCV and HCV RNA positivity using multivariate logistic regressions, where age effects were captured flexibly through fractional polynomials (see “Methods” section). By fitting the point-estimated data with multivariable regression models, we observe a significant association (*p* < 0.05) between age, time, and HCV prevalence (see Table 1). Specifically, antibody prevalence increases with age, rising steeply in younger age groups before reaching a plateau around the age of 50 (Fig. 2a). It is interesting to note that, regardless of age, antibody prevalence decreases between 2004, 2013, and 2023 (see also Discussion section, Section 5.1 of SI). A comparable age-dependent pattern is observed for HCV RNA positivity, which also increases with age. However, HCV RNA positivity does not plateau around the age of 50; instead, it continues to show a small but consistent increase in older age groups (Fig. 2b). Across all survey years, the difference between antibody and RNA prevalence remains substantial. Anti-HCV prevalence exceeded HCV RNA prevalence by approximately 23 percentage points in 2013 and 29 percentage points in 2023.

**Table 1:** Logistic regression models used to estimate anti-HCV and HCV RNA prevalence in drug user populations

Variable	Fractional polynomial transformation (η(a))	Regression coefficient (SE)	*p*-Value
Anti-HCV			
PWUD			
Age			<2e − 16
	${\left(a/10\right)}^{0.5}$	21.7 (4.3)	
	$\left(a/10\right)$	−4.5 (1.1)	
Edition 2013 (ref 2004)		−1.2 (0.2)	1.6e − 06
Edition 2023 (ref 2004)		−1.8 (0.3)	3.6e − 12
Intercept		−25.2 (4.7)	3.1e − 08
PWID			
Age			4.8e − 09
	${\left(a/10\right)}^{0.5}$	10.2 (1.7)	
	${\left(a/10\right)}^{2}$	−0.2 (0.1)	
Edition 2013 (ref 2004)		−1.3 (0.3)	7.8e − 05
Edition 2023 (ref 2004)		−2.0 (0.3)	2.4e − 09
Injecting drug use (ref No)		3.3 (0. 2)	<2e − 16
Intercept		−18.4 (2.6)	3.3e − 12
HCV RNA			
PWUD			
Age			0.000233
	${\left(a/10\right)}^{-2}$	−23.8 (14.3)	
	${\left(a/10\right)}^{-2}\bullet \log \left(a/10\right)$	14.8 (21.2)	
Edition 2023 (ref 2013)		−1.0 (0.2)	9.3e − 08
Intercept		−1.0 (0.9)	0.0001
PWID			
Age			0.000412
	${\left(a/10\right)}^{-2}$	−10.6 (3.0)	
Edition 2023 (ref 2013)		−1.1 (0.2)	5.5e − 08
Injecting drug use (ref No)		2.6 (0.4)	4.6e − 10
Intercept		−2.7 (0.4)	1.3e − 08

For example, for model Anti-HCV, PWUD for the survey edition 2023: ${{{\rm{logit}}}}\left(P\left(Y=1,|,a,t=2023\right)\right)=21.7\cdot {\left(a/10\right)}^{0.5}-4.5\cdot \left(a/10\right)-1.8-25.2$

Variables are presented in italics. *P*-values were derived from two-sided *t*-tests accounting for sampling weights and stratification. *SE* stands for standard error, *anti-HCV* hepatitis C virus antibodies, *HCV* hepatitis C virus, *PWUD* people who use drugs, *PWID* people who inject drugs.

### Estimated age- and time-dependent HCV incidence

**Fig. 3:**
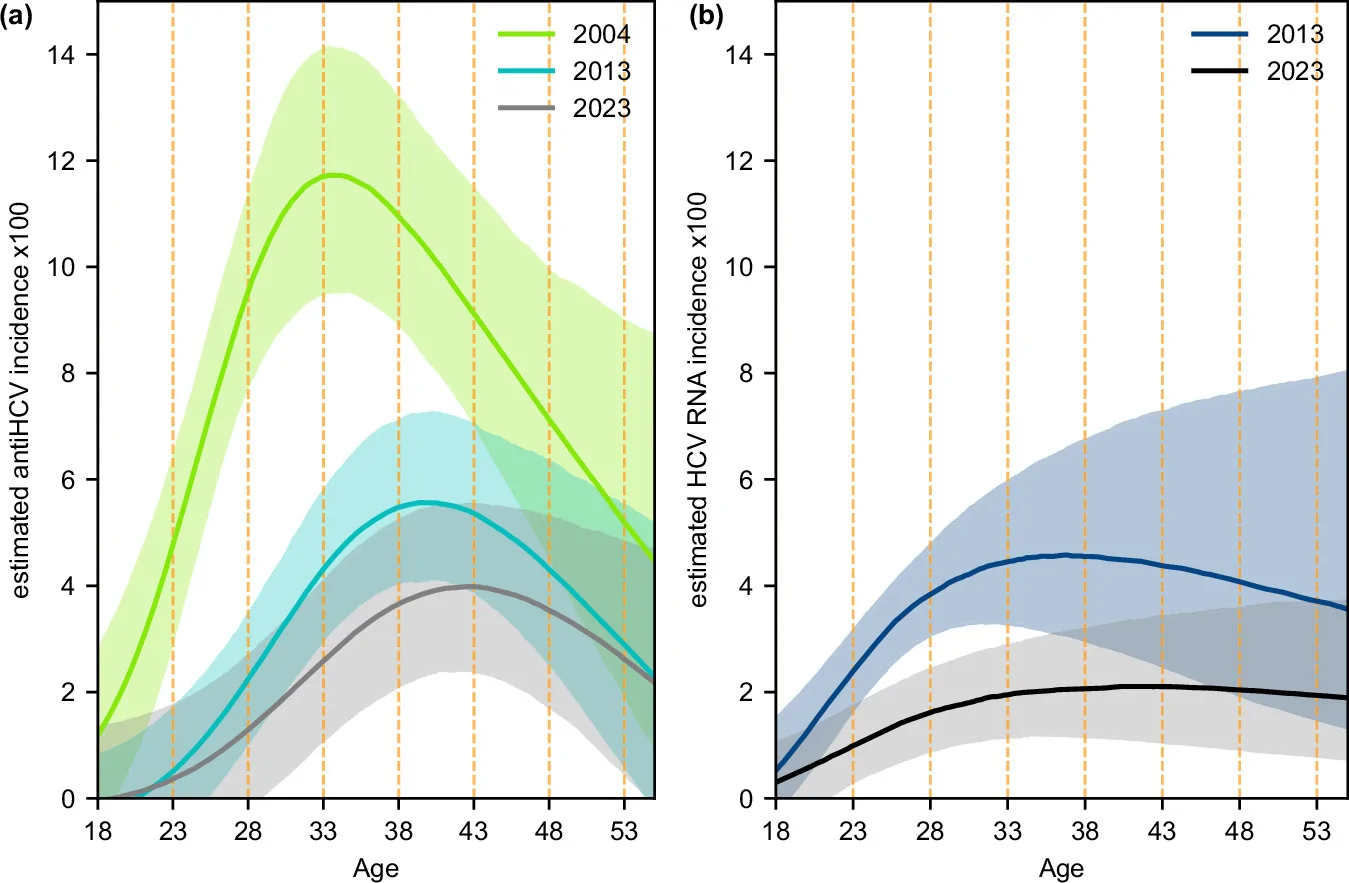
Incidence of anti-HCV and HCV RNA per 100 person-years in PWUD. **a** Anti-HCV incidence by age, estimated for 2004 (green), 2013 (light blue), and 2023 (grey). **b** HCV RNA incidence by age, estimated for 2013 (blue) and 2023 (black). **a**, **b** The mean values are displayed; shaded areas indicate 95% confidence intervals. Vertical orange lines are included as visual guides. AntiHCV hepatitis C virus antibodies, HCV hepatitis C virus. Source data are provided as a Source Data file.

To estimate incidence, we developed two compartmental models tailored to different biological markers: one capturing transitions between anti-HCV negative and anti-HCV positive states (accounting for seroreversion); and another focused on HCV RNA status (+/−) to better reflect active infection dynamics (Fig. S4). For the RNA-based model, spontaneous clearance and treatment-induced sustained virological response were included as clearance pathways. These models are consistent with previously developed frameworks^13–17^ (see “Methods”). We found that HCV antibody incidence declined over time. In 2004, 2013, and 2023, incidence increased with age until reaching a peak—at age 33 in 2004, age 40 in 2013, and age 42 in 2023—before subsequently declining (Fig. 3a). The highest estimated incidence was 11.6 [9.4, 14.0] per 100 person-years in 2004, reducing to 5.6 [4.1, 7.0] per 100 person-years in 2013 and to 4.0 [2.4, 5.4] per 100 person-years in 2023. For HCV RNA incidence, a slightly different pattern emerged. In 2013, incidence increased until age 36, peaking at 4.6 [3.1, 6.4] per 100 person-years before declining. By 2023, the peak incidence shifted to age 41, with a peak of 2.1 [1.1, 3.2] per 100 person-years.

### PWID-specific analysis

**Fig. 4:**
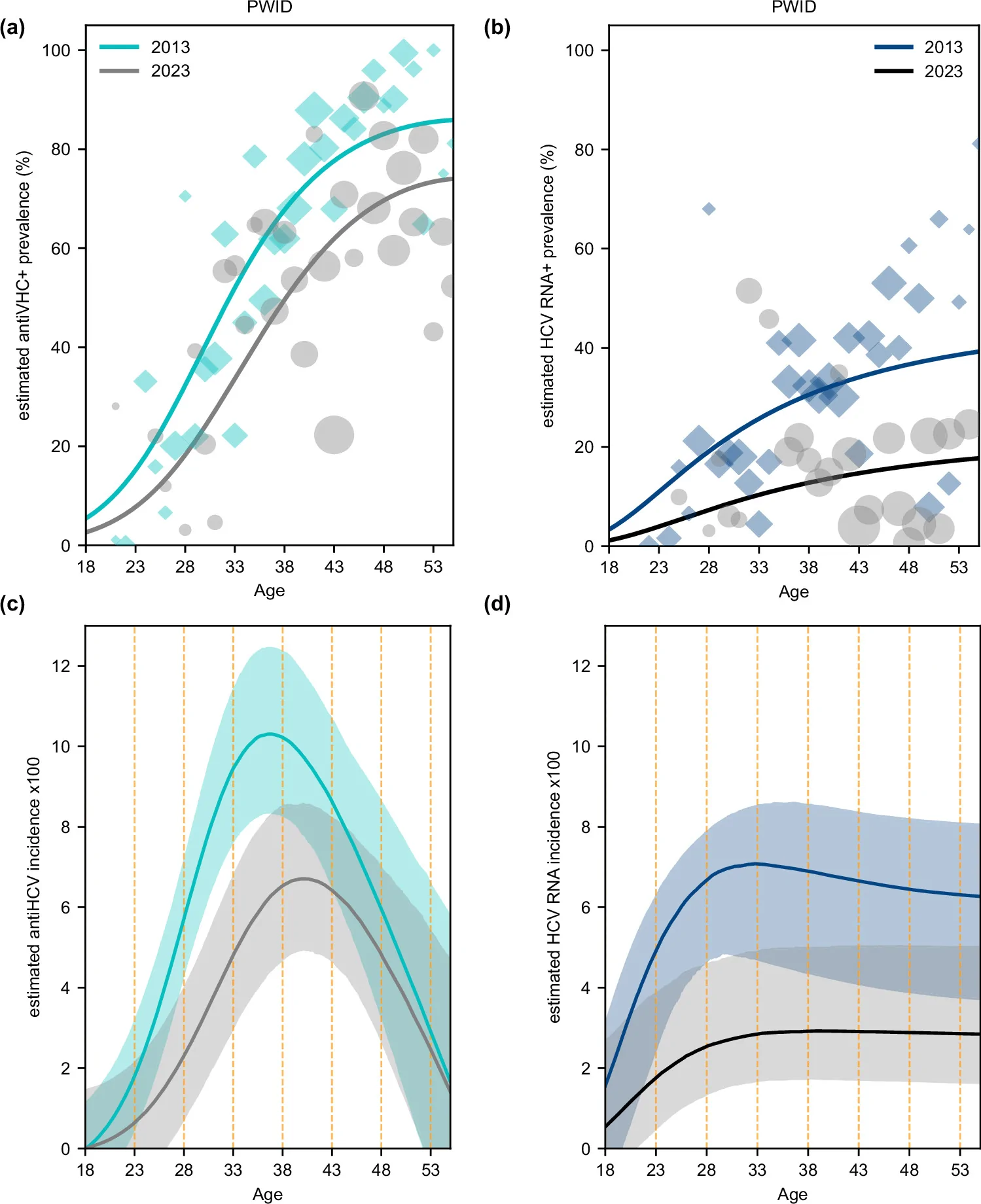
HCV RNA and anti-HCV prevalence and incidence in PWID. **a** Anti-HCV prevalence by age, estimated from the regression model applied to PWID in 2013 (light blue) and 2023 (grey). **b** RNA HCV prevalence by age, estimated from the regression model applied to PWID in 2013 (blue) and 2023 (black). **a**, **b** Confidence intervals are not shown for readability. Symbols represent age-specific prevalence estimates: diamonds for 2013 and circles for 2023. The size of the symbol is proportional to the survey weight. 2004 values for HCV antibody are reported in the SI (Fig. S4). **c** Anti-HCV incidence by age, estimated for 2013 (light blue) and 2023 (grey). **d** HCV RNA incidence by age, estimated for 2013 (blue) and 2023 (black). **c**, **d** The mean values are displayed; shaded areas represent 95% confidence intervals. Vertical orange lines serve as visual guides. AntiHCV hepatitis C virus antibodies, HCV hepatitis C virus, PWID people who inject drugs. Source data are provided as a Source Data file.

**Fig. 5:**
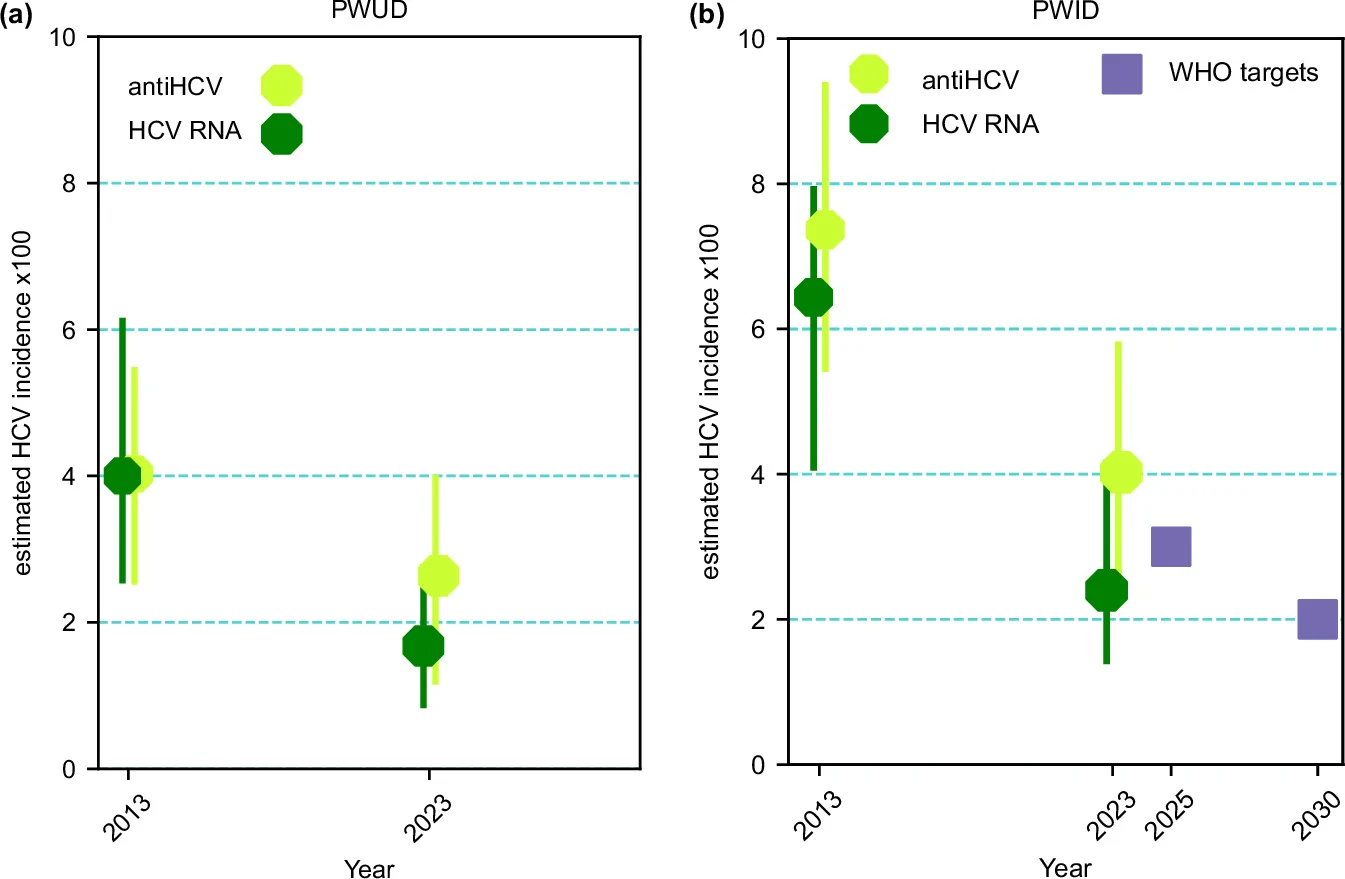
Global incidence of HCV in PWUD and PWID. **a** HCV incidence in PWUD for 2013 and 2023, estimated from anti-HCV (lighter green) and HCV RNA (darker green) data. **b** HCV incidence in PWID for 2013 and 2023, estimated from anti-HCV (lighter green) and HCV RNA (darker green) data. Purple squares represent the WHO eradication targets for 2025 and 2030^1^. **a**, **b** The mean values are displayed; vertical lines represent 95% confidence intervals, based on *n* = 573 (2004), *n* = 1307 (2013), and *n* = 1022 (2023) participants and *N* = 2000 bootstrap samples. Horizontal blue lines serve as visual guides. PWUD people who use drugs, PWID people who inject drugs, AntiHCV hepatitis C virus antibodies, HCV hepatitis C virus, WHO World Health Organization. Source data are provided as a Source Data file.

We also compared prevalence and incidence specifically among PWID in the last two surveys. In 2013 and 2023, anti-HCV prevalence also increased progressively with age, peaking among individuals in their late 40 s before reaching a plateau (Fig. 4a). A similar trend was observed for HCV RNA prevalence (Fig. 4b), although absolute levels remained lower than those of anti-HCV prevalence. In 2023, the overall prevalence of anti-HCV antibodies among PWID was 59.3% [54.1, 64.3]%, while the overall HCV RNA prevalence was 14.5% [10.9, 18.9]%. The HCV antibody incidence peaks were higher among PWID than PWUD, reaching 10.3 [8.4, 12.4] per 100 person-years in 2013 and 6.7 [5.0, 7.6] per 100 person-years in 2023 (Fig. 4c). The maximum incidence for HCV RNA was 7.1[4.7, 8.5] per 100 person-years in 2013 and 2.9 [1.7, 5.0] per 100 person-years in 2023. For HCV RNA, the incidence peaks at the age of 33. Sensitivity analyses suggest that the descending trend of 2023 remains robust despite uncertainty in the model parameters (Figs. S9 and S10). Globally, among all PWID, the HCV RNA incidence in 2023 is 2.4 [1.4, 4.2] per 100 person-years, while the anti-HCV estimate is at 3.1 [1.4, 4.7] per 100 person-years (Fig. 5b, Table S4). To achieve the WHO 2030 elimination target, an additional reduction of 16.7% is still required.

## Discussion

Based on data from three Coquelicot surveys conducted in France in 2004, 2013 and 2023, we estimated HCV prevalence and incidence by age and over time, among PWUD and PWID, using regression and epidemic transmission models. Our results indicate that France is on track to meet the WHO elimination targets by 2030, provided that the incidence of anti-HCV and HCV RNA among PWIDs in 2023 is compatible with the WHO milestones established for 2025^1^. Notably, HCV RNA incidence decreased from a mean value of 6.4 to 2.4 per 100 person-years between 2013 and 2023, representing an almost threefold reduction. Achieving the 2030 target of 2 per 100 person-years incidence would therefore require only an additional reduction of 17%. This aligns with other studies, which suggest that the WHO elimination target is realistic for (and primarily achievable by) some high-income countries, including France^18–20^.

The Coquelicot surveys enabled these estimates by targeting a hard-to-reach and often precarious population across multiple urban areas and specialized treatment services. The surveys in 2013 and 2023 editions further strengthened surveillance and epidemiological tracking through inclusion of non-French speaking population and integration of HCV RNA testing. We provide age-specific estimates of HCV prevalence and incidence among PWUD, for both HCV-antibody and HCV RNA. Our previous study, conducted in 2016^13^, estimated HCV incidence between 2004 and 2011 but it relied solely on HCV-antibody data and it was conducted before the introduction of DAA. An earlier study from the late 1990s^21^ estimated anti-HCV incidence in northeastern France, but it was limited to a two-year period, to a limited part of France and focused exclusively on people who inject drugs. In contrast, our study includes a large and heterogeneous sample of participants recruited across multiple French cities over 20-years period.

The progress observed in France reflects the encouraging—yet heterogeneous—trends reported across Europe and other high-income countries regarding HCV elimination among PWID^18,22^. Notably, several cities in Spain^23,24^ and Norway^25,26^ have achieved remarkable reductions in HCV prevalence. In Oslo HCV RNA prevalence among PWIDs decreased from 26.3% in 2018 to 14.2% in 2021, reaching 7% in 2023, thus meeting the WHO’s 80% reduction target. These achievements have been attributed to integrated approaches combining prevention, testing, harm reduction and treatment for PWID. France stands out for its widespread harm reduction services^27^ and broad access to DAA treatments^28^, supported by public funding for health infrastructure. Nevertheless, marked heterogeneity persists across Europe^18,22^, with HCV RNA prevalence among PWUD estimated at 56% in Tallinn (2022), 27% in Bavaria (2022), and 30% in Stockholm (2021). France’s estimated HCV incidence of 2.4 (1.4–4.2) per 100 person-years in 2023 aligns with the downward trends observed^25,29–32^, where infection and reinfection incidence between 2013 and 2023 ranges from 1.2 to 3.7 per 100 person-years. Modeling studies from England further estimate an HCV incidence in 2023 between 1.9 and 4.9 per 100 person-years, with projections suggesting a decline to 0.2–2.2 per 100 person-years by 2030. In Scotland, a modeling study estimated that the total infection incidence rate decreased to 2.8 per 100 person-years in 2019–2020 and to 3.4 per 100 person-years in 2022–23^33^.

Compared to other global modelling approaches^8,34^, the strength of our study is to provide more precise, representative and up-to-date estimates of HCV prevalence and incidence in France. It relies on a strong sampling methodology as well as individual-level biomarker data from 2023, which enhances estimation accuracy. Unlike global estimates aimed at international comparability, our country-specific approach is tailored to meet local needs and enables direct estimation for key subpopulations (e.g. PWID) and geographic strata. The observed age-dependency in incidence likely reflects both the aging and mortality of individuals with HCV antibodies, and a lower rate of new infections among recent PWUD. Younger PWUD are also less exposed to older ones, who have historically acted as reservoirs of transmission. Many young people began injecting at a time when access to sterile equipment and harm reduction services was more widespread. This is particularly true in France, where harm reduction policies were introduced relatively late (in the 1990s^35^) but have since benefited from substantial and consistent investment from the mid-1990s to the present^27^.

To sustain this trend and avoid resurgence or infection peaks, it is crucial that access to care and harm reduction remain a high priority. A proactive, comprehensive approach to prevention, screening and harm reduction is essential^34,36^, along with efforts to prevent reinfection among those previously treated^37^. Evidence suggests that the benefits of DAA will only be fully maximized with an exceptional increase in diagnosis coverage to 90% by 2030^38^. This requires harm reduction programs aimed directly at reducing incidence, while anti-viral treatment focuses on reducing chronic prevalence^39^. In France, as of 2023, access to DAA remains limited for individuals without social health coverage—a group estimated to represent 20.5% [18.2%–23.0%] of PWUD^12^. Our findings also indicate that the majority of HCV infections occur among PWID, consistent with previous estimates for high-income countries^13,40^. This population has consistently represented the majority of HCV cases, underscoring the need for targeted interventions. An area of concern is the provision of needles and syringes for safe drug use: about 1 in 4 PWID report inadequate availability to meet their needs in 2023^12^. This highlights potential gaps to prevent new infections and reinfections.

Several factors can influence temporal trends in HCV prevalence and incidence. This includes strong associations with demographic and behavioral characteristics (Table S2), as well as structural factors such as drug and health policies. Moreover, our analysis relies on temporal snapshots, from which we infer trends. However, we lack data for the years when no surveys were conducted, leaving gaps in our understanding of potential fluctuations. We do not know the full impact of the COVID-19 pandemic, during which efforts to sustain the HCV care cascade were disrupted, and many micro-elimination initiatives were hindered^41^. However, the three Coquelicot surveys span two decades marked by major transformations in HCV care^42^, including the introduction of DAA and expanded harm reduction access. As such, they offer a valuable long-term insight on incidence and prevalence dynamics. Migration also plays a role in shaping trends, potentially introducing new sources of infection. Since the mid-2000s, PWID from Eastern Europe (especially from Georgia) have migrated to France. In 2013, HCV seroprevalence among Russian-speaking drug users was nearly double that of French-speaking peers (9 out of 10 were anti-HCV positive)^43^. Similarly, in migrants from North African countries, the incidence ranges from 4.5 to 15.5 per 100 person-years in 2024^44^. This evidence motivated the inclusion of migrant population in our analysis.

The decline in HCV antibody prevalence observed between 2004, 2013 and 2023 should be interpreted cautiously and likely reflects the combined effect of several epidemiological and methodological factors. Most of the decrease occurred between 2004 and 2013, whereas the difference between 2013 and 2023 was comparatively modest, particularly after correcting prevalence estimates for previously treated individuals who tested anti-HCV negative. The estimates for the latter survey waves remain broadly consistent with observations reported in other European settings^22^. In addition, generational changes among people who use drugs may have contributed to lower cumulative exposure to HCV over time, through increased awareness of transmission risks, broader access to harm reduction interventions, and safer injecting practices. At the same time, individuals with longstanding HCV infection, who likely contributed substantially to the high antibody prevalence observed in 2004, may no longer be represented in later surveys because of aging, mortality, or cessation of drug use. The Coquelicot surveys are repeated cross-sectional studies rather than a longitudinal cohort; the observed temporal changes may also reflect differences in the composition of the sampled PWUD population across survey waves.

In this study, we analyzed both molecular (HCV RNA) and serological (HCV antibody) data, as each provides distinct epidemiological insights. Antibody-based estimates capture the prevalence of individuals who have ever been exposed to HCV, regardless of their current infection status. However, this approach tends to overestimate the true prevalence of active infections, by including individuals who have cleared the virus (anti-HCV+ and HCV RNA−, see Table S1). At the same time, recent infections may be missed, as individuals in the early stages of infection can be RNA-positive while still seronegative. This limitation becomes particularly important following the scale-up of DAA treatment, which substantially reduces active viraemic infection while having little impact on antibody prevalence. We therefore focused on RNA-based modeling, which directly captures active infections. In this approach, individuals are considered susceptible if they are RNA-negative, regardless of their serological status. This framework accounts for the possibility of reinfection, as antibody-positive individuals remain at risk. One alternative metric could have been the incidence of first-time infections, identified by individuals who are RNA-positive but antibody-negative (with rare cases of seroreversion). However, we did not pursue this approach due to the very small number of individuals with this profile—approximately 10 in both surveys (Table S1). Moreover, this biomarker’s condition is transient, as it only occurs during the early stage of infection. The discrepancy between RNA and antibody-based incidence estimates in 2023 (a 22.5% relative variation) is markedly greater than in 2013, when estimates were—6.4 and 6.5 per 100 person-years- reflecting only a 1.5% variation. The increasing divergence between these two indicators over time may reflect improvements in access to treatment, particularly the rollout of DAA, which reduces chronic infection without affecting seropositivity. The RNA becomes undetectable, but antibodies remain as part of the immune memory.

This study has several limitations. First, the cross-sectional design of our surveys limits our ability to assess changes in HCV dynamics over time and to infer causality to explain variations. However, repeated cross-sectional surveys combined with rigorous mathematical modeling, offer a practical alternative to long-term prospective cohort studies, which are time- and resource-intensive. By integrating routine surveillance data into model input parameters, it is possible to derive indirect yet informative estimates of incidence. Cohort studies also face important limitations, particularly regarding representativeness and selection biases. Our recruitment strategy relied on a broad network of specialized services, spanning low- and high-threshold facilities, as well as outreach and street-based teams aimed at reaching highly marginalized PWUD. While these approaches are expected to capture a substantial proportion of the PWUD population, selection bias may persist, as individuals who are more socially integrated, less vulnerable, or only occasional users may be less likely to attend such services. Second, we effectively modeled active infections, impact of treatments and the spontaneous clearance, which may introduce uncertainties. However, we have tested the robustness of these assumptions through sensitivity analyses. Our model also assumes that individuals cured by DAA treatment re-enter the susceptible compartment with unchanged re-infection risk. However, DAA scale-up is often paired with harm reduction strategies (e.g., syringe services and OAT) and education that may lower re-infection rates^45^. Conversely, successful treatment could increase the pool of susceptible individuals, potentially leading to higher re-infection rates if risk behaviors persist or re-testing declines, as suggested in previous studies^33,46^. Future extensions of the model could explore heterogeneous re-infection risks to better reflect empirical observations. Additionally, some model parameters relied on self-reported data, such as the percentage of individuals receiving treatment over the years. Finally, incorporating stratified parameters by age would have strengthened the model. However, detailed demographic data on the French PWUD population are currently lacking.

Our analysis highlights the progress made in reducing both incidence and prevalence over time, aligning with WHO eradication criteria. Our findings provide valuable insights into the burden of HCV within the PWUD population and can inform the development of medium-term management plans.

## Methods

### Ethical approval

All procedures involving human participants were conducted in accordance with the 1964 Helsinki declaration and its later amendments. The research project has been approved by ethical review boards for each edition of the ANRS-Coquelicot Survey, CCPPRB in 2004, CPP-Créteil in 2011–2013 and CPP-Ile de France II in 2022–2023. Interviewers provided participants with both oral and written information about the study prior to obtaining their consent. Oral consent was requested to participants due to the illegality of drug use practices in France. Participants received an incentive of 15 euros for their participation.

### Data ANRS-coquelicot surveys

We analyzed data from three cross-sectional multicenter epidemiological surveys in 2004, 2013, and 2023^10–12^. The surveys targeted individuals who had injected or snorted drugs at least once, as these practices are considered a risk for HCV transmission. In the 2013 and 2023 editions, the inclusion criteria were expanded to also include individuals who had smoked crack cocaine at least once, reflecting the increasing importance of this route of administration in the French context^12^ as well as its role in HCV transmission^47^ (see Supplementary Information; SI). Recruitment took place through various low-threshold services, including needle exchange programs, drug treatment centers, and post-treatment facilities. Participants had to be at least 18 years old and provide oral consent to participate. Data collection was conducted through face-to-face interviews. The questionnaire covered a wide range of topics, including sociodemographic, drug-use history, risk behaviors, and access to healthcare and prevention services. We analyzed key variables associated with HCV infection^13^: these include age, HIV serostatus, and injection practices. We defined a person who injects drugs (PWID) as someone who had injected drugs at least once in his/her lifetime, while a person who actively injects drugs (PWAID) was defined as someone who had injected drugs in the month preceding the interview. The 2004 survey^10^ took place in five cities—Bordeaux, Lille, Marseille, Paris, and Strasbourg—across 136 participating services, enrolling 1,462 PWUD. The questionnaire was available only in French. The 2013 survey^11^ expanded its geographic coverage to include two additional departments, with recruitment across 121 services and with a questionnaire available in French and Russian. This edition included 1568 participants. The 2023 survey^12^ further extended its geographical scope to 27 French cities, with recruitment in 195 services, ultimately enrolling 2125 participants^12^. The questionnaire was also translated in Georgian, Arabic, and English. Sampling for all surveys employed a probabilistic time-location method^48^ with a two-stage design. In the first stage, time-service slots for surveys were selected using simple random sampling without replacement. In the second stage, individuals within these slots were recruited using systematic random sampling. To produce all population-level estimates for PWUD, the analyses accounted for the sampling design. This included the use of sampling weights (SI), stratification, and primary sampling units across all three surveys (SI).

### Biological samples analysis

Biological samples collected using dried blood spots were available for 79%, 92%, and 82% of participants in 2004, 2013, and 2023, respectively. Participants provided self-collected blood samples on blotting paper, which were sent to a centralized virology laboratory. The laboratory performed serological testing for the presence of HIV and HCV antibodies in all three surveys, and molecular testing for HCV RNA in 2013 and 2023 only. Valid laboratory results were obtained for 56%, 85% and 80% of the total participants across the three survey waves, respectively. Further details of the molecular and serological data analysis are provided in the SI.

### Anti-HCV data correction

To correct HCV serological data, we redefined anti-HCV positivity for 2013 and 2023 to include individuals with either a positive anti-HCV test result or a self-reported history of HCV treatment, following what was previously done in ref. ^49^. Treatment history data were unavailable for the 2004 survey; however, this limitation is unlikely to affect interpretation, as HCV treatment access in France was extremely limited during that period^50^. The impact of the correction on prevalence estimates is shown in Figure S2.

### Combining datasets

To conduct prevalence and incidence analyses, we combined the three surveys into a single dataset. To ensure comparability across populations, the analysis was restricted to data from the five cities common to all survey waves—Bordeaux, Lille, Marseille, Paris, and Strasbourg —and to PWUD with available and exploitable blood samples. The data from the three surveys were combined using a pooling approach^51^, which involved adjusting sampling weights to reflect the target population and account for the precision of each survey^51–53^. The sampling weight (${w}_{{year}}^{i}$) for an individual i in year ${w}_{{{\mathrm{year}}}}^{i}$ is multiplied by the combination coefficient for that year (${{\mathrm{year}}}$):

1 $${w}_{{{\mathrm{final}}}}^{i}={w}_{{{\mathrm{year}}}}^{i}\cdot {\alpha }_{{{\mathrm{year}}}}$$

where ${\alpha }_{{{\mathrm{year}}}}$ are calculated to minimize the variance of the final estimator^54–56^ and can be determined as follows:

2 $${\alpha }_{{{\mathrm{year}}}}=\frac{1/{{{\mathrm{deff}}}}_{{{\mathrm{year}}}}}{1/{{{\mathrm{deff}}}}_{2004}+1/{{{\mathrm{deff}}}}_{2013}+1/{{{\mathrm{deff}}}}_{2023}}$$

where ${{{{\rm{deff}}}}}_{{{{\rm{year}}}}}$ represents the design effect for each dataset ${{{{\rm{deff}}}}}_{{{{\rm{year}}}}}=1+{\left(\frac{{{{{\rm{CV}}}}}_{{{{\rm{year}}}}}}{100}\right)}^{2}{{{\rm{and}}}}\,{{{{\rm{CV}}}}}_{{{{\rm{year}}}}}=\frac{{{{\rm{SD}}}}({w}_{{{{\rm{year}}}}})}{{\bar{w}}_{{{{\rm{year}}}}}}\cdot 100$

with ${{{\rm{SD}}}}\left({w}_{{{{\rm{year}}}}}\right)$ and ${\bar{w}}_{{{{\rm{year}}}}}$ are the standard deviation and the mean of the sampling weights ${w}_{{{\mathrm{year}}}}^{i}$ for individuals *i* in that year. This method prioritizes surveys with lower weight dispersion and higher precision by assigning greater weight to datasets that minimize variance.

### Modelling prevalence

#### Anti-HCV

Let us consider a binary variable of interest Y, corresponding to the HCV antibodies classification (${Y}_{i}=1$ if individual *i* is seropositive and 0 if not). To estimate the probability of being seropositive for anti-HCV ${{{\rm{P}}}}(Y=1{|a},t)$, where *a* represents age and *t* denotes the survey edition (2004, 2013, or 2023), we used a multivariate regression model with a logit link function. Age was treated as a continuous variable, and its association with anti-HCV prevalence was modeled using fractional polynomials^57^, allowing for flexible, nonlinear relationships. The model was specified as follows:

3 $${{{\rm{P}}}}\left(Y=1,|,a,t\right)=\frac{\exp (\alpha+\eta \left(a\right)+{ct})}{1+\exp (\alpha+\eta \left(a\right)+{ct})}$$

Where α is the intercept, *c* is the regression coefficient associated with the survey edition *t* (2004, 2013, or 2023), and *η(a)* represents the fractional polynomial for age *a*. We tested several models incorporating both age and survey edition as covariates, including alternative models that used splines^58^ instead of fractional polynomials, and models with interactions between age and survey edition (see SI). The best-fitting model, identified through AIC model comparison, is reported in Table 1.

#### HCV RNA

We estimate the probability of testing positive for HCV RNA, ${{{\rm{I}}}}(Y=1{{{\rm{| }}}}a,t)$, where *a* represents age and *t* denotes the survey edition (2013, or 2023). We applied the multivariate regression model described above to evaluate the association between age, survey edition, and HCV RNA prevalence. The best-fitting model, identified through AIC model comparison, is reported in Table 1.

### Modelling incidence

#### Anti-HCV

We modeled the transmission of HCV in PWUD and PWID populations using a two-state compartmental model, with the two states being anti-HCV negative (*N*) and anti-HCV positive (*P*), as previously done in ref. ^13^. Following exposure, individuals in state *N* can become infected and transition to state *P* at a rate *λ(a, t)* that depends on age *a* and time *t*. Transitions from state (*P)* back to state (*N)* may also occur due to loss of detectable anti-HCV antibodies. Here, *λ(a, t)* represents the incidence of HCV infection, including *primo-infection* and rare cases of reinfection in individuals who have undergone complete seroreversion^59–61^ (Section 4 SI). *λ(a, t*) can thus be expressed as a function of the derivative of the prevalence function. Parameters, values, and sources used to define the compartmental models are listed in Table 2. In particular, the percentages reported in Table 2 for the different treatment PWUD received (interferon/ribavirin, direct-acting antivirals) were derived from weighted calculations based on self-reported responses in our dataset. Sensitivity analyses on parameters are reported in the SI. All calculations are provided in SI. We computed the confidence intervals of the estimates using a bootstrap method, as described in SI.

**Table 2:** Parameters of the two-state compartmental models

Variable	Anti-HCV model	HCV RNA model	References
*β*(*t*): yearly proportion new PWUD	2% in 2004 2% in 2013 1.6% in 2023	2% in 2004 2% in 2013 1.6% in 2023	^66,67^
*ε*: seroreversion rate after spontaneous clearance	0.001 years^−1^	–	^60^
*ε*‘: seroreversion rate after SVR (IFN or DAA)	0.0048 years^−1^	–	^61^
*x*(*t*): PWUD that already received a IFN treatment	0% in 2004 18% in 2013 9% in 2023	0% in 2004, 18% in 2013 9% in 2023	Calculation from Ref. ^11^ Calculation from Ref. ^12^
*z*(*t*): PWUD that already received a DAA treatment	0% in 2004 0% in 2013 23% in 2023	0% in 2004 0% in 2013 23% in 2023	Calculation from Ref. ^11^ Calculation from Ref. ^12^
*δ*: proportion of spontaneous clearance in acute phase	–	24.4%	^68^
*γ*: spontaneous clearance rate in chronic phase	–	0.0019 year^−1^	^67^
${{{\rm{\gamma }}}}{\prime}$*:* proportion of SVR after IFN treatment	–	60%	^16,69–71^
*τ*’^−1^: average treatment duration^a^	–	0.5 years	^16^
${{{\rm{\gamma }}}}{\prime} {\prime}$*:* proportion of SVR after DAA treatment	–	98%	^2^
*τ*”^−1^: average treatment duration	–	0.23 years (12 weeks)	^2,38^
*µ*_1_: death rate among PWUD	0.022 years^−1^	0.022 years^−1^	
σ: death rate among HCV positive PWUD	0.03 years^−1^	0.03 years^−1^	^72–75^

^a^Exit rate calculated from the average of the genotype 1 treatment and the genotype 2 treatment length. Variables are presented in italics.

*Anti-HCV* hepatitis C virus antibodies, *HCV* hepatitis C virus.

#### HCV RNA

As the number of patients successfully treated with DAA increases over time^62^, antibody prevalence becomes a less reliable indicator of the HCV burden. A more accurate measure is the prevalence of chronic and/or current infections among drug users, determined using HCV RNA tests to confirm active viral presence^63^. To align with this paradigm shift, we modified our model by estimating incidence based on individuals with positive HCV RNA results, rather than those with positive antibodies. This approach maps seronegative and seropositive individuals into susceptible and infected individuals (Fig. S4). Given the cross-sectional nature of our studies, we cannot distinguish between acute and chronic infections. Thus, we use a single compartment for infected individuals but accounting for spontaneous clearance during the acute phase and both clearance and sustained virological response (SVR) during the chronic phase. Further details can be found in Table 2, Fig. S4, Fig. S5 and section 4 of SI.

### Global incidence

We estimated the incidence of anti-HCV and HCV RNA infection in 2004, 2013, and 2023 within the PWUD and PWID population. The proportion of PWUD at age *a* and time *t*, denoted as $\hat{q}(a,t)$, was estimated using the Horvitz-Thompson estimator^64^:

4 $$\hat{q}(a,t)=\frac{{\sum }_{i=1}^{n}{w}_{i}{x}_{i}(a,t)}{{\sum }_{i=1}^{n}{w}_{i}}$$

Where ${w}_{i}$ represents the survey weight of individual $i,{x}_{i}\left(a,t\right)=1$ if the individual is of age a at time t, and 0 otherwise, and n is the sample size. For each survey, the overall incidence can then be defined as the weighted arithmetic mean of age-specific incidences:

5 $$\lambda \left(t\right)={\sum}_{a}\hat{q}(a,t)\lambda (a,t)$$

### Reporting summary

Further information on research design is available in the Nature Portfolio Reporting Summary linked to this article.

## Supplementary information

Supplementary Information

Reporting Summary

Peer Review file

## Source data

Source Data

## Data Availability

ANRS-Coquelicot data are confidential and cannot be shared due to ethical restrictions. Researchers wishing to have more information about the data provided in the article are invited to contact Marie Jauffret-Roustide (PI of the survey) and Chiara E. Sabbatini, at the following addresses: marie.jauffret-roustide@inserm.fr; chiara.sabbatini@santepubliquefrance.fr. All aggregate results, prevalence, and incidence data generated in this study are included in the accompanying Source Data file. Source data are provided with this paper.

## Code availability

Analyses were carried out in R version 4.3.3 and STATA 18.0. Code of the model is publicly available at https://codeberg.org/SpF_DATA/HCV_among_PWUD_Coquelicot.git For reproducibility, the version corresponding to this study is deposited in Zenodo (https://zenodo.org/records/22054853)^65^.

## References

[1] WHO. Global health sector strategies on HIV, viral hepatitis and sexually transmitted infections for the period 2022-2030. https://www.who.int/publications/i/item/9789240053779

[2] Forns, X. et al. Glecaprevir plus pibrentasvir for chronic hepatitis C virus genotype 1, 2, 4, 5, or 6 infection in adults with compensated cirrhosis (EXPEDITION-1): a single-arm, open-label, multicentre phase 3 trial. *Lancet Infect. Dis.* **17**, 1062–1068 (2017). 10.1016/S1473-3099(17)30496-6 28818546

[3] WHO. Global hepatitis report 2024: action for access in low- and middle-income countries. https://www.who.int/publications/i/item/9789240091672

[4] WHO. Hepatitis C. https://www.who.int/news-room/fact-sheets/detail/hepatitis-c

[5] Thomadakis, C. et al. Prevalence of chronic HCV infection in EU/EEA countries in 2019 using multiparameter evidence synthesis. *Lancet Reg. Health Eur.* **36**, 100792 (2024). 10.1016/j.lanepe.2023.100792 PMC10769889 38188273

[6] ECDC. Hepatitis C, Annual Epidemiological Report for 2023. https://www.ecdc.europa.eu/sites/default/files/documents/AER-Hepatitis-C-2023.pdf

[7] Simão, M. & Gonçalves, C. Hepatitis C virus infection in Europe. *Pathogens* **13**, 841 (2024). 10.3390/pathogens13100841 PMC11510056 39452713

[8] Artenie, A. et al. Global, regional, and national estimates of hepatitis C virus (HCV) infection incidence among people who inject drugs and number of new annual HCV infections attributable to injecting drug use: a multi-stage analysis. *Lancet Gastroenterol. Hepatol.* **10**, 315–331 (2025). 10.1016/S2468-1253(24)00442-4 PMC12179389 39993400

[9] Brouard, C., Laporal, S., Bruyand, M., Pillonel, J. & Lot, F. Hépatites B ET C MISE À JOUR DES DONNÉES ÉPIDÉMIOLOGIQUES. *Journée Nationale De Lutte Contre Les Hépatites B et C, Ministère Des Solidarités Et De La Santé*, *Paris*, *25 Septembre**2019*.

[10] Jauffret-Roustide, M. et al. A national cross-sectional study among drug-users in France: epidemiology of HCV and highlight on practical and statistical aspects of the design. *BMC Infect. Dis.* **9**, 113 (2009). 10.1186/1471-2334-9-113 PMC2733898 19607712

[11] Weill-Barillet, L. et al. Hepatitis C virus and HIV seroprevalences, sociodemographic characteristics, behaviors and access to syringes among drug users, a comparison of geographical areas in France, ANRS-Coquelicot 2011 survey. *Rev. Épidémiol Publiq* **64**, 301–312 (2016). 10.1016/j.respe.2015.10.003 26904917

[12] Jauffret-Roustide, M. Monitoring HIV, HCV and HBV among people who use drugs in France. A reflective feed-back on a 25-year experiment based on mixed methods. The ANRS-Coquelicot survey. *Int. J. Drug Policy* **136**, 104690 (2024). 10.1016/j.drugpo.2024.104690 39740356

[13] Leon, L. et al. Age- and time-dependent prevalence and incidence of hepatitis C virus infection in drug users in France, 2004–2011: model-based estimation from two national cross-sectional serosurveys. *Epidemiol. Infect.* **145**, 895 (2016). 10.1017/S0950268816002934 PMC9507806 28004616

[14] Pitcher, A. B., Borquez, A., Skaathun, B. & Martin, N. K. Mathematical modeling of hepatitis C virus (HCV) prevention among people who inject drugs: a review of the literature and insights for elimination strategies. *J. Theor. Biol.* **481**, 194 (2018). 10.1016/j.jtbi.2018.11.013 PMC6522340 30452959

[15] Cousien, A. et al. Dynamic modelling of hepatitis C virus transmission among people who inject drugs: a methodological review. *J. Viral Hepat*. **22**, 213–29 (2015). 10.1111/jvh.12337 25270261

[16] Martin, N. K. et al. Can antiviral therapy for hepatitis C reduce the prevalence of HCV among injecting drug user populations? A modeling analysis of its prevention utility. *J. Hepatol.* **54**, 1137–1144 (2011). 10.1016/j.jhep.2010.08.029 21145810

[17] Martin, N. K., Vickerman, P. & Hickman, M. Mathematical modelling of hepatitis C treatment for injecting drug users. *J. Theor. Biol.* **274**, 58–66 (2011). 10.1016/j.jtbi.2010.12.041 21236265

[18] EUDA (The European Union Drugs Agency). Drug-related infectious diseases – the current situation in Europe (European Drug Report 2025) |. https://www.euda.europa.eu/publications/european-drug-report/2025/drug-related-infectious-diseases_en#0-level-3

[19] European Union Drugs Agency (EUDA): Viral hepatitis elimination barometer among people who inject drugs in Europe. https://www.euda.europa.eu/publications/data-factsheet/viral-hepatitis-elimination-barometer-among-people-who-inject-drugs-in-europe_en

[20] Razavi, H., Sanchez Gonzalez, Y., Yuen, C. & Cornberg, M. Global timing of hepatitis C virus elimination in high-income countries. *Liver Int. J. Int. Assoc. Study Liver* **40**, 522–529 (2020). 10.1111/liv.14324 31815353

[21] Lucidarme, D. et al. Incidence and risk factors of HCV and HIV infections in a cohort of intravenous drug users in the North and East of France. *Epidemiol. Infect.* **132**, 699–708 (2004). 10.1017/s095026880400247x PMC2870151 15310172

[22] EUDA (The European Union Drugs Agency). Viral hepatitis elimination barometer among people who inject drugs in Europe. https://www.euda.europa.eu/publications/data-factsheet/viral-hepatitis-elimination-barometer-among-people-who-inject-drugs-in-europe_en

[23] Mochales, A. H. et al. Implementing a new HCV model of care for people who use drugs. *JHEP Rep.* **6**, 101145 (2024). 10.1016/j.jhepr.2024.101145 PMC11416665 39308984

[24] Ryan, P. et al. Decrease in active hepatitis C infection among people who use drugs in Madrid, Spain, 2017 to 2023: a retrospective study. *Eurosurveillance* **29**, 2300712 (2024). 10.2807/1560-7917.ES.2024.29.29.2300712 PMC11258947 39027941

[25] Whittaker, R., Midtbø, J. E. & Kløvstad, H. Monitoring progress towards the elimination of hepatitis C as a public health threat in norway: a modelling study among people who inject drugs and immigrants. *J. Infect. Dis.* **230**, e700 (2024). 10.1093/infdis/jiae147 PMC11420790 38537267

[26] Opheim, E. et al. Towards elimination of hepatitis C in Oslo: cross-sectional prevalence studies among people who inject drugs. *Int. J. Drug Policy* **123**, 104279 (2024). 10.1016/j.drugpo.2023.104279 38061225

[27] Nguemeni Tiako, M. J., Netherland, J., Hansen, H. & Jauffret-Roustide, M. Drug overdose epidemic colliding with COVID-19: what the United States can learn from France. *Am. J. Public Health* **112**, S128–S132 (2022). 10.2105/AJPH.2022.306763 PMC8965177 35349315

[28] Pol, S. et al. Impact of extending direct antiviral agents (DAA) availability in France: an observational cohort study (2015-2019) of data from French administrative healthcare databases (SNDS). *Lancet Reg. Health Eur.* **13**, 100281 (2021). 10.1016/j.lanepe.2021.100281 PMC8671622 34950924

[29] Midgard, H. et al. Hepatitis C treatment and reinfection surveillance among people who inject drugs in a low-threshold program in Oslo, Norway. *Int. J. Drug Policy* **96**, 103165 (2021). 10.1016/j.drugpo.2021.103165 33642182

[30] Cunningham, E. B. et al. Reinfection following successful direct-acting antiviral therapy for hepatitis C virus infection among people who inject drugs. *Clin. Infect. Dis.* **72**, 1392–1400 (2021). 10.1093/cid/ciaa253 32166305

[31] Akiyama, M. J. et al. Low hepatitis C reinfection following direct-acting Antiviral therapy among people who inject drugs on opioid agonist therapy. *Clin. Infect. Dis.* **70**, 2695–2702 (2020). 10.1093/cid/ciz693 PMC7456350 31346609

[32] Kleiner, B. B. et al. Incidence of HIV and HCV Among People Who Inject Drugs in Montreal, Canada: data From the HEPCO Longitudinal Cohort Study, 2011–2025. *J. Viral Hepatol.* **33**, e70185 (2026). 10.1111/jvh.70185 PMC13179476 42141790

[33] McDonald, S. A. et al. Estimating incidence rates of primary infection and reinfection with hepatitis C virus among people who inject drugs in Scotland: a model-based analysis of repeated cross-sectional survey data. *Lancet Reg. Health Eur.* **60**, 101505 (2026). 10.1016/j.lanepe.2025.101505 PMC12605865 41234782

[34] Degenhardt, L. et al. Epidemiology of injecting drug use, prevalence of injecting-related harm, and exposure to behavioural and environmental risks among people who inject drugs: a systematic review. *Lancet Glob. Health* **11**, e659–e672 (2023). 10.1016/S2214-109X(23)00057-8 PMC12580156 36996857

[35] Jauffret-Roustide, M., Pedrono, G. & Beltzer, N. Supervised consumption rooms: the French Paradox. *Int. J. Drug Policy* **24**, 628–630 (2013). 10.1016/j.drugpo.2013.05.008 23777651

[36] Lalanne, L. et al. Drug consumption rooms are effective to reduce at-risk practices associated with HIV/HCV infections among people who inject drugs: Results from the COSINUS cohort study. *Addict. Abingdon Engl.* **119**, 180–199 (2024). 10.1111/add.16320 37743675

[37] Litwin, A. H. et al. Hepatitis C virus reinfection among people who inject drugs: long-term follow-up of the HERO study. *JAMA Netw. Open* **7**, e2430024 (2024). 10.1001/jamanetworkopen.2024.30024 39186268

[38] Heffernan, A., Cooke, G. S., Nayagam, S., Thursz, M. & Hallett, T. B. Scaling up prevention and treatment towards the elimination of hepatitis C: a global mathematical model. * Lancet* **393**, 1319–1329 (2019). 10.1016/S0140-6736(18)32277-3 PMC6484702 30704789

[39] Gountas, I. et al. Treatment and primary prevention in people who inject drugs for chronic hepatitis C infection: is elimination possible in a high-prevalence setting? *Addict*. *Abingdon Engl.* **112**, 1290–1299 (2017). 10.1111/add.13764 PMC5553636 28107585

[40] Trickey, A. et al. The contribution of injection drug use to hepatitis C virus transmission globally, regionally, and at country level: a modelling study. *Lancet Gastroenterol. Hepatol.* **4**, 435–444 (2019). 10.1016/S2468-1253(19)30085-8 PMC6698583 30981685

[41] Buti, M., Domínguez-Hernández, R. & Casado, M. A. Impact of the COVID-19 pandemic on HCV elimination in Spain. *J. Hepatol.* **74**, 1246 (2020). 10.1016/j.jhep.2020.12.018 PMC7749992 33358780

[42] Hermetet, C. et al. Continuum of hepatitis C care in France: a 20-year cohort study. *PloS ONE* **12**, e0183232 (2017). 10.1371/journal.pone.0183232 PMC5574535 28850623

[43] Jauffret-Roustide, M. et al. Comparaison des profils, pratiques et situation vis-à-vis de l’hépatite C des usagers de drogues russophones et francophones à Paris, ANRS-Coquelicot Study, 2011-2013. *Bull. Epidémiol. Hebd*. **14-15**, 285–290 (2017).

[44] Makhoul, M. et al. Hepatitis C virus transmission among people who inject drugs in the Middle East and North Africa: mathematical modeling analyses of incidence and intervention impact. *eClinicalMedicine* **80**, 103040 (2025). 10.1016/j.eclinm.2024.103040 PMC11786755 39896871

[45] Hajarizadeh, B. et al. Hepatitis C reinfection after successful antiviral treatment among people who inject drugs: a meta-analysis. *J. Hepatol.* **72**, 643–657 (2020). 10.1016/j.jhep.2019.11.012 31785345

[46] Yeung, A. et al. Population-level estimates of hepatitis C reinfection post scale-up of direct-acting antivirals among people who inject drugs. *J. Hepatol.* **76**, 549–557 (2022). 10.1016/j.jhep.2021.09.038 PMC8852744 34634387

[47] Macías, J. et al. High prevalence of hepatitis C virus infection among noninjecting drug users: association with sharing the inhalation implements of crack. *Liver Int.* **28**, 781–786 (2008). 10.1111/j.1478-3231.2008.01688.x 18312289

[48] Leon, L., Jauffret-Roustide, M. & Le Strat, Y. Design-based inference in time-location sampling. *Biostat. Oxf. Engl.* **16**, 565–579 (2015). 10.1093/biostatistics/kxu061 25597489

[49] Maher, L. et al. Utilising Integrated Bio-behavioural Surveillance (IBBS) to investigate declining hepatitis C antibody prevalence among people who inject drugs in the Australian Needle and Syringe Program Survey. *Int. J. Drug Policy* **131**, 104545 (2024). 10.1016/j.drugpo.2024.104545 39159531

[50] Direction de la recherche, des études, de l’évaluation et des statistiques (DREES). La prise en charge des patients atteints d’hépatite C chronique. https://drees.solidarites-sante.gouv.fr/sites/default/files/2020-10/er470.pdf (2006).

[51] Kish, L. Cumulating/combining population surveys. *Surv. Methodol.* **25**, 129–138 (1999).

[52] Thomas, S. & Wannell, B. Combining cycles of the Canadian Community Health Survey. *Health Rep.* **20**, 53–58 (2009). 19388369

[53] Friedman, E. M. & Jang, D. Combined estimates from four quarterly survey data sets. Section on survey research methods. *Proc. Jt. Stat. Meet*. 1064–1969 (2002).

[54] Bénézet, L., Renuy, A., Delmas, M.-C. & Iwatsubo, Y. Estimations De Prévalences Par Combinaison Des Échantillons Annuels De La Cohorte Constances. in *11e Colloque International Francophone sur les Sondages* (Bruxelles, France, 2021).

[55] Delmas, M.-C. et al. Underdiagnosis of obstructive lung disease: findings from the French CONSTANCES cohort. *BMC Pulm. Med.* **21**, 319 (2021). 10.1186/s12890-021-01688-z PMC8518215 34649556

[56] Westat. Data on Health and Well-being of American Indians, Alaska Natives, and Other Native Americans. *ASPE* http://aspe.hhs.gov/reports/data-health-well-being-american-indians-alaska-natives-other-native-americans-0 (2006).

[57] Royston, P., Ambler, G. & Sauerbrei, W. The use of fractional polynomials to model continuous risk variables in epidemiology. *Int. J. Epidemiol.* **28**, 964–974 (1999). 10.1093/ije/28.5.964 10597998

[58] Binder, H., Sauerbrei, W. & Royston, P. Comparison between splines and fractional polynomials for multivariable model building with continuous covariates: a simulation study with continuous response. *Stat. Med.* **32**, 2262–2277 (2013). 10.1002/sim.5639 23034770

[59] Lefrère, J.-J. et al. Full or partial seroreversion in patients infected by hepatitis C virus. *J. Infect. Dis.* **175**, 316–322 (1997). 10.1093/infdis/175.2.316 9203652

[60] Le Page, A. K., Robertson, P. & Rawlinson, W. D. Discordant hepatitis C serological testing in Australia and the implications for organ transplant programs. *J. Clin. Virol.* **57**, 19–23 (2013). 10.1016/j.jcv.2012.12.019 23380661

[61] Kee, K.-M. et al. Decreased anti-hepatitis C virus titer and associated factors in chronic hepatitis C patients after sustained virological response: a prospective study. *J. Gastroenterol. Hepatol.* **27**, 1106–1111 (2012). 10.1111/j.1440-1746.2011.06946.x 22004331

[62] Thompson, W. W. Vital signs: hepatitis C treatment among insured adults—United States, 2019–2020. *MMWR Morb. Mortal. Wkly. Rep.* **71**, 1011–1017 (2022). 10.15585/mmwr.mm7132e1 PMC9400534 35951484

[63] WHO. Guidelines for the care and treatment of persons diagnosed with chronic hepatitis C virus infection. https://www.who.int/publications/i/item/9789241550345 (2018). 30307724

[64] Horvitz, D. G. & Thompson, D. J. A Generalization of sampling without replacement from a finite universe. *J. Am. Stat. Assoc.* **47**, 663–685 (1952).

[65] Sabbatini, C. E. *HCV Incidence and Prevalence Trends Among People Who Use Drugs in France 2004 to 2023*. 10.5281/zenodo.22054853 (2026). PMC13745531 43000429

[66] OFDT. Les niveaux d’usage des drogues illicites en France en 2023. https://www.ofdt.fr/publication/2024/les-niveaux-d-usage-des-drogues-illicites-en-france-en-2023-2122 (2024).

[67] Bulteel, N., Sarathy, P., Forrest, E., Stanley, A. & Innes, H. Factors associated with spontaneous clearance of chronic hepatitis C virus infection - ScienceDirect. *J. Hepatol.* **65**, 266–272 (2016). 10.1016/j.jhep.2016.04.030 27155531

[68] Smith, D. J., Jordan, A. E., Frank, M. & Hagan, H. Spontaneous viral clearance of hepatitis C virus (HCV) infection among people who inject drugs (PWID) and HIV-positive men who have sex with men (HIV+ MSM): a systematic review and meta-analysis. *BMC Infect. Dis.* **16**, 471 (2016). 10.1186/s12879-016-1807-5 PMC5011802 27595855

[69] Elbasha, E. H. Model for hepatitis C virus transmissions. *Math. Biosci. Eng. MBE* **10**, 1045–1065 (2013). 10.3934/mbe.2013.10.1045 23906202

[70] Mathers, B. M. et al. Mortality among people who inject drugs: a systematic review and meta-analysis. *Bull. World Health Organ.* **91**, 102–123 (2013). 10.2471/BLT.12.108282 PMC3605003 23554523

[71] Gjersing, L. & Bretteville-Jensen, A. L. Gender differences in mortality and risk factors in a 13-year cohort study of street-recruited injecting drug users. *BMC Public Health* **14**, 440 (2014). 10.1186/1471-2458-14-440 PMC4047552 24886464

[72] Meijerink, H. et al. Modelling the burden of hepatitis C infection among people who inject drugs in Norway, 1973-2030. *BMC Infect. Dis.* **17**, 541 (2017). 10.1186/s12879-017-2631-2 PMC5543437 28774261

[73] Hutchinson, S. J., Bird, S. M. & Goldberg, D. J. Modeling the current and future disease burden of hepatitis C among injection drug users in Scotland. *Hepatology* **42**, 711–723 (2005). 10.1002/hep.20836 16116637

[74] Sangiovanni, A. et al. The natural history of compensated cirrhosis due to hepatitis C virus: a 17-year cohort study of 214 patients. *Hepatology* **43**, 1303–1310 (2006). 10.1002/hep.21176 16729298

[75] Wang, H.-W., Wang, Y.-C., Huang, Y.-T. & Jia, M.-Y. All-cause and cause-specific mortality risk among men and women with hepatitis C virus infection. *Plos ONE* **19**,9, e0309819. https://journals.plos.org/plosone/article?id=10.1371/journal.pone.0309819 10.1371/journal.pone.0309819 PMC11383219 39250481

