# Supplementary material for: HCV incidence and prevalence trends among people who use drugs in France 2004 to 2023
Source: Nat Commun. 2026 Oct 11;17:11525. doi: 10.1038/s41467-026-78598-3 (PMC13745531; doi:10.1038/s41467-026-78598-3)
Supplement: Supplementary file 1 [file 41467_2026_78598_MOESM1_ESM.pdf]

SUPPLEMENTARY INFORMATION

HCV incidence and prevalence trends among people who use drugs in France 2004 to 2023

Chiara E. Sabbatini[1], Yann Le Strat[1], Christophe Bonaldi[1], Cécile Brouard[1], Martin Audran[2], Leïla Saboni[1], Jean-Baptiste Richard[1], Amélie David[3], Catherine Gaudy-Graffin[3], Stephane Chevaliez[4], Francis Barin[3], Karl Stefic[3], Marie Jauffret-Roustide[2,5,6]

[1] Santé publique France, French National Public Health Agency, Saint-Maurice, France.
[2] Centre d'étude des mouvements sociaux (Inserm U1276/CNRS UMR 8044/EHESS), Paris, France
[3] Université de Tours, Inserm U1259 MAVIVH, and CHU de Tours, Laboratoire associé au CNR du VIH, Tours, France. [4]Department of Virology, French National Reference Centre for hepatitis B, C and delta viruses, Hôpital Henri Mondor (AP-HP), Université Paris-Est, Créteil, France
[5] Centre hospitalier Le Vinatier, Lyon, France.
[6] Institut Universitaire sur les Dépendances, Montréal, Québec, Canada.
** Corresponding author. *chiara.sabbatini@santepubliquefrance.fr*

1. ANRS-COQUELICOT SURVEYS 2
- 1.1 Inclusion criteria 2
- 1.2 Sampling design and sampling weights 2
- 1.3 Molecular and serological data 2
- 1.4. Breakdown of the PWUD population by molecular and serological status 3
- 1.5. Recruitment distribution across services 3
- 1.6. HCV antibody positive data correction 4

2. DESCRIPTIVE STATISTICS 5

3. MULTIVARIATE REGRESSION MODELS 6

4. TRANSMISSION MODELS 7
- 4.1. Compartmental models and parameters 7
- 4.2. Confidence intervals 10
- 4.3. Global incidence 10

5. ADDITIONAL RESULTS 11
- 5.1 Prevalence across generations 11
- 5.2 Prevalence and incidence in PWID 12
- 5.3 Prevalence and incidence in minorities 13

6. SENSITIVITY ANALYSIS 14
- 6.1 Impact of spontaneous clearance in the acute phase 14
- 6.2 Impact of the percentage of population on DAA treatments 15

7. List of Coquelicot partner structures acknowledged 16

REFERENCES 19

1. ANRS-COQUELICOT SURVEYS

1.1 Inclusion criteria

While the core inclusion criteria of the Coquelicot surveys have remained largely stable[1], some adjustments have been made over time in response to changes in drug use practices and the characteristics of the target population. For instance, in the first survey in 2004, participants were required to have a history of either injecting or snorting drugs in their lifetime. However, in subsequent editions (2013 and 2023), the criteria were expanded to include individuals who had smoked crack cocaine, reflecting both the increasing prevalence of crack use and growing evidence on its potential role in HCV transmission[1].

Regarding language, in 2004, the questionnaire was available only in French, as fewer than 5% of people attending services reported not speaking French. However, by 2013, up to 20% of PWUD in some Paris sites spoke Russian, leading to the addition of a Russian version of the questionnaire. In 2023, linguistic diversity had increased further, requiring additional translations to reach a broader non-French-speaking population. These were not changes in inclusion criteria per se, but rather adaptations to ensure adequate coverage of an evolving population.

1.2 Sampling design and sampling weights

To generate population-level estimates among people who use drugs (PWUD), all analyses accounted for the sampling designs of the three surveys, including sampling weights, stratification, and primary sampling units.

We implemented a two-stage Time-Location Sampling (TLS) design to obtain a representative sample of PWUD attending participating services[2]. In the first stage, we did an exhaustive inventory of all services dedicated to PWUDs. For each participating center, we constructed a comprehensive sampling frame including all opening half-days during the survey period. From this frame, a predefined number of half-day time slots were randomly selected. In the second stage, during each selected time slot, we applied systematic random sampling to invite individuals attending the service to participate. Sampling weights were constructed to reflect both stages of the design. Participants' frequency of attendance at service centers was incorporated into the construction of the sampling weights. The sampling weight of an individual results from multiplying the time-slot service weight (the inverse of the inclusion probability) by the individual weight (the inverse of the probability of selection during the visit). The weighting process also accounts for self-reported service visits in the days before the interview by dividing by the number of services frequented, as done in Ref.[2]. Finally, the process applies a finite population correction (fpc) at the regional level, based on the proportion of time slots actually surveyed with respect to the total available during the study period.

1.3 Molecular and serological data

Dried blood spots (DBS) were collected from participants on a voluntary basis. Six drops of capillary blood were spotted on a filter paper card. To ensure sample quality, we required each drop to have a minimum diameter of 6 mm. Cards were dried at room temperature and then kept in individual zipped plastic bags to be sent daily to a National Reference Center (NRC).

Serological and molecular analyses were carried out as previously described in Refs.[3–5] . Serological analyses were carried out by the HIV National Reference Center (NRC) - University Hospital of Tours, Tours, France. DBS were eluted in 0.01 M sodium phosphate buffer containing 10% bovine serum albumin and 0.05% Tween 20 (PBS-BSA-TW), then incubated overnight at +4°C. Anti-HIV and anti-HCV were tested using ELISA tests (Genscreen ULTRA HIV Ag/Ab catalog number 72386, Monolisa Anti-HCV PLUS V3 catalog number 72340 and Monolisa HBs Ag ULTRA, Biorad, Marnes-la-Coquette, France). To classify individuals as HCV seropositive or seronegative, we analyzed the distribution of quantitative anti-HCV antibody test results. The traditional biological approach, relies on a specific

detection threshold to determine the presence or absence of anti-HCV antibodies. Of note, DBS has a known lower sensitivity compared to serum, requiring to lower the signal-to-cutoff (s/co) ratio below 1.0 (i.e. the s/co ratio for serum). An s/co ratio of 0.29 was used as anti-HCV positivity cut-off, which yielded the highest classification agreement with reported HCV infection status. The analytical specificity of the assay using this ratio was further confirmed on a panel of 214 anti-HCV negative samples, leading to a specificity of 99.9%. This method provides a binary result: the individual is classified as seropositive or seronegative. For 2004 and 2013, biological values were modeled using mixture models of normal distributions, as described in Ref[6]. This approach led to a high level of classification agreement with standard binary results, with a discrepancy of approximately 1.5% between methods. Thus, classification was performed using only the binary outcome in 2023.

Molecular analyses were carried out by the Viral Hepatitis NRC (University Hospital Henri-Mondor, Créteil, France) for the 2013 survey and by the HIV NRC for the 2023 one. HCV RNA was quantified using the Cobas Ampliprep/Cobas TaqMan HCV version 2 (CAP/CTM HCV v2.0, Roche Molecular Systems, Pleasanton, California, USA) or the Abbott RealTime HCV Amplification Reagent Kit (Abbott Molecular, Des Plaines, Illinois), respectively, according to the manufacturer's recommendations. For Cobas, DBS was eluted in 1.1 mL of lysis buffer (Cobas AmpliPrep/Cobas TaqMan Specimen Pre-extraction (SPEX) at 56°C with gentle agitation for 30 min and centrifuged at 36 220*g.* A total of 500 µL of pre-extraction supernatant was added to perform the CAP/CTM HCV v2.0 assay. For Abbott, DBS were eluted in 1.3 mL of mDBS buffer (mSample Preparation system DBS Buffer Kit) at ambient temperature for 30 min.

1.4. Breakdown of the PWUD population by molecular and serological status

Table S1. Breakdown of the PWUD population by HCV RNA and HCV antibody test results: 2013 and 2023 datasets.

2013	HCV RNA -	HCV RNA +	Total
Anti-HCV-	753	13	766
Anti-HCV+	281	260	541
Total	1034	273	1307

2023	HCV RNA -	HCV RNA +	Total
Anti-HCV-	709	7	716
Anti-HCV+	219	87	306
Total	928	94	1022

1.5. Recruitment distribution across services

The data reveal a highly skewed distribution of recruitment of PWUD across services. As shown in **Figure S1a-c** most services contribute modestly to recruitment, typically enrolling fewer than 20 participants. However, a small number of services accounts for a large share of the total. This pattern remains consistent across all survey editions (2004, 2013, and 2023). The boxplots in **Figure S1d-f** further illustrate this disparity, particularly in cities like Paris, where certain services recruit between 50 and 140 participants. This heterogeneity likely reflects structural factors, such as service capacity, population density, or outreach strategies, with some centers serving as key recruitment hubs. Such variability is an inherent feature of harm reduction and addiction care.

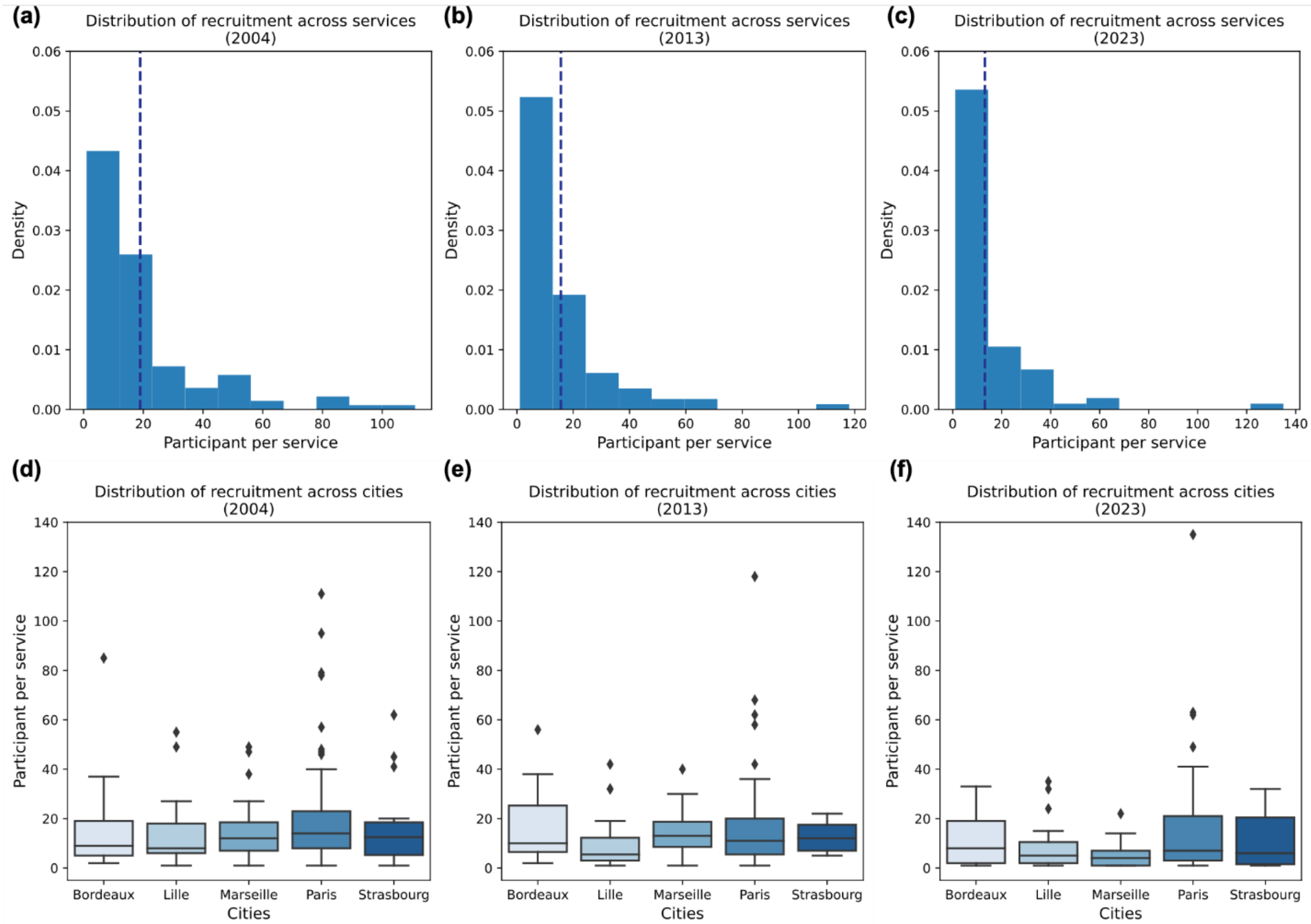

Figure S1. Distribution of recruitment across services and cities. (a-c) Histograms showing the distribution of recruitments among the services included in the study for 2004 **(a)**, 2013 **(b)** and 2023 **(c)**. The blue dashed line represents the mean value. **(d-f)** Boxplots showing the number of participants per service in Bordeaux, Lille, Marseille, Paris, and Strasbourg for 2004 **(d)**, 2013 **(e)** and 2023 **(f)**. Box plots represent the median (line in the middle of the box), interquartile range (box limits) and 2.5th and 97.5th percentiles (whiskers) and outliers for each city, illustrating the variability in recruitment. Total sample sizes per survey year were n = 573 (2004), n = 1,307 (2013), and n = 1,022 (2023). Paris exhibits the highest variability, with some services recruiting significantly more participants than others.

1.6. HCV ANTIBODY POSITIVE DATA CORRECTION

Following what was done in Ref.[7] we redefined the prevalence of antiHCV+ for 2013 and 2023 as those with either a positive antiHCV+ test result or a self-reported history of ever having HCV treatment. **Figure S2** compares original and corrected prevalence estimates. The correction slightly attenuated the decline in antibody prevalence, resulting in an overall median increase of 7.7% in 2013 and 15.2% in 2023 compared to the original data.

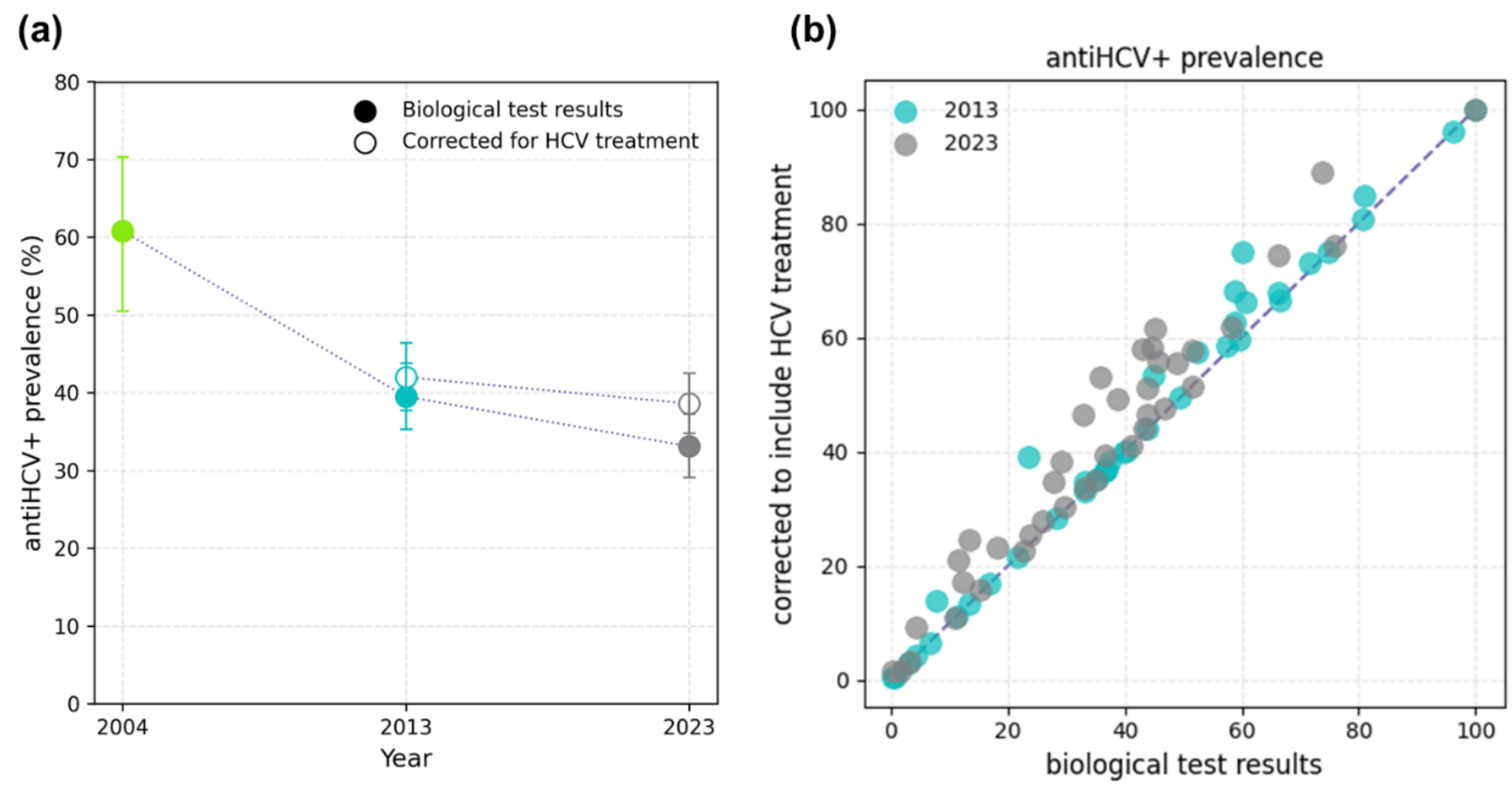

Figure S2. **Comparison of anti-HCV+ prevalence data: biological test results vs. corrected for HCV treatment. (a)** AntiHCV+ prevalence data for 2004, 2013 and 2023. Filled circles represent biological test results. Void circles represent corrected data to include HCV treatment. The mean values are displayed; error bars represent 95%CI accounting for the survey's weighting design, based on samples of n = 573 (2004), n = 1307 (2013), and n = 1022 (2023) respondents. **(b)** Scattered plot between corrected antiHCV+ prevalence data and antiHCV+ prevalence data from biological test results for different ages. Data are shown for 2013 (light blue) or 2023 (grey). Results for these indicators refer to the national scale.

2. DESCRIPTIVE STATISTICS

The Coquelicot surveys (2004, 2013, 2023) enrolled 1462, 1718 and 2125 respondents. Available and valid test results ranged from 817, 1468 and 1699, yielding final analytical samples of 573 (2004), 1307 (2013), and 1022 (2023) as shown in **Figure S3**. **Table S2** details the statistics of retained samples.

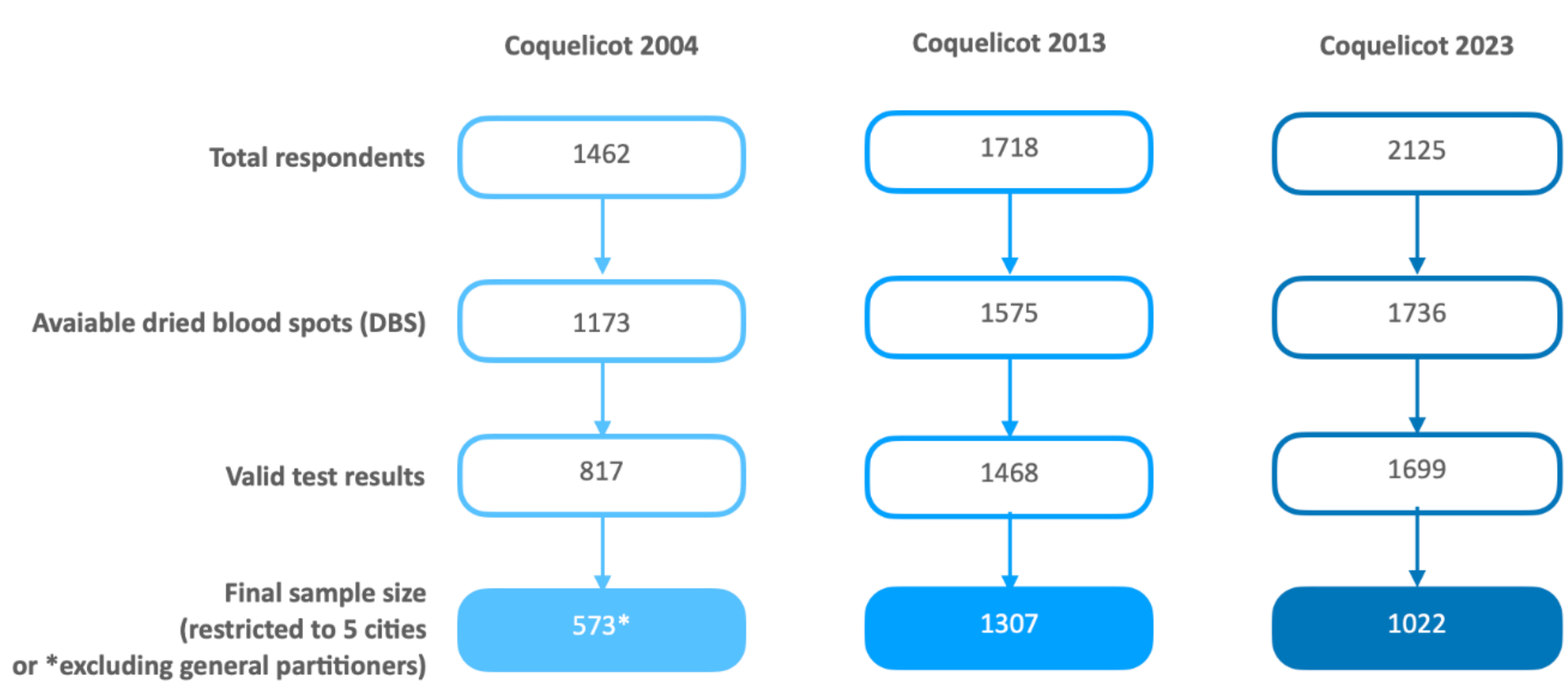

Figure S3. Flowchart of participant inclusion and DBS testing across the Coquelicot surveys (2004, 2013, and 2023). The figure shows the number of total respondents, available DBS samples, valid test results, and final sample sizes (restricted to 5 cities or excluding general partitioners for 2004) for each survey year.

Table S2. Descriptive statistics of PWUD of ANRS-Coquelicot in 2004, 2013 and 2023. Estimates account for survey design. The table shows estimated percentages, 95% confidence intervals and unweighted observations (n). P-values were derived from second-order Rao-Scott chi-square test.

	2004		2013		2023		p-value[1]
	% [95% CI]	n	% [95% CI]	n	% [95% CI]	n	
Total participants	-	573	-	1307	-	1022	
Gender							0.01
Men	71.6% [61.0%, 80.2%]	438	80.9% [77.5%, 84.0%]	1046	84.6% [81.3%, 87.5%]	874	
Women	28.4% [19.8%, 39.0%]	135	19.1% [16.0%, 22.5%]	258	15.4% [12.5%, 18.7%]	148	
Age classes							<0.001
1: 18-20	0.5% [0.2%, 1.5%]	6	0.7% [0.3%, 1.6%]	11	0.3% [0.1%, 0.7%]	6	
2: 21-25	6.7% [4.1%, 10.8%]	69	8.3% [6.4%, 10.6%]	132	5.4% [4.0%, 7.3%]	61	
3: 26-35	48.7% [38.4%, 59.1%]	251	28.5% [24.7%, 32.7%]	387	15.4% [12.7%, 18.6%]	170	
4: 36-45	40.0% [31.4%, 49.2%]	218	42.0% [37.7%, 46.4%]	531	33.3% [29.5%, 37.3%]	348	
5: 46-55	3.7% [2.1%, 6.6%]	28	18.2% [15.1%, 21.8%]	215	32.4% [28.5%, 36.5%]	331	
6: >56	0.4% [0.1%, 2.4%]	1	2.4% [1.5%, 3.8%]	31	13.2% [10.4%, 16.7%]	106	
HCV RNA+	-	-	19.4% [16.3%, 23.0%]	273	9.5% [7.2%, 12.3%]	94	<0.001
Anti-HIV+	10.8% [6.5%, 17.6%]	58	9.4% [7.2%, 12.2%]	106	4.6% [3.1%, 6.9%]	36	0.021
Anti-HCV+	60.8% [50.5%, 70.3%]	305	39.5% [35.3%, 43.8%]	541	33.1% [29.1%, 37.3%]	306	<0.001
Anti-HCV+[2]	-	-	42.0% [37.7%, 46.4%]	570	38.6% [34.8%, 42.5%]	381	0.002
PWID	70.1% [58.3%, 79.7%]	415	63.4% [59.1%, 67.5%]	856	61.7% [57.6%, 65.6%]	619	0.3
PWAID	27.1% [17.6%, 39.3%]	151	25.1% [21.6%, 29.0%]	348	31.6% [27.7%, 35.8%]	331	0.2
Cocaine crack	38.6% [26.1%, 52.9%]	100	34.6% [30.5%, 38.9%]	350	57.7% [53.4%, 61.9%]	623	<0.001

[1]second-order Rao-Scott chi-square test, [2]Corrected accounting for HCV treatment

3. MULTIVARIATE REGRESSION MODELS

We tested several models incorporating both age and survey edition as covariates, including alternative models that used splines instead of fractional polynomials to model age, and models with interactions between age and survey edition. These models are reported in Table S3 are not identified as best fitting models via AIC criterion.

Table S3. Logistic regression models performed to estimate anti-HCV prevalence in drug user populations. P-values were derived from two-sided t-tests accounting for sampling weights, stratification, and clustering. SE stands for standard error.

Variable	Fractional polynomial transformation ($\boldsymbol{\eta(a)}$) or splines base	Regression coefficient (SE)	p-value
Anti-HCV			
**PWUD, splines**			
Age			< 2e-06
	bs(age, df = 3)1	5.7 (1.9)	
	bs(age, df = 3)2	5.6 (2.2)	
	bs(age, df = 3)3	1.7 (4.2)	
Edition 2013 (ref 2004)		-1.2 (0.2)	1.3e-06
Edition 2023 (ref 2004)		-1.8 (0.3)	2.9e-12
Intercept		-2.5 (0.6)	2.0e-05
**PWUD, interaction**			
Age			0.00230
	age_1: $\left(a/_{10}\right)^{0.5}$	44.2 (14.5)	
	age_2: $\left(a/_{10}\right)^{1}$	-10.8 (3.9)	
Edition 2013 (ref 2004)		8.5 (15.4)	0.5798
Edition 2023 (ref 2004)		19.7 (14.6)	0.1779
age_1:edit2013		-12.0 (16.3)	0.4628
age_2:edit2013		3.6 (4.3)	0.4016
age_1:edit2023		-24.2 (15.6)	0.1203
age_2:edit2023		6.7 (4.1)	0.1012
Intercept		-43.9 (13.4)	0.00103

4. TRANSMISSION MODELS

4.1. COMPARTMENTAL MODELS AND PARAMETERS

We used two distinct two-state compartmental models: one to model seroconversion and seroreversion, and another to model the dynamics of the two HCV RNA states (+/-). Both models are illustrated in **Figure S4** and parameters are reported in **Table 2.**

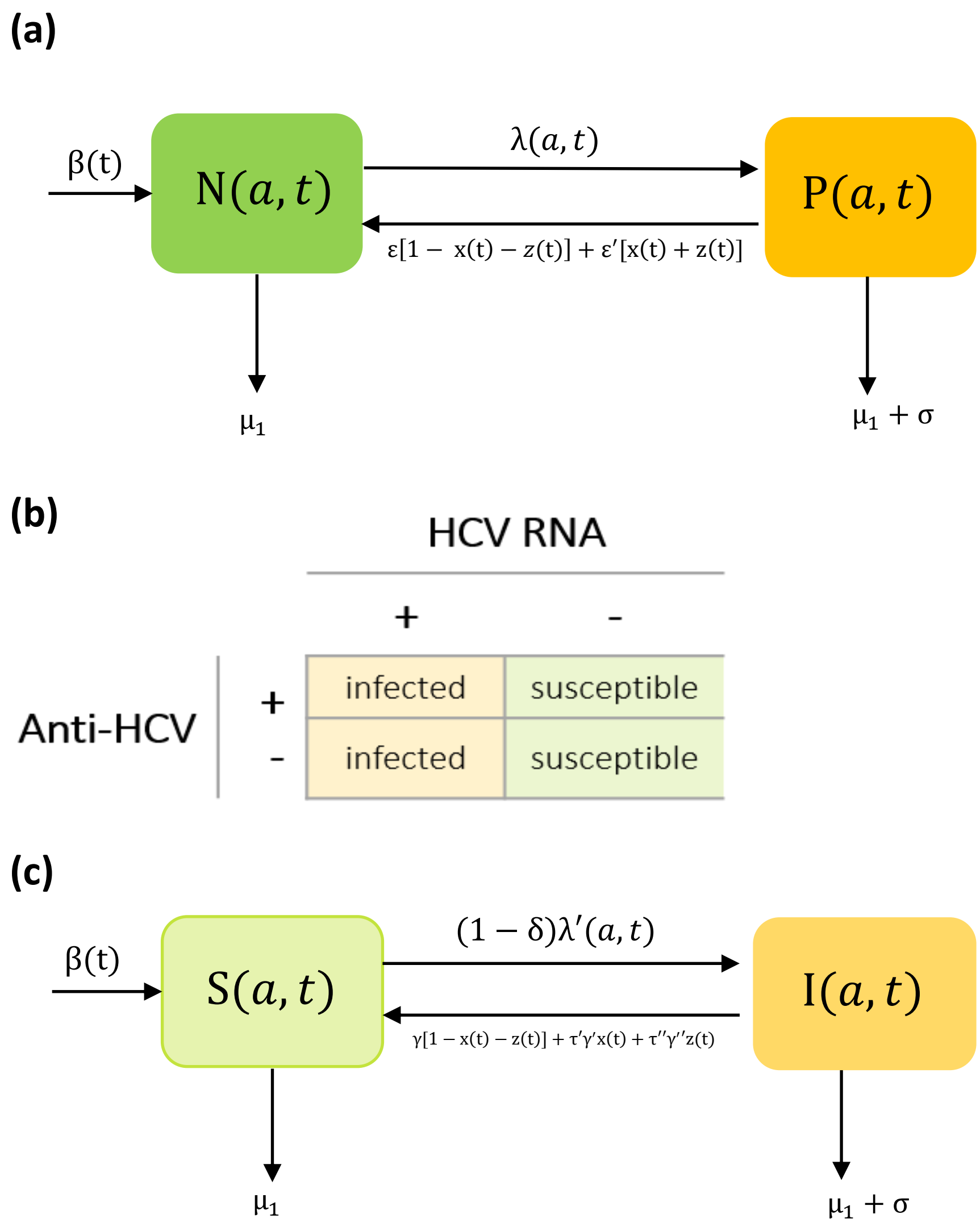

Figure S4. Compartmental models for HCV transmission. (a) Seroconversion and seroreversion model. N(a,t) represents anti-HCV- PWUD (or PWID) of age a at survey time t (2004, 2013, or 2023), while P(a,t) represents antiHCV+ individuals of age a at the same time points. Model parameters are detailed in **Table 2**. **(b)** Mapping between antibody and RNA classifications. The table illustrates the correspondence between antibody status (seronegative or seropositive) and HCV RNA status (susceptible or infected). **(c)** Model for susceptible and infected PWUD (or PWID). S(a,t) represents susceptible PWUD (or PWID) of age a at survey time t (2013 or 2023), and I(a,t) represents infected individuals of the same age and time points. Model parameters are detailed in **Table 2**.

The HCV transmission model presented in **Figure S4a** is then expressed in the form of partial differential equations by the following system:

$$\begin{cases} \frac{\partial N(a,t)}{\partial a} + \frac{\partial N(a,t)}{\partial t} = -\lambda(a,t)N(a,t) - \mu_1 N(a,t) + \beta(t)N(a,t) + \epsilon[1 - x(t) - z(t)]P(a,t) + \epsilon'[x(t) + z(t)]P(a,t) \\ \frac{\partial P(a,t)}{\partial a} + \frac{\partial P(a,t)}{\partial t} = \lambda(a,t)N(a,t) - (\mu_1 + \sigma)P(a,t) - \epsilon[1 - x(t) - z(t)]P(a,t) - \epsilon'[x(t) + z(t)]P(a,t) \end{cases} \quad (1)$$

The model is formulated in terms of normalized proportions among the population, such that: $N(a,t) + P(a,t) = 1$, as previously done in literature[8–12]. We can then substitute $N(a,t) = 1 - P(a,t)$ in the first equation of (3), and express the incidence of HCV infection, $\lambda(a,t)$, in terms of the derivative of the prevalence function:

$$\frac{\partial(1-P(a,t))}{\partial a} + \frac{\partial(1-P(a,t))}{\partial t} = -\lambda(a,t)(1-P(a,t)) - \mu_1(1-P(a,t)) + \beta(t)(1-P(a,t)) + \epsilon[1-x(t)-z(t)]P(a,t) + \epsilon'[x(t)+z(t)]P(a,t)$$

Then:

$$\lambda(a,t)(1-P(a,t)) = \frac{\partial P(a,t)}{\partial a} + \frac{\partial P(a,t)}{\partial t} - \mu_1(1-P(a,t)) + \beta(t)(1-P(a,t)) + \epsilon[1-x(t)-z(t)]P(a,t) + \epsilon'[x(t)+z(t)]P(a,t)$$

So:

$$\lambda(a,t)(1-P(a,t)) = \frac{\partial P(a,t)}{\partial a} + \frac{\partial P(a,t)}{\partial t} - \mu_1(1-P(a,t)) + \beta(t)(1-P(a,t)) + \epsilon[1-x(t)-z(t)]P(a,t) + \epsilon'[x(t)+z(t)]P(a,t)$$

where $P(a,t) = P(Y=1|a,t)$.

By performing the calculations, we obtain the expression for the incidence as a function of age *a* at a given fixed time *t*.

$$\lambda(a|t) = \frac{\frac{\partial P(a|t)}{\partial a} + (\beta - \mu_1) - \left(\beta - \mu_1 - \epsilon\big(1 - x(t) - z(t)\big) - \epsilon' x(t) - \epsilon' z(t)\right)P(a|t)}{1 - P(a|t)}$$

Thus, with the logit link, the derivative of the prevalence by age *a*, at a time t, can be deduced from equation (1) in the **Method** section of the main paper:

$$\lambda(a|t) = \frac{\eta'(a)P(a|t)\big(1-P(a|t)\big) + (\beta - \mu_1) - \left(\beta - \mu_1 - \epsilon\big(1 - x(t) - z(t)\big) - \epsilon' x(t) - \epsilon' z(t)\right)P(a|t)}{1 - P(a|t)}$$

Regarding the HCV RNA model, we can imagine a compartmental structure including one susceptible compartment (*S*) and two infectious compartments corresponding to acute (I_{ac}) and chronic (I_{ch}) infection stages (**Figure S5**). In this framework, individuals in the acute infection compartment could spontaneously clear the virus and return to the susceptible state, progress to chronic infection, or exit because of death. Individuals in the chronic infection compartment could either exit because of death or return to the susceptible state following spontaneous clearance during the chronic phase or sustained virologic response (SVR) after treatment. However, because our data do not allow us to distinguish between acute and chronic infections and only provide information on RNA positivity versus negativity, we adopted a simplified model with a single infectious compartment (**Figure S4c**). This compartment incorporates all possible pathways through which individuals may return to the susceptible state, including spontaneous clearance and treatment-induced SVR.

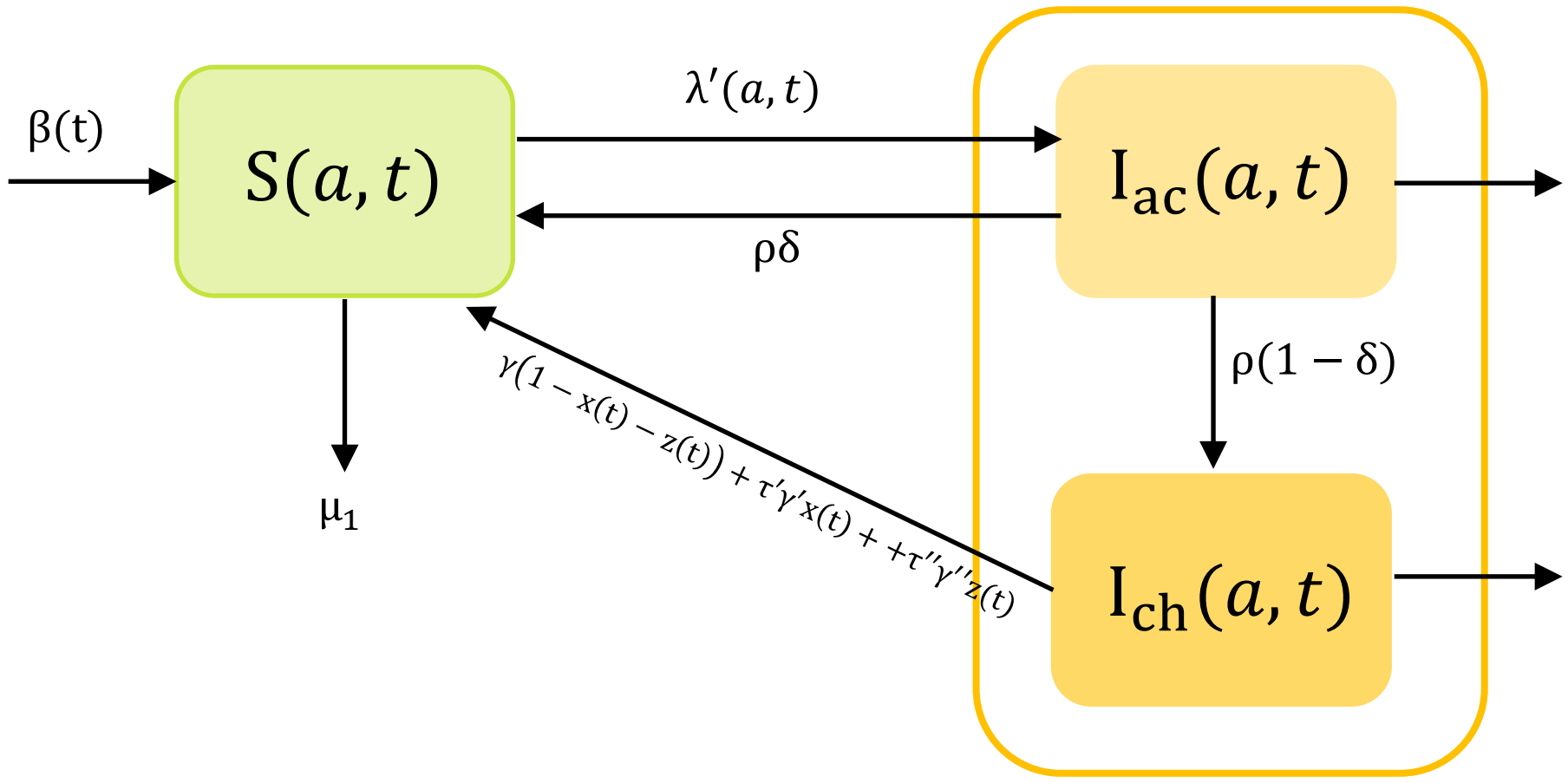

Figure S5. Compartmental models for HCV transmission. Complete model for susceptible and infected PWUD (PWID). S(a,t) represents susceptible individuals of age a at edition time t (2013 or 2023). Infected individuals are divided into acute (I_{ac} (a,t)) and chronic (I_{ch} (a,t)) categories, where I_{ac} (a,t) represents acutely infected individuals of age a at time t, and I_{ch} (a,t) represents chronically infected individuals of the same age and time points. Model parameters are described in **Table 2.** ρ is the mean duration time in the acute phase.

The equations governing the compartmental model presented in **Figure S4c** are as follows:

$$\begin{cases} \dfrac{\partial S(a,t)}{\partial a} + \dfrac{\partial S(a,t)}{\partial t} = -(1-\delta)\lambda'(a,t)S(a,t) - \mu_1 S(a,t) + \beta(t) + [\gamma[1 - x(t) - z(t)] + \tau'\gamma' x(t) + \tau''\gamma'' z(t)]I(a,t) \\ \dfrac{\partial I(a,t)}{\partial a} + \dfrac{\partial I(a,t)}{\partial t} = (1-\delta)\lambda'(a,t)S(a,t) - (\mu_1 + \sigma)I(a,t) - [\gamma[1 - x(t) - z(t)] + \tau'\gamma' x(t) + \tau''\gamma'' z(t)]I(a,t) \end{cases}$$

Substituting $S(a,t) = 1 - I(a,t)$ in the first equation we obtain for a given t:

$$\lambda'(a|t) = \frac{\dfrac{\partial I(a|t)}{\partial a} + (\beta - \mu_1) - (\beta - \mu_1 - [\gamma[1 - x(t) - z(t)] + \tau'\gamma' x(t) + \tau''\gamma'' z(t)])I(a|t)}{(1 - I(a|t))(1-\delta)}$$

As a consequence of our modeling assumptions, the inferred incidence (λ′(a,t)) includes both primary infections and reinfections, with the latter occurring among individuals who cleared the infection following sustained virologic response (SVR) or successful treatment.

4.2. Confidence intervals

We used a bootstrap approach to assess the variances of the estimates, generating 2000 resampled datasets combining data from the three surveys. First, individuals were replicated proportionally to their survey weights, creating an expanded pseudo-population. For each bootstrap iteration, we assigned a random number to each record (including the copies generated by duplication). Records were then reordered by survey year and by these random numbers. We then selected the first *n* observations for each survey-year-specific ordered list, with sample sizes of *n=573* for 2004, *n=1307* for 2013, and *n=1022* for 2023. This procedure was repeated 2000 times. For each resample dataset, we calculated prevalence while accounting for the survey design. To estimate incidence, we randomly drew the parameters following the approach of Ref.[4]. Finally, we determined the 95% confidence intervals by extracting the 2.5th and 97.5th percentiles from the distribution of estimated prevalences and incidences across the 2000 generated samples.

4.3. Global incidence

We estimated a global incidence in all PWUD and PWID. Values are reported in **Table S4.**

Table S4. Estimation of HCV incidence (per 100 person-years) in French drug users, in 2004, 2013 and 2023.

	2004	2013	2023
Anti-HCV			
PWUD	9.6 [7.5 – 11.7]	4.0 [2.5 – 5.5]	2.6 [1.2 – 4.0]
PWID	14.6 [11.5 – 18.9]	7.4 [5.4 – 9.4]	4.0 [2.3 – 5.8]
HCV RNA			
PWUD	-	4.1 [2.6 – 6.1]	1.7 [0.8 – 2.7]
PWID	-	6.5 [4.1 – 8.0]	2.4 [1.4 – 4.2]

5. ADDITIONAL RESULTS

5.1 Prevalence across generations

Regardless of age, antibody prevalence decreases between 2004, 2013, and 2023. The observed decrease likely reflects both the aging and mortality of individuals with HCV antibodies, and a lower rate of new infections among recent PWUD. Younger users are also less exposed to older ones—who have historically acted as reservoirs of transmission. Many young people began injecting at a time when access to sterile equipment and harm reduction services was more widespread[1]. Anti-HCV prevalence is higher in the older birth cohorts (**Figure S6**). This trend could indicate that older birth cohorts were more likely to be exposed to HCV (cohort effect).

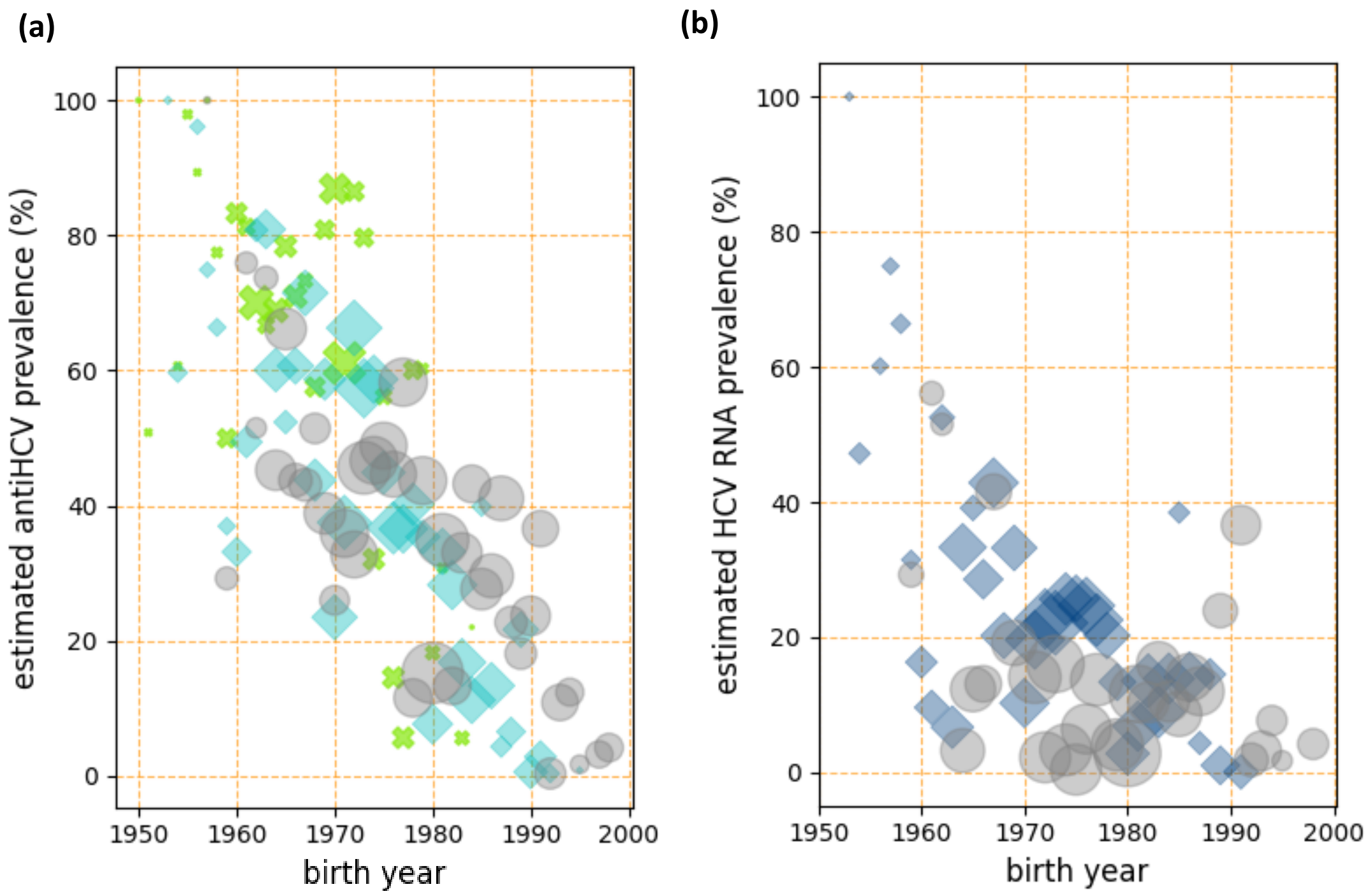

Figure S6. Prevalence of anti-HCV and HCV RNA. (a) Anti-HCV prevalence by generation, estimated for PWUD in 2004 (green), 2013 (light blue), and 2023 (grey). Symbols indicate age-specific prevalence estimates: crosses for 2004, diamonds for 2013, and circles for 2023. Symbol size is proportional to survey weight. **(b)** HCV RNA prevalence by generation, estimated for PWUD in 2013 (blue) and 2023 (grey). Symbols indicate age-specific prevalence estimates: diamonds for 2013 and circles for 2023. Symbol size is proportional to survey weight.

5.2 Prevalence and incidence in PWID

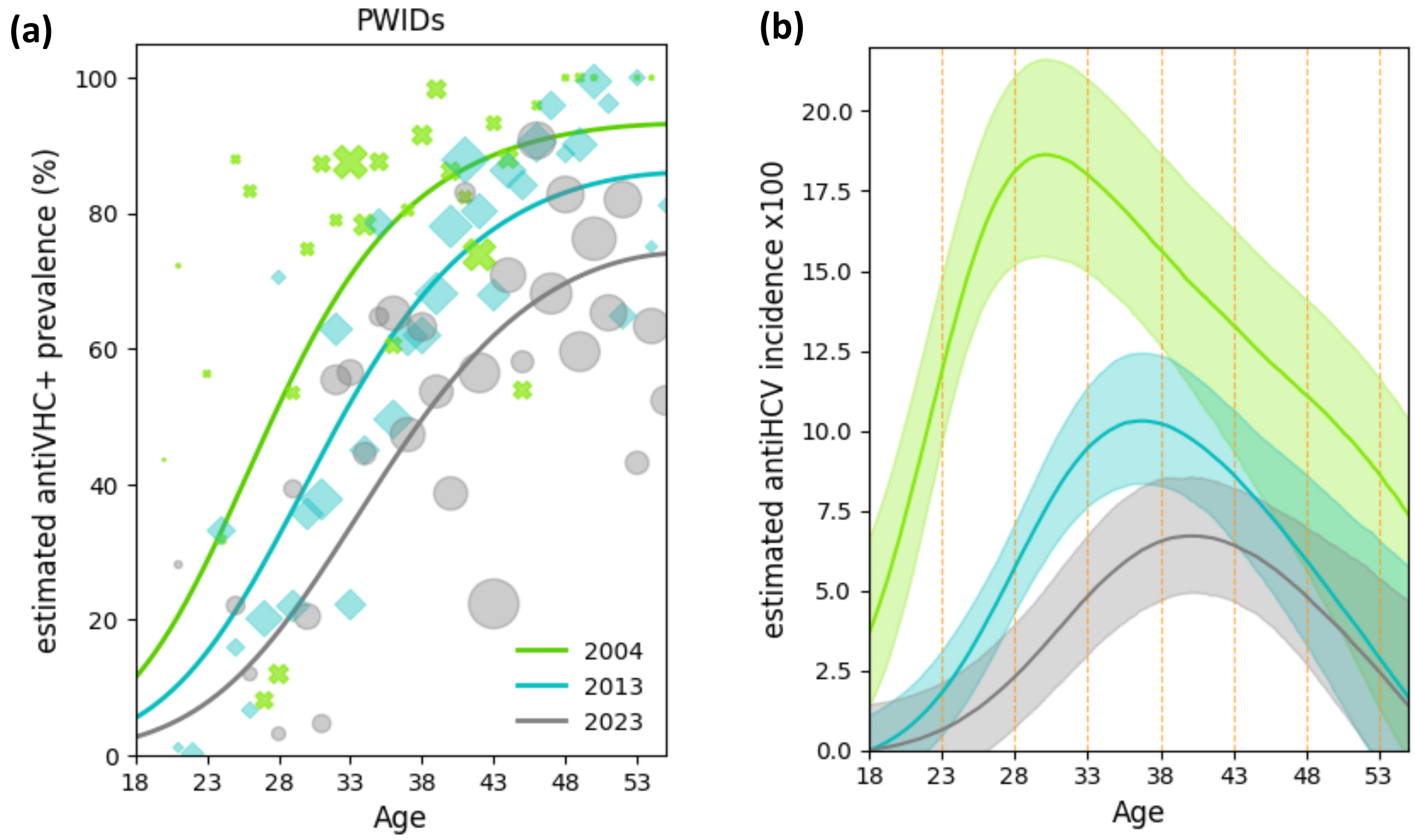

Figure S7. Anti-HCV prevalence and incidence in PWID (a) Anti-HCV prevalence by age, estimated from the regression model applied to PWIDs in 2004 (green), 2013 (light blue) and 2023 (grey). Confidence intervals are not shown for readability. Symbols represent age-specific prevalence estimates: crosses for 2004, diamonds for 2013 and circles for 2023. The size of the symbol is proportional to the survey weight. (b) AntiHCV incidence by age, in PWIDs in 2004 (green), 2013 (blue) and 2023 (black).

5.3 Prevalence and incidence in minorities

Here, we present prevalence and incidence results for specific subpopulations of PWUD, including individuals without stable housing, those with a history of incarceration, and crack-cocaine users. In 2023, prevalence and incidence appear slightly higher across all these subgroups. There is also the critical issue of access to DAA for people without social health coverage, as highlighted by Ref.[13].

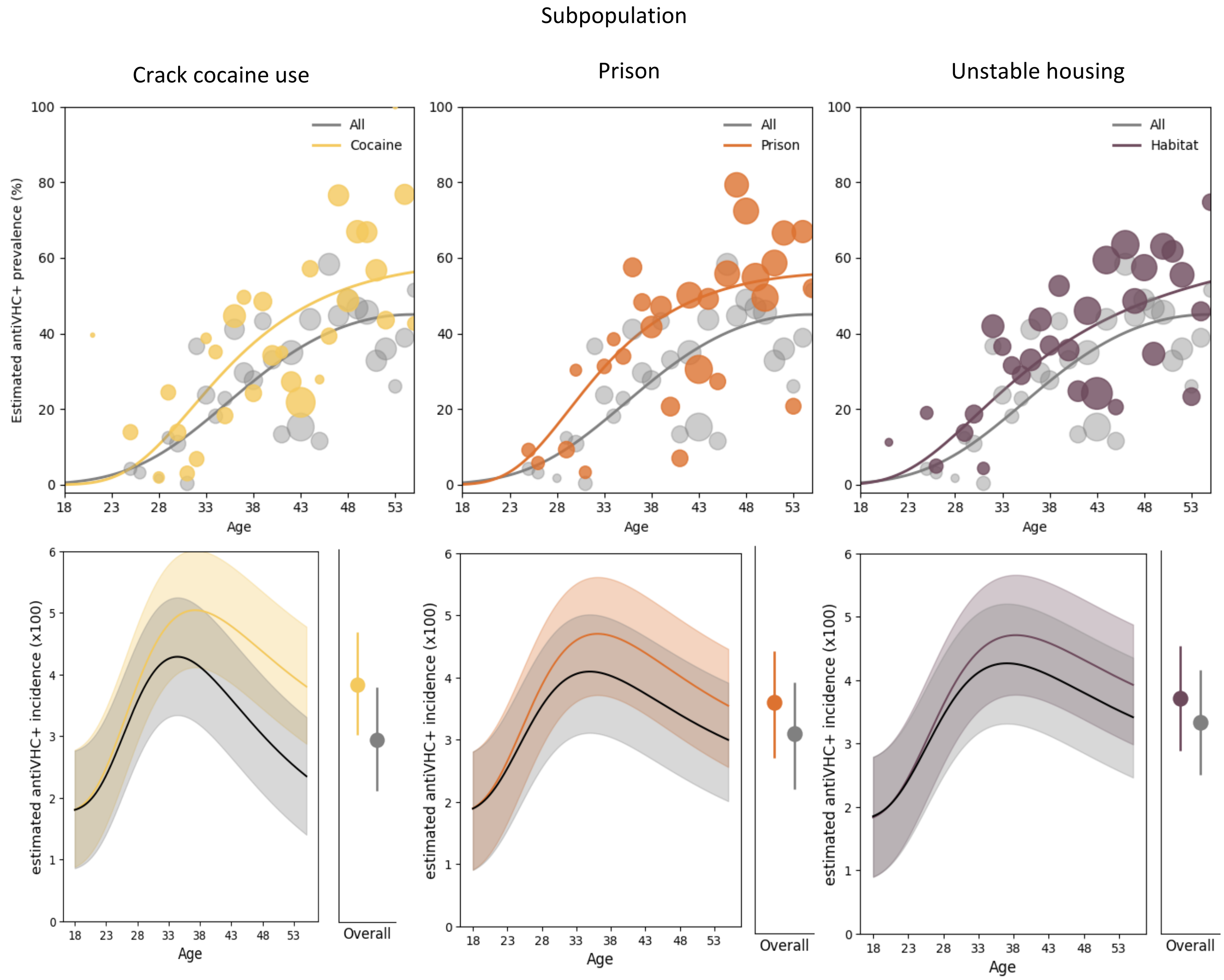

Figure S8. Impact of being in a specific subpopulation on the prevalence and incidence of anti-HCV in 2023. (Top) anti-HCV prevalence as a function of age, estimated for PWUD using crack cocaine (**left**, yellow), individuals with a history of incarceration (**center**, orange), and people without stable housing (**right**, purple). The grey line and points represent the reference scenario for all PWUD not using crack cocaine (**left**), individuals without a history of incarceration (**center**), and people with stable housing (**right**). Confidence intervals are not shown for readability. Symbols indicate age-specific prevalence estimates for 2023, with symbol size proportional to survey weight. **(Bottom)** Anti-HCV incidence as a function of age and overall incidence, estimated for PWUD using crack cocaine (**left**, yellow), individuals with a history of incarceration (**center**, orange), and people without stable housing (**right**, purple). Shaded areas indicate 95% confidence intervals (CIs). The mean values are displayed, vertical lines represent 95% confidence intervals, based on n = 573 (2004), n = 1307 (2013), and n = 1022 (2023) participants and N=2000 bootstrap samples.

6. SENSITIVITY ANALYSIS

Here we present the results of our sensitivity analysis on main parameters considered in the model. Results remained robust, with estimates that remain within the confidence intervals reported in the main text. We used PWID as an illustrative example.

6.1 IMPACT OF SPONTANEOUS CLEARANCE IN THE ACUTE PHASE

We assessed the impact of a different percentage of spontaneous clearance in the acute phase, which is set to 24.4% in the main text, based on estimates from the literature (**Table 2**). Assuming 0% or 50% spontaneous

clearance produced differences in estimated incidence (e.g., the median incidence varied by approximately 30%). However, these differences remained within the confidence intervals of the main analyses (**Figure S9**).

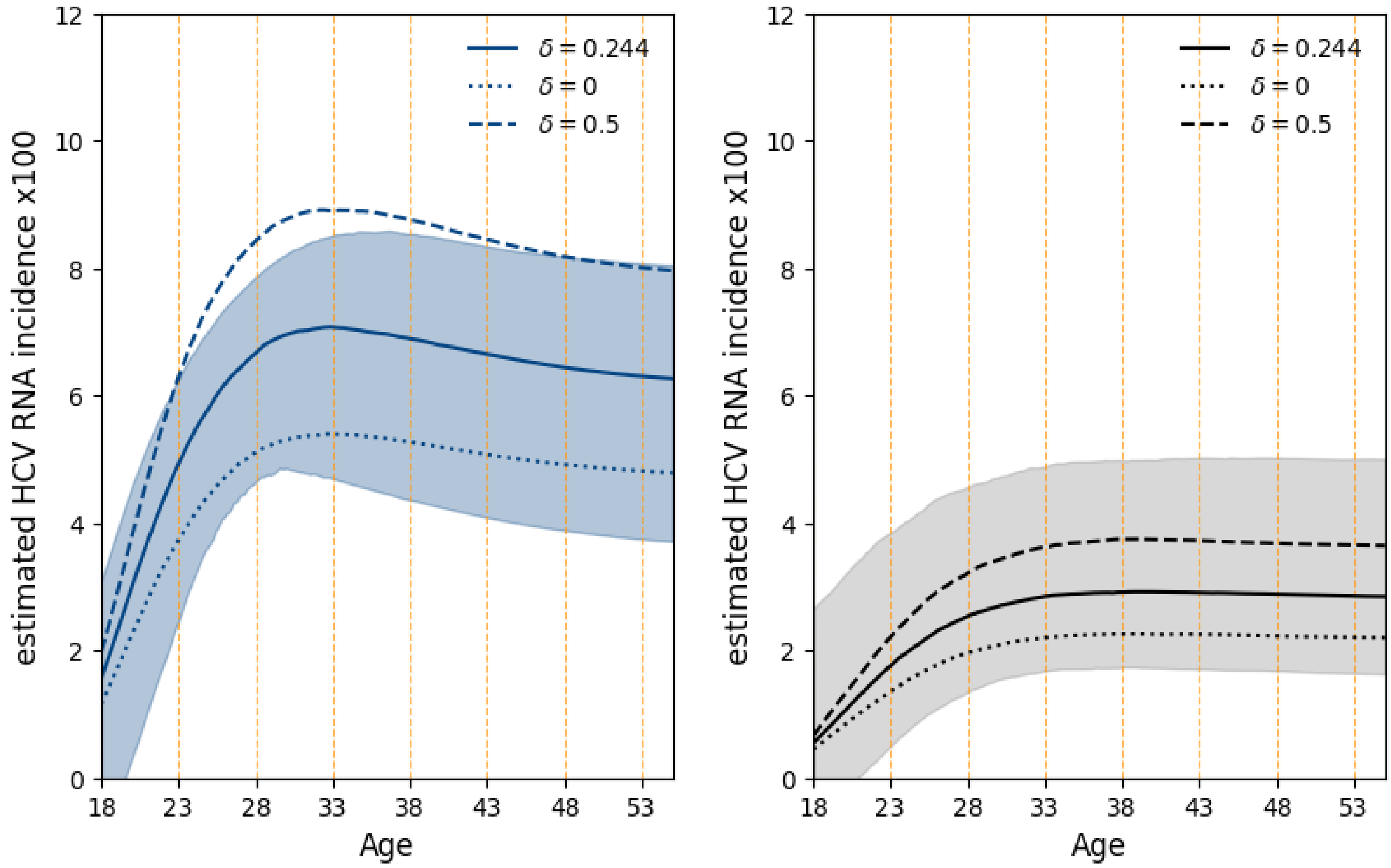

Figure S9. Impact of spontaneous clearance in the acute phase on HCV RNA incidence in PWID population. (Left) HCV RNA incidence estimated as a function of age for 2013. The solid line represents the main parameter value (δ = 24.4%), the dotted line corresponds to δ = 0%, and the dashed line represents δ = 50%. Shaded areas indicate 95% confidence intervals (CIs) of the main parameter value. Vertical orange lines serve as visual guides. **(Right)** as **(Left)** for 2023.

6.2 Impact of the percentage of population on DAA treatments

Figure S10 reports the sensitivity on the percentage of PWID receiving DAA treatment. We tested 0% and 50% of the population, with respect to 23% assumed in the main text. We found no significant differences in incidence of antibody, given the rare phenomenon of seroreversion, playing a minor role. For HCV RNA, the estimates do not affect the general results presented in the main text. The fact of moving more infected individuals to being susceptible again, without providing them with any immunity, is evident in the curves in the right panel of **Figure S10**.

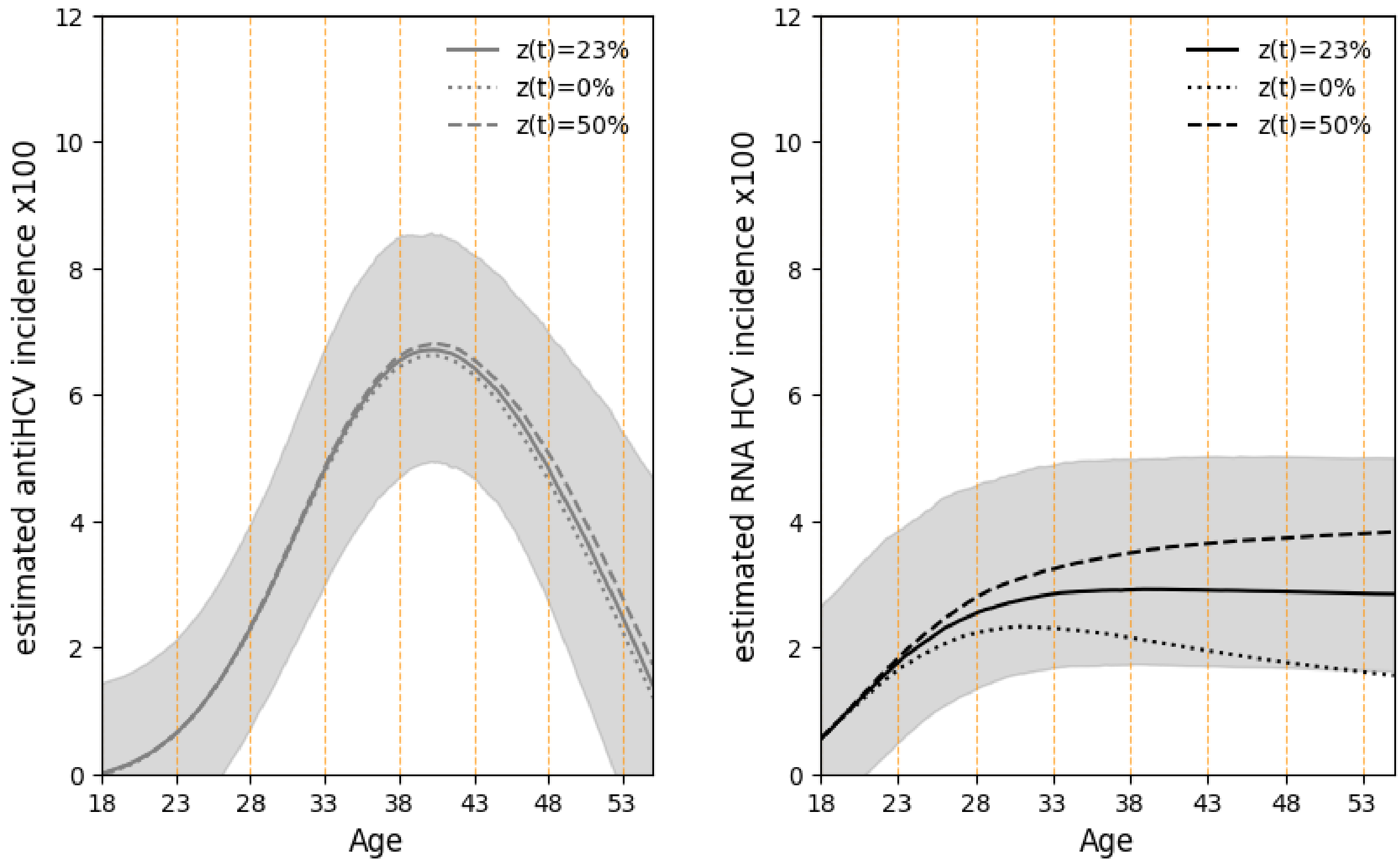

Figure S10. Impact of the percentage of DAA-treated people on anti-HCV and HCV RNA incidence in PWID population. (Left) Anti-HCV incidence estimated as a function of age for 2023. The solid line represents the main parameter value (z(t) = 23%), the dotted line corresponds to z(t) = 0%, and the dashed line represents z(t) = 50%. Shaded areas indicate 95% confidence intervals of the main parameter value. Vertical orange lines serve as visual guides. **(Right)** as **(Left)** for HCV RNA.

7. List of Coquelicot partner structures acknowledged

2004 :

Paris: Nova Dona, Pierre Nicolle, Horizons, La Corde Raide, Charonne, SOS DI, Drogues Jeunesses, Moreau de Tours, Murger, Cassini, Emergence, EGO, MDM, Aides, La Terrasse, Marmottan, Dr Majerloch, Dr Margelisch, Dr Molinier, Dr Joory, Dr Ekue, Dr Bezanson, Dr Demoor, Dr Francoz, Dr Gandour

Lille: Boris Vian, CITD, Espace du Possible, Le Cèdre Bleu, AIDES, Dr Marousez, Dr Heunet, Dr Messaadi, Dr Debomy, Dr Matton, Dr Riff, Dr Flageollet

Strasbourg: Espace Indépendance, ALT, Le fil d'Ariane, Dr Bernard-Henry, Dr Verhnes, Dr Garnier, Dr Gras, Dr Michel, Dr Rolland Jacquemin, Dr Sellam

Bordeaux: MDM, CEID, Montesquieu, Pellegrin, Parlement Saint-Pierre, Dr Lorans, Dr Quilichini, Dr Sagardoy, Dr Thibaut, Dr De Ducla, Dr Canovas

Marseille: AMPTA, MDM, AIDES, Sainte-Marguerite, Intersecteur des pharmacodépendances, SOS DI, Le Cabanon, Dr Beria, Dr Federici, Dr Philibert, Dr Martin, Dr Robichon, Dr Brun, Dr Hakoun, Dr Hariton, Dr Chiappe

2013:

Bordeaux : le département d'addictologie de l'hôpital de Bordeaux, Le Local - La Case, le PES - La Case, la boutique Planterose - CEID, le centre méthadone – CEID, le PES - CEID, le CSAPA Saint-Pierre, le CSAPA généraliste résidentiel de Bègles, le service d'appartements thérapeutiques – CEID.

Lille : le Caarud Aides 59, le Caarud JOAO et ambulatoire – Reagir, le centre de soins Reagir – Service Méthadone, la permanence d'accueil et d'orientation du centre de soins – Reagir, le centre d'hébergement d'urgence de nuit pour UD - Le Cèdre Bleu, le CSAPA le Cèdre Bleu, le service d'appartements thérapeutiques du Cèdre Bleu, la Clairière, le Point de repère ABEJ, le centre hospitalier la Fraternité, l'accueil Spiritek, le point accueil Oxygène CIPD, l'unité mobile Oxygène CIPD, l'Âtre, l'Ellipse – ADNSEA, le Relais – ADNSEA, le Relais – ADNSEA, le Prélude – ADNSEA, l'Intermezzo – ADNSEA, le Concerto – ADNSEA, Boris Vian.
Strasbourg : La Robertsau - ALT - Association de lutte contre la toxicomanie, Ste Catherine - ALT - Association de lutte contre la toxicomanie, le RMA - Réseau maternité et addiction, le Fil d'Ariane, le Caarud - Espace Indépendance, le Bus - Espace indépendance, le CSAPA - Espace Indépendance.
Marseille : AMPTA - CSAPA Ouest Etang de Berre, le CSAPA - Villa Floreal, le CSAPA Casanova - SOS drogue international, TIPI, TIPI Vian, Asud, Point Marseille, SOS DI Point Marseille, Sleep In - Consultation - SOS DI, Sleep In - Nuit - SOS DI, La Corniche - SOS DI, CSAPA Bus méthadone - Association Bus 31/32, CSAPA PES (Accueil + Bus 31/32), Accueil MDM - Association Bus 31/32, Protox, CSAPA Nationale – AMPTA, Hébergement CSAPA Nationale – AMPTA, Hébergement CSAPA Nationale – AMPTA, Puget Corderie / Pôle addictions.
Paris : 110 - Les Halles - SOS drogue international (CSAPA), Le Kaléidoscope - SOS drogue international (Caarud), Emergence Espace Tolbiac CSAPA, Cité le village - Association des cités du Secours catholique, Hôpital Sainte-Anne (service d'addictologie) - Moreau de Tours, Hôpital Marmottan (CSAPA) - Addictologie, Hôpital Marmottan (service hospitalier), Hôpital Marmottan - Médecine générale, Cassini (CSAPA) - Le Centre méthadone, Le Centre Cassini CSAPA, La terrasse (Caarud) - Boréal, La terrasse (Caarud) - Équipe de rue de Boréal, La terrasse (CSAPA) - Casat La terrasse, le Centre méthadone La terrasse (CSAPA), La corde raide- Centre de soins (CSAPA), La corde raide (CSAPA) - Unité médicale, le Bus Gaïa (CSAPA), Gaïa (Caarud), Adaje (CSAPA), EGO (CSAPA), Ego Centre d'accueil (Caarud), Ego Step (Caarud), Ego - Equipe mobile Step, Safe, Monte Cristo HEGP (CSAPA), Nova dona (CSAPA), Nova dona (Caarud), Pierre Nicole - Unité de substitution (CSAPA), Pierre Nicole – CTR, ATR Pierre Nicole, Pierre Nicole - Pass' justice, Sleep-in - SOS drogue international (CSAPA), Sleep-in jour - SOS drogue international (CSAPA), Confluences - appartements thérapeutiques SOS drogue Comportements à risque et prévention dans des populations particulièrement exposées au VIH, aux IST et aux hépatites BEH 39-40 | 26 novembre 2013 | 509 international (CSAPA), Confluences - Service accueil - SOS drogue international (CSAPA), Coordination thérapeutique - SOS drogue international (H), CSAPA Charonne, CHS (centre d'hébergement de stabilisation) Charonne, ACT (appartements de coordination thérapeutique) Charonne, ATR (appartements thérapeutiques relais) – Charonne, Charonne (boutique mixte-Caarud), Charonne (boutique femmes Caarud), PES Charonne, Charonne (Caarud) – Beaurepaire, Aides 75 (PES) – AUDVIH, Horizons (CSAPA), Hôpital Fernand Widal (CSAPA) - espace Murger

2023:
Lyon : Le Caarud d'Oppelia Aria, Le Csapa de l'association Lyade ARHM, Le Caarud et auprès de l'équipe mobile de Pause diabolo, La permanence d'accueil de l'association Keep Smiling, Le Csapa des Hospices Civils de Lyon, Le Csapa de l'Hôpital de La Croix rousse, Le Csapa d'Oppelia Aria à Villeurbanne, Le Csapa de l'association Lyade ARHM à Venissieux
Clermont-Ferrand : Le Csapa de l'Association Nationale de Prévention en Alcoologie et Addictologie, Le Csapa Satis et le dispositif d'hospitalisation du CHU, Le Caarud de l'association Aides
Annemasse : Le Caarud et le Csapa Apreto.
Bordeaux: Csapa ambulatoire du Service d'addictologie du centre hospitalier Charles-Perrens, Le Csapa Maurice-Serisé (Comité d'étude et d'information sur la drogue et les addictions – CEID addictions), Le Caarud Planterose (CEID addictions), Le Caarud Saint-James (association La Case), Les dispositifs de Csapa résidentiel en appartements de coordination thérapeutique/dans le diffus du CEID, Le dispositif d'hébergement en appartements de coordination thérapeutique/dans le diffus géré par l'association La Case, et auprès des équipes mobiles du CEID et de La Case.

Strasbourg: Le Caarud, le Csapa et la salle de consommation à moindres risques (SCMR) gérés par l'association Ithaque, Le Csapa, le centre thérapeutique résidentiel et le dispositif d'appartements thérapeutiques de l'Association de lutte contre la toxicomanie (ALT), Le Csapa du service d'addictologie des Hôpitaux universitaires de Strasbourg, et un dispositif de réduction des risques de proximité mené en partenariat par les associations Ithaque, ALT et OPI.
Cayenne: Le Caarud, le Relais santé drogues et auprès de l'équipe mobile de l'association Relais Drogues Solidarité, L'association guyanaise de réduction des risques,
Saint-Laurent-du-Maron: Le Caarud, le Csapa auprès de l'équipe mobile de l'association Akatij,
Matoury : Le Caarud de l'association Relais Drogues Solidarité;
Roura: Le dispositif d'hébergement du groupe SOS Solidarités.
Lille: Le Caarud, le Csapa et le Sleep'in de l'association Cédragir,Le Caarud de l'association Aides, Le Caarud Spiritek, Le Csapa Boris-Vian (EPSM Pôle Addictologie), Le Caarud Ellipse de la Sauvegarde du Nord, Le service d'Addictologie du CHU de Lille, Le Csapa de l'accueil temporaire réinsertion (Atre) de l'Association d'aide aux détenus nécessitant des soins médico-psychologiques (ADNSMP), Le Caarud Entractes, Le Caarud Point de repère de l'association ABEJ Solidarité,
Faches-Thumesnil: Le Caarud Oxygène,
Montataire: Le Caarud Le Relais de l'association Sato-Picardie
Tourcoing: Le Caarud, le Csapa et le Sleep-in JOAO de l'association Cédragir
Amiens: Le Caarud La Parenthèse et le Csapa du Mail,
Creil: Le Csapa de Creil.
Paris : Le Spot Beaumarchais (75003) & le Caarud Antenne Paris (75019) de l'association Aides, Le Csapa de l'Hôpital Georges Pompidou (75015), Le Csapa de l'Hôpital Lariboisière-Fernand Widal (75010), Le Csapa Emergence Espace Tolbiac (ETT) (75013) de l'association mutualité fonction publique, Le Csapa & Caarud Ego-STEP, Espace de repos La Chapelle, Equipe mobile & Maraude (75018), Hébergement ASSORE et PHASE (75019) de l'association Aurore, Le Csapa Horizon de l'association Estrella (75010), La HSA, Caarud, Csapa, bus méthadone et équipe Mobile de l'association Gaïa (75010, 75011), Le Csapa Marmottan (75017), le Csapa Saint Anne (75014), le Csapa la Terrasse (75018) et le Caarud Boréal (75019) du GHU Paris Psychiatrie Neurosciences , Le Caarud Kaléidoscope (75019) et les Csapa (75002,75013,75018) du Groupe SOS Solidarités, Le Csapa Pierre Nicole et l'hébergement de l'association la Croix Rouge Française (75005), Le Csapa & Caarud de l'association Nova Dona, Le Csapa, Caarud, équipe mobile, hébergements et Espace femmes de l'association Oppelia Charonne (75010, 75012, 75013, 75018 & 75019), L'équipe mobile de l'association SAFE (75015)
Bondy: Le Csapa Boucebci de l'Assistance Publique Hôpitaux de Paris (93140), Le Caarud YUCCA de l'association Groupe SOS Solidarités
Aulnay sous-bois: Le Caarud CHI Robert Ballanger de l'association Aurore (93600), Le Csapa Gainville de l'hôpital Robert Ballanger
Montreuil: Le Csapa Mosaïque du CHI André Grégoire, Le Caarud et Hébergement Montreuil
Montfermeil: Le Csapa Valjean de l'hôpital de Montfermeil GHT Grand Paris Nord Est
Saint Denis: Le Csapa Le Corbillon de l'hôpital Casanova GHT St Denis-Gonesse, le Caarud et Equipe Mobile Saint-Denis de l'association Proses
Juvisy sur Orge: Le Caarud Fressonne d'Oppelia
Athis-Mons: RESSOURCES – Le CSAPA de l'association Ressources
Nantes: Le Caarud et le Csapa gérés par l'association Oppelia, Le Csapa de l'Association Les Absyades, Le service d'addictologie du CHU de Nantes.
Marseille: Le Csapa du Groupe SOS Solidarités Covid, Le Caarud du Groupe SOS Solidarités, Le Csapa Puget Corderie, Le Caarud et le Csapa/Bus Méthadone de l'association Bus 31/32, Le Csapa Le Sémaphore (association Addiction Méditerranée), Le Csapa et le Caarud Protox (AP-HM), Le dispositif d'hébergement / Csapa Point Marseille du Groupe SOS Solidarités, Le Caarud Asud-le Tipi à Marseille, Auprès des équipes mobiles d'Asud/Mars Say Yeah.
Nice : Le Csapa du CHU L'archet, Le Csapa, le Caarud Imp'actes et auprès de l'équipe mobile de la Fondation de Nice, Le Caarud du Groupe SOS Solidarités, Le Caarud Lou Passagin (Groupe SOS Solidarités Info 3 Services), Le Csapa Emergence (Groupe SOS Solidarités).
Avignon : Le Caarud Le Patio et le Csapa Ressources CAP14 (Groupe SOS Solidarités), Le Csapa Convergence (Anpaa 84), Le Caarud La Boutique, Le dispositif d'hébergement Le Relais (Groupe SOS Solidarités).

Montpellier : Le Csapa accompagnement, le Csapa UTTD, le service d'addictologie du service d'hospitalisation du CHU de Montpellier, Le Caarud et l'équipe mobile de l'association Réduire les risques, Le Caarud, l'antenne méthadone et l'équipe mobile Axess (groupe SOS Solidarité), Le Csapa AMT Arc-en-ciel, Le Csapa de l'association Anpaa 34.
Toulouse : Le Csapa Maurice-Dide du CHU de Toulouse, Le Csapa de l'Association régionale de prévention et d'aide face aux dépendances et aux exclusions (Arpade), Le Caarud Intermède, le Csapa et le dispositif d'appartement thérapeutique de l'association Clémence-Isaure), Le Caarud de l'association Aides.
Nîmes : Le Caarud et le Csapa Logos (association Apsa 30), Lieu d'accueil et équipe mobile d'Arap Rubis (Association de santé communautaire en milieu prostitutionnel), Le Csapa du CHU de Nîmes, Le Caarud et l'équipe mobile de l'association Asud.

REFERENCES

(1) Jauffret-Roustide, M. Monitoring HIV, HCV and HBV among People Who Use Drugs in France. A Reflective Feed-Back on a 25-Year Experiment Based on Mixed Methods. The ANRS-Coquelicot Survey. *Int. J. Drug Policy* **2024**, *136*, 104690. https://doi.org/10.1016/j.drugpo.2024.104690.
(2) Leon, L.; Jauffret-Roustide, M.; Le Strat, Y. Design-Based Inference in Time-Location Sampling. *Biostat. Oxf. Engl.* **2015**, *16* (3), 565–579. https://doi.org/10.1093/biostatistics/kxu061.
(3) Vaux, S.; Chevaliez, S.; Saboni, L.; Sauvage, C.; Sommen, C.; Barin, F.; Alexandre, A.; Jauffret-Roustide, M.; Lot, F.; Velter, A.; ANRS-Prevagay group. Prevalence of Hepatitis C Infection, Screening and Associated Factors among Men Who Have Sex with Men Attending Gay Venues: A Cross-Sectional Survey (PREVAGAY), France, 2015. *BMC Infect. Dis.* **2019**, *19* (1), 315. https://doi.org/10.1186/s12879-019-3945-z.
(4) Leon, L.; Kasereka, S.; Barin, F.; Larsen, C.; Weill-Barillet, L.; Pascal, X.; Chevaliez, S.; Pillonel, J.; Jauffret-Roustide, M.; Strat, Y. L. Age- and Time-Dependent Prevalence and Incidence of Hepatitis C Virus Infection in Drug Users in France, 2004–2011: Model-Based Estimation from Two National Cross-Sectional Serosurveys. *Epidemiol. Infect.* **2016**, *145* (5), 895. https://doi.org/10.1017/S0950268816002934.
(5) Stefic, K.; Guinard, J.; Peytavin, G.; Saboni, L.; Sommen, C.; Sauvage, C.; Lot, F.; Laperche, S.; Velter, A.; Barin, F. Screening for Human Immunodeficiency Virus Infection by Use of a Fourth-Generation Antigen/Antibody Assay and Dried Blood Spots: In-Depth Analysis of Sensitivity and Performance Assessment in a Cross-Sectional Study. *J. Clin. Microbiol.* **2019**, *58* (1), e01645-19. https://doi.org/10.1128/JCM.01645-19.
(6) Léon, L. Estimation de prévalences et d'incidences à partir d'enquêtes épidémiologiques transversales répétées auprès de populations difficiles d'accès : Application au virus de l'hépatite C chez les usagers de drogues en France. phdthesis, Université Paris Saclay (COmUE), **2016**. https://theses.hal.science/tel-01635287 (accessed 2025-02-26).
(7) Maher, L.; Wand, H.; Heard, S.; Starr, M.; Zolala, F.; Kemp, R.; Cunningham, P.; Drummer, H.; Hellard, M.; Kaldor, J.; Dore, G. Utilising Integrated Bio-Behavioural Surveillance (IBBS) to Investigate Declining Hepatitis C Antibody Prevalence among People Who Inject Drugs in the Australian Needle and Syringe Program Survey. *Int. J. Drug Policy* **2024**, *131*, 104545. https://doi.org/10.1016/j.drugpo.2024.104545.
(8) Pitcher, A. B.; Borquez, A.; Skaathun, B.; Martin, N. K. Mathematical Modeling of Hepatitis C Virus (HCV) Prevention among People Who Inject Drugs: A Review of the Literature and Insights for Elimination Strategies. *J. Theor. Biol.* **2018**, *481*, 194. https://doi.org/10.1016/j.jtbi.2018.11.013.
(9) Martin, N. K.; Vickerman, P.; Hickman, M. Mathematical Modelling of Hepatitis C Treatment for Injecting Drug Users. *J. Theor. Biol.* **2011**, *274* (1), 58–66. https://doi.org/10.1016/j.jtbi.2010.12.041.
(10) Helikumi, M.; Mushanyu, J.; Mhlanga, A. A Mathematical Model of HCV Transmission Dynamics with Sex Stratification and Environmental Effects. *PLOS ONE* **2025**, *20* (12), e0336374. https://doi.org/10.1371/journal.pone.0336374.
(11) Elbasha, E. H. Model for Hepatitis C Virus Transmissions. *Math. Biosci. Eng. MBE* **2013**, *10* (4), 1045–1065. https://doi.org/10.3934/mbe.2013.10.1045.
(12) Jothi, N. K.; M, J.; D, S.; V, V.; Mattuvarkuzhali, C. Modeling Hepatitis C Transmission to Inform Public Health Strategies and Long-Term Control. *Trends Immunother.* **2025**, 25–44. https://doi.org/10.54963/ti.v9i4.1347.

(13) Donnadieu-Rigole, H. *Sympo AFEF ANRS - Etude ANRS*. https://afef.asso.fr/sympo-afef-anrs-etude-anrs-icone-2-un-modele-de-depistage-pour-les-usagers-de-drogues-pour-lutter-contre-lhepatite-c/ (accessed 2025-05-27).
