# Supplementary material for: HCV incidence and prevalence trends among people who use drugs in France 2004 to 2023
Source: Nat Commun. 2026 Oct 11;17:11525. doi: 10.1038/s41467-026-78598-3 (PMC13745531; doi:10.1038/s41467-026-78598-3)
Supplement: Supplementary file 2 [file 41467_2026_78598_MOESM2_ESM.pdf]

nature portfolio

Corresponding author(s): Chiara Elisa Sabbatini

Last updated by author(s): August 28, 2026

Reporting Summary

Nature Portfolio wishes to improve the reproducibility of the work that we publish. This form provides structure for consistency and transparency in reporting. For further information on Nature Portfolio policies, see our Editorial Policies and the Editorial Policy Checklist.

Statistics

For all statistical analyses, confirm that the following items are present in the figure legend, table legend, main text, or Methods section.

n/a	Confirmed	
☐	☒	The exact sample size (*n*) for each experimental group/condition, given as a discrete number and unit of measurement
☐	☒	A statement on whether measurements were taken from distinct samples or whether the same sample was measured repeatedly
☐	☒	The statistical test(s) used AND whether they are one- or two-sided<br>*Only common tests should be described solely by name; describe more complex techniques in the Methods section.*
☐	☒	A description of all covariates tested
☐	☒	A description of any assumptions or corrections, such as tests of normality and adjustment for multiple comparisons
☐	☒	A full description of the statistical parameters including central tendency (e.g. means) or other basic estimates (e.g. regression coefficient) AND variation (e.g. standard deviation) or associated estimates of uncertainty (e.g. confidence intervals)
☐	☒	For null hypothesis testing, the test statistic (e.g. *F*, *t*, *r*) with confidence intervals, effect sizes, degrees of freedom and *P* value noted<br>*Give P values as exact values whenever suitable.*
☒	☐	For Bayesian analysis, information on the choice of priors and Markov chain Monte Carlo settings
☐	☒	For hierarchical and complex designs, identification of the appropriate level for tests and full reporting of outcomes
☒	☐	Estimates of effect sizes (e.g. Cohen's *d*, Pearson's *r*), indicating how they were calculated

Our web collection on statistics for biologists contains articles on many of the points above.

Software and code

Policy information about availability of computer code

Data collection	Computer code was not used to collect data
Data analysis	Analyses were carried out in R version 4.3.3 and STATA 18.0.<br>Code is publicly available at: https://codeberg.org/SpF_DATA/HCV_among_PWUD_Coquelicot.git<br>For reproducibility, the version corresponding to this study is deposited in Zenodo: https://zenodo.org/records/22054853

For manuscripts utilizing custom algorithms or software that are central to the research but not yet described in published literature, software must be made available to editors and reviewers. We strongly encourage code deposition in a community repository (e.g. GitHub). See the Nature Portfolio guidelines for submitting code & software for further information.

Data

Policy information about availability of data

All manuscripts must include a data availability statement. This statement should provide the following information, where applicable:

- Accession codes, unique identifiers, or web links for publicly available datasets
- A description of any restrictions on data availability
- For clinical datasets or third party data, please ensure that the statement adheres to our policy

ANRS-Coquelicot data are confidential and cannot be shared due to ethical restrictions. Researchers wishing to have more information about the data provided in the article are invited to contact Marie Jauffret-Roustide and Chiara E. Sabbatini, at the following addresses: marie.jauffret-roustide@inserm.fr; chiara.sabbatini@santepubliquefrance.fr. All aggregate results, prevalence, and incidence data generated in this study are included in the accompanying Source

Data file. Source Data are provided with this paper.

Research involving human participants, their data, or biological material

Policy information about studies with human participants or human data. See also policy information about sex, gender (identity/presentation), and sexual orientation and race, ethnicity and racism.

Reporting on sex and gender	Gender was determined via self-report. Participants self-identified as male or female; no non-binary or gender-diverse categories were collected. We reported descriptive statistics on gender but performed no gender-based analyses, as this represents a distinct research question deserving full investigation and thus falls beyond the scope of this article.
Reporting on race, ethnicity, or other socially relevant groupings	Collecting data on race is not allowed in France. Ethno-linguistic affiliation (French speaking versus non French speaking) was used as a proxy for migration based on self-report. Additionally, we gathered information on the birth country of the participants, their parents, and their grandparents. However, we do not interpret these variables as indicators of ethnicity or race.
Population characteristics	Participants were people who use drugs or people who inject drugs (PWUD, PWID) who attend harm reduction services and addiction treatment centers, aged 18 and older, recruited in France during cross-sectional surveys in 2004, 2013, and 2023. Covariates included age, gender, drug use patterns (e.g. injection history, crack use), incarceration history, HIV and HCV status, and self-reported access to housing. These characteristics are described in the Methods section and in Supplemental Information
Recruitment	Participants were recruited in community settings and harm reduction sites on a voluntary basis.
Ethics oversight	All participants provided informed consent. Only simulated data are included in this repository; no personal or identifying information is shared.

Note that full information on the approval of the study protocol must also be provided in the manuscript.

Field-specific reporting

Please select the one below that is the best fit for your research. If you are not sure, read the appropriate sections before making your selection.

[x] Life sciences [ ] Behavioural & social sciences [ ] Ecological, evolutionary & environmental sciences

For a reference copy of the document with all sections, see nature.com/documents/nr-reporting-summary-flat.pdf

Life sciences study design

All studies must disclose on these points even when the disclosure is negative.

Sample size	Sample sizes were determined based on available population in each survey year (2004, 2013, and 2023).
Data exclusions	Participants with missing data on key variables (e.g., anti-HCV status, age) were excluded from relevant analyses. These exclusions are specified and performed consistently across datasets. Details can be found in the Methods section and in Supplemental Information
Replication	The study used three independent cross-sectional surveys conducted in 2004, 2013, and 2023. Analyses were replicated across these timepoints to estimate trends in antiHCV, HCV RNA prevalence and incidence. Additionally, we used bootstrap resampling (2,000 iterations) to generate confidence intervals and assess the stability of incidence estimates.
Randomization	Our study is observational and population-based in nature, and involves no intervention. Randomization of subjects is therefore not applicable.
Blinding	Our study is observational and population-based in nature, and involves no intervention. Blinding techniques are therefore not applicable.

Reporting for specific materials, systems and methods

We require information from authors about some types of materials, experimental systems and methods used in many studies. Here, indicate whether each material, system or method listed is relevant to your study. If you are not sure if a list item applies to your research, read the appropriate section before selecting a response.

Materials & experimental systems

n/a	Involved in the study	
☐	☒	Antibodies
☒	☐	Eukaryotic cell lines
☒	☐	Palaeontology and archaeology
☒	☐	Animals and other organisms
☒	☐	Clinical data
☒	☐	Dual use research of concern
☒	☐	Plants

Methods

n/a	Involved in the study	
☒	☐	ChIP-seq
☒	☐	Flow cytometry
☒	☐	MRI-based neuroimaging

Antibodies

Antibodies used	FDA-approved commercial kits were used for the anti-HCV and anti-HIV ELISAs according to manufacturer instructions (Genscreen ULTRA HIV Ag/Ab catalog number 72386, Monolisa Anti-HCV PLUS V3 catalog number 72340).
Validation	Validation relies on the manufacturer clinical validation data for the standardized diagnostic ELISA kits.

Plants

Seed stocks	-
Novel plant genotypes	-
Authentication	-
