# Supplementary material for: HCV incidence and prevalence trends among people who use drugs in France 2004 to 2023
Source: Nat Commun. 2026 Oct 11;17:11525. doi: 10.1038/s41467-026-78598-3 (PMC13745531; doi:10.1038/s41467-026-78598-3)
Supplement: Supplementary file 3 [file 41467_2026_78598_MOESM3_ESM.pdf]

nature portfolio

Peer Review File

HCV incidence and prevalence trends among people who use drugs in France 2004 to 2023

Corresponding Author: Dr Chiara Sabbatini

This file contains all reviewer reports in order by version, followed by all author rebuttals in order by version.

Version 0:

Reviewer comments:

Reviewer #1

(Remarks to the Author)
Thank you for the opportunity to review this manuscript. This study provides important insights into HCV elimination in France, and the findings will be of relevance for many researchers and practitioners who work in HCV research. I also ran the code and it worked perfectly. Below are a few comments:
1. It is interesting that there was only an average of about ten participants per service, can you provide more information about the distribution of recruitment? Are there some services that do the bulk of recruitment?
2. Further to this, did researchers visit services once, using a random time-service slot?
3. Were 20% of DBS missing in 2023 because participants refused testing, or because of an invalid test result?
4. How many participants were excluded from the 2013 and 2023 surveys after restricting the analysis to Bordeaux, Lille, Marseille, Paris, and Strasbourg?
5. Suggest using colour-blind friendly palettes in figures, avoiding combining green and red
6. I'm confused about what figure 1. (c) is showing. Is the percentage of participants who were PWID in 2023 around 40%, and the percentage who were non-PWID around 25%? Shouldn't these percentages add to 100%? Can you please clarify
7. The model parameters look reasonable, although should there also be a parameter for the death rate among HCV infected?
8. Can you clarify what the DAA treatment parameter includes? Is it that 23% of participants reported completing DAA treatment in 2023? I looked at reference 12 and couldn't find any information on this

(Remarks on code availability)
I ran the code and it worked fine and was easy to follow

Reviewer #2

(Remarks to the Author)
The paper considers an important question of whether France has progressed to achieving HCV elimination among PWID through the scale up in HCV treatment and changes in other interventions. The paper uses data from three large surveys, with modelling being used to translate data on prevalence trends into incidence trends. I have the following comments which need addressing:
1. In the introduction, when you say you increase accuracy of estimates using HCV RNA-based data, I think you need to say more clearly what you mean. I think the RNA data is used to estimate real decreases in HCV transmission, which HCV Ab prevalence is not so good at doing.
2. What bias is introduced by just recruiting from service settings. What proportion of people do not attend these settings and so would not be represented in your surveys. Does this make your projections of impact optimistic? This should be discussed in a limitations section.
3. It is good to see the comparisons between rounds being limited to the common cities. However, how do you think other changes in scope and eligibility to include crack smoking and including different languages, likely affected your results?
4. The model for using antibody prevalence data seems ok, although I couldn't see mu2 defined in table 2.
5. More explanation is needed on how you go from the equations in line 186 to equations on line 189 in the supplementary materials. I can see some of what you may have done but more of the steps need to be include. Also, mu2 does not appear in the equations on line 189. You have also gone from ODE to PDE with no explanation.

6. You say similar calculations are used for the HCV RNA model but no details are given. I think this needs to be included to understand what you did because the model will be different in some important ways. Indeed, I am unsure if the derivative of the prevalence will still be a good representation of the incidence because treatment directly effects the prevalence profile. It is important to see how the authors accounted for the rate of treatment other time.
7. I am surprised antibody prevalence has decreased between the survey rounds. This has not been observed in other settings like England and Scotland. In those settings antibody prevalence has stayed stable but RNA prevalence has decreased considerably. Only in Australia, have they seen large decreases in antibody prevalence since the scale up in treatment and they decided that this was due to more rapid rates of loss of antibody response, with the decrease disappearing when they accounted for people who had lost their Ab response after treatment. See the paper https://pubmed.ncbi.nlm.nih.gov/39159531/ (maher IJDP 2024). Are you sure your decrease in prevalence reflects a decrease in incidence and not something else? Do you have any data to do a similar correction to what they did in Australia? They looked to see what proportion of those with negative antibody response reported ever having been HCV treated.
8. The authors discuss that the difference between antibody and RNA prevalence remained substantial over time. It would be good to see how the prevalence of RNA positivity among people with positive antibody response changes over time. In other settings this percentage has decreased dramatically because of the scale up in treatment.
9. I think the difference between the estimates of incidence from the antibody prevalence data and the RNA prevalence data needs careful consideration. Why do you think it is different?
10. Did the modelling account for the changing age distribution of the population in each survey year and what that means for the recruitment rate of new PWID. Similarly, did you control for changes in the population distribution when you assessed the changes in antibody prevalence and RNA prevalence over time.
11. I was also surprised that incidence increases with age – this is contrary to a recent systematic review that found incidence to be highest in young PWID (https://pubmed.ncbi.nlm.nih.gov/36996853/). It would be good to change figure 2 so that it uses a more standard non logarithmic scale for showing prevalence from 0 to 100% on the y axis. This would help the reader see how the prevalence gradient increases over age up to the ages stated in the text.
Minor comments:
1. Two sentences in a row in introduction second paragraph start with however. I would suggest removing the however at the start of the second sentence
2. Swop ‘ones' for surveys when first mention ANRS-Coquelicot in the introduction
3. Swop evaluate with evaluating in the last line of introduction

(Remarks on code availability)
I reviewed the modelling maths which was in insufficient detail.

Reviewer #3

(Remarks to the Author)
In this manuscript the authors apply regression and dynamic compartmental modelling methods to data from three surveys conducted in 5 French cities spaced approximately 10 years apart, to estimate HCV prevalence and incidence over time. The prevalence of HCV positivity (both antibody and RNA) declined over time, and in particular HCV incidence among PWID was estimated to have decreased to 2.4 per 100 person-years, consistent with encouraging progress towards the WHO's 2030 HCV elimination targets. The authors' findings provide a useful addition to the evidence base on the evaluation of progress towards achievement of HCV elimination within the high-risk population of PWUD.

General comments:

I missed discussion regarding how the authors'results fit within the international context. In any eventual revision, I recommend adding some text comparing/contrasting the current estimates for PWUD in France with evaluation of progress towards the WHO targets reported by other countries. Although an English modelling study is cited (Ward et al., 2025), I believe that the reader would benefit from additional international discussion.

The principal limitations of the modelling techniques are adequately documented. Like similar modelling studies, the modelled estimates of HCV prevalence and incidence do depend on important assumptions and parameter values derived from the data source, some of which may be subject to bias and so could affect the conclusions.

Is the effect of DAA treatment on estimated HCV RNA incidence realistically modelled? As infected individuals who are successfully treated simply re-enter the susceptible compartment, the assumption is that there no modification of the incidence rate of re-infection. But in reality, DAA treatment is accompanied with education and harm reduction initiatives that could lead to reduced re-infection rates. In contrast, increased reinfection rates could be observed as a consequence of a growing susceptible pool of PWUD/PWID following successful treatment and/or reduced rates of re-testing, and any increases may be setting/population specific (see e.g. Yeung et al., 2022, J Hep). The authors should discuss this potential limitation further.

Specific comments:

L96-97: The authors 'note that, regardless of age, antibody prevalence significantly decreases between 2004, 2013, and

2023', and modelled HCV Ab incidence declined over time (consistent with declining prevalence in a study population consisting of different samples each survey round), but the reason for this is not discussed, at least in the main text. In 5.2 of the Suppl ageing and mortality is mentioned. The authors may wish to consider adding a sentence to the main text, rather than locating this only in this Suppl.

L112-114 The modelled peak incidence at relatively older ages (36-41 years) is interesting, as in contrast to previous studies of PWID populations where acquisition of infection occurs at a much younger age (possibly driven by riskier injection practices). However, in 5.1 of the Suppl the authors note that exposure in their younger-age population could well differ from the older studies regarding access to sterile equipment. As with the preceding comment, adding a sentence to the main text should be considered.

Suppl L191-193: Is there an error in notation? lambda(a,t) is first used to denote incidence of HCV infection, but in line 191 it is also used for prevalence, "we obtain the expression for prevalence for a given time t [as lambda(a,t) = ...]". Please edit if necessary.

Suppl section 4.2: Can you justify why a bootstrapped procedure without replacement was used to determine 95% CIs around outcomes, rather than bootstrapping involving sampling with replacement? The option chosen may lead to underestimation of the width of the 95% CI.

Suppl L310-11: The sentence "Results remained robust: assuming 0% or 50% leads to differences in the ballpark of estimations" conveys the opposite of what I believe the author intended. Please edit (is it not correct this analysis suggests no substantial difference in estimated incidence when the spontaneous clearance percentage is varied?).

(Remarks on code availability)

Version 1:

Reviewer comments:

Reviewer #1

(Remarks to the Author)
Thanks for your thorough revisions. I am satisfied with the responses.

(Remarks on code availability)

Reviewer #2

(Remarks to the Author)
The authors have replied in detail to all my comments. My only remaining query is their response to comment 10. I agree that it his hard to find a reference population age distribuition, however it would be standard practise when comparing prevalence estimates to adjust for any socio-demographic differences. Because prevalence increases with age it would be important to estimate an adjusted prevalence ratio for each survey round compared to the 2004 survey round that adjusts for differences in age and other factors.

(Remarks on code availability)

Reviewer #3

(Remarks to the Author)
I commend the authors on their careful revision of this important manuscript. All of my previous comments have been satisfactorily addressed and I have no further substantive comments.

A very minor point, only noticed in this round : Fig S6 (left panel); the x-axis label is incorrect.

(Remarks on code availability)

Open Access This Peer Review File is licensed under a Creative Commons Attribution 4.0 International License, which permits use, sharing, adaptation, distribution and reproduction in any medium or format, as long as you give appropriate credit to the original author(s) and the source, provide a link to the Creative Commons license, and indicate if changes were made.
In cases where reviewers are anonymous, credit should be given to 'Anonymous Referee' and the source.
The images or other third party material in this Peer Review File are included in the article's Creative Commons license, unless indicated otherwise in a credit line to the material. If material is not included in the article's Creative Commons license and your intended use is not permitted by statutory regulation or exceeds the permitted use, you will need to obtain permission directly from the copyright holder.
To view a copy of this license, visit https://creativecommons.org/licenses/by/4.0/

Reviewer #1 (Remarks to the Author):

Thank you for the opportunity to review this manuscript. This study provides important insights into HCV elimination in France, and the findings will be of relevance for many researchers and practitioners who work in HCV research. I also ran the code and it worked perfectly. Below are a few comments:

We thank the referee for taking the time to review our manuscript and for providing detailed feedback. We appreciate their thorough evaluation and have carefully considered their comments that helped us to largely improve the quality and presentation of our work. We provide here below a point-by-point reply to all comments.

1. It is interesting that there was only an average of about ten participants per service, can you provide more information about the distribution of recruitment? Are there some services that do the bulk of recruitment?

The average of approximately ten participants per service masks a more heterogeneous pattern of recruitment across sites.

We have added a figure (**Figure S1**) in the revised **Supplementary Information (SI)** that illustrates the distribution of recruitment across services. As illustrated in the histogram (**Figure S1a–c**), most services contributed relatively small numbers of participants, typically fewer than 20. However, the distribution is right-skewed, with a limited number of services accounting for a substantial share of total recruitment. The boxplots (**Figure S1d–f**) further highlight this variability, showing the presence of high-recruiting outliers, particularly in larger urban settings such as Paris.

This heterogeneity likely reflects differences in service size, catchment area, accessibility, and local outreach strategies, with some centers serving as key recruitment hubs. Such variability is an inherent feature of harm reduction and addiction care settings and was accounted for through our comprehensive sampling design and survey weighting methodology. This pattern was consistent across survey waves, indicating a stable structural feature.

All the above elements are now better explained in the **SI** [lines 95-108]:

*“The data reveal a highly skewed distribution of recruitment of PWUD across services. As shown in **Figure S1a-c** most services contribute modestly to recruitment, typically enrolling fewer than 20 participants. However, a small number of services account for a large share of the total. This pattern remains consistent across all survey editions (2004, 2013, and 2023). The boxplots in **Figure S1d-f** further illustrate this disparity, particularly in cities like Paris, where certain services recruit between 50 and 140 participants. This heterogeneity likely reflects structural factors, such as service capacity, population density, or outreach strategies, with some centers serving as key recruitment hubs. Such variability is an inherent feature of harm reduction and addiction care.”*

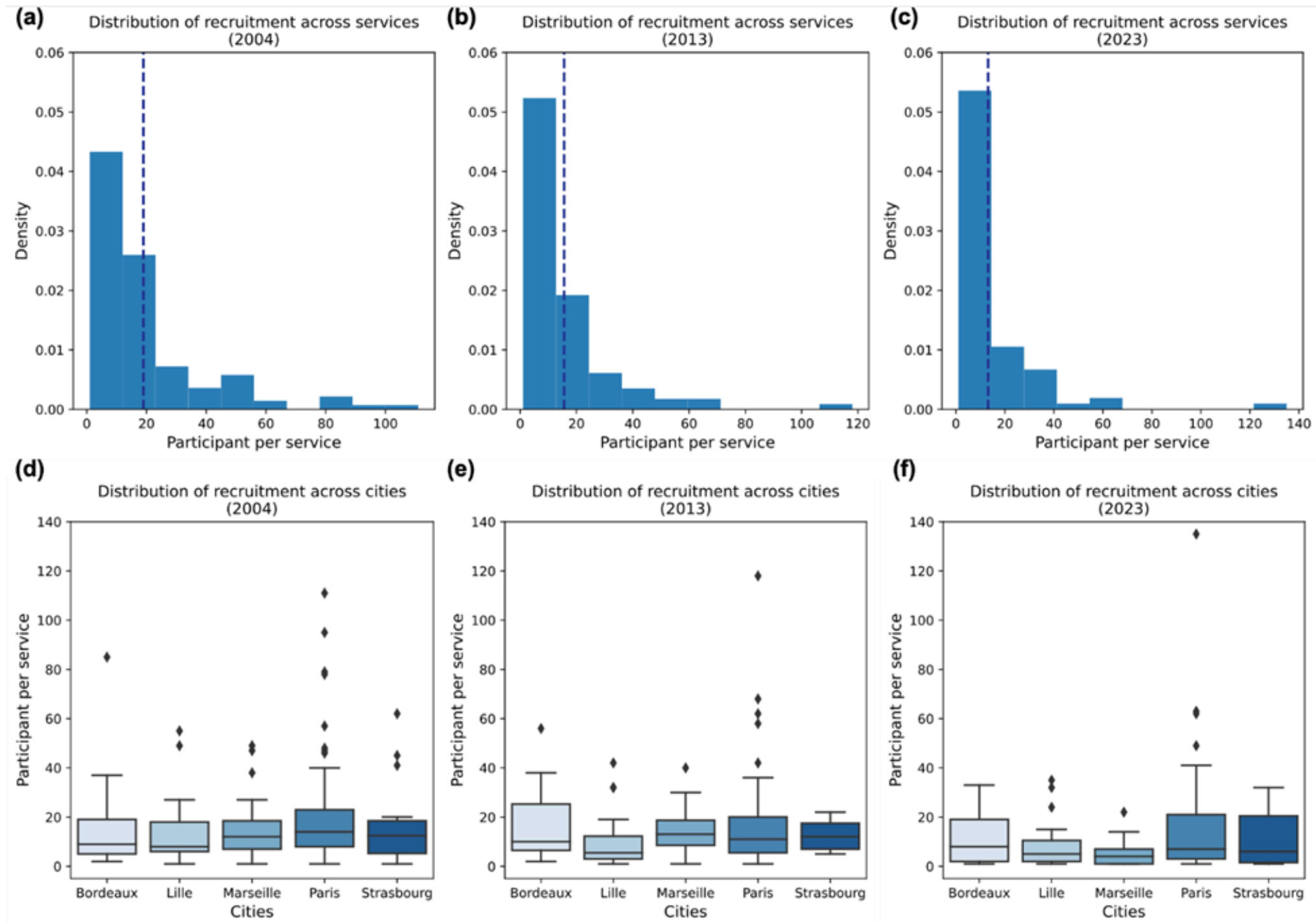

Figure S1. Distribution of recruitment across services and cities. (a-c) Histograms showing the distribution of recruitments among the services included in the study for 2004 **(a)**, 2013 **(b)** and 2023 **(c)**. The blue dashed line represents the mean value. **(d-f)** Boxplots showing the number of participants per service in Bordeaux, Lille, Marseille, Paris, and Strasbourg for 2004 **(d)**, 2013 **(e)** and 2023 **(f)**. Box plots represent the median (line in the middle of the box), interquartile range (box limits) and 2.5th and 97.5th percentiles (whiskers) and outliers for each city, illustrating the variability in recruitment. Paris exhibits the highest variability, with some services recruiting significantly more participants than others.

2. Further to this, did researchers visit services once, using a random time-service slot?

Thank you for this question. We did not restrict our visits to a single time slot per service. Instead, we implemented a two-stage Time-Location Sampling (TLS) design[1] to obtain a more representative sample.

First, we conducted an exhaustive inventory of services dedicated to PWUDs. For each participating service, we constructed a sampling frame that included all opening half-days during the survey period. From this list, we randomly selected a predefined number of half-day time slots. Consequently, some centers were visited multiple times when more than one of their time slots was selected.

Second, during each selected time slot, we applied systematic random sampling to invite individuals attending the service to participate. Participants were included if they provided written consent to be interviewed. Because this approach increases the likelihood of including individuals who attend services more frequently, we accounted for this in our analyses by applying weights based on frequency of venue attendance (FVA)[1].

The two-stage TLS approach enabled us to capture a broader and more diverse snapshot of the population across different days and times, rather than relying on a single visit that might not be representative. Further details are provided in the **SI** [lines 52–57]:

“We implemented a two-stage Time-Location Sampling (TLS) design to obtain a representative sample of PWUD attending participating services[2]. In the first stage, we did an exhaustive inventory of all services dedicated to PWUDs. For each participating center, we constructed a comprehensive sampling frame including all opening half-days during the survey period. From this frame, a predefined number of half-day time slots were randomly selected. In the second stage, during each selected time slot, we applied systematic random sampling to invite individuals attending the service to participate. Sampling weights were constructed to reflect both stages of the design.”

3. Were 20% of DBS missing in 2023 because participants refused testing, or because of an invalid test result?

About 20% of DBS samples were not available because participants **did not complete the self-sampling procedure**, rather than due to invalid test results.

In total, 2,125 individuals completed the questionnaire. Among them, 1,736 (82%) agreed to and attempted blood collection using the self-sampling kit. Of these, 1,699 provided DBS samples were both available and had valid test results, representing 80% of the total study sample. We have clarified this point and corrected the corresponding sentence in the **Methods** section of the main manuscript [lines 290–296].

“Biological samples collected using dried blood spots were available for 79%, 92%, and 82% of participants in 2004, 2013, and 2023, respectively. Participants provided self-collected blood samples on blotting paper, which were sent to a centralized virology laboratory. The laboratory performed serological testing for the presence of HIV and HCV antibodies in all three surveys, and molecular testing for HCV RNA in 2013 and 2023 only. Valid laboratory results were obtained for 56%, 85% and 80% of the total participants across the three survey waves, respectively.”

To improve clarity, we also added a flow diagram in the **SI** (**Figure S3**) detailing participant inclusion and reasons for exclusion at each stage of the study for all survey waves.

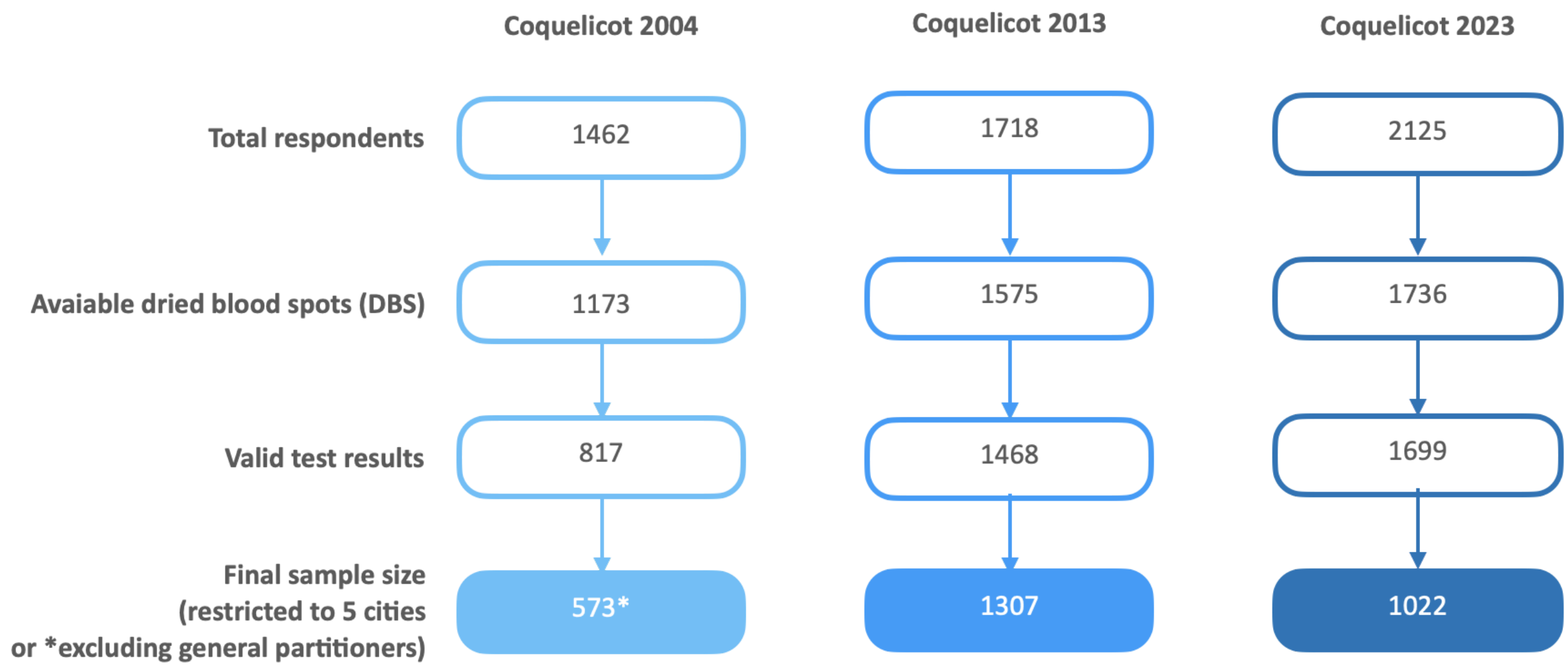

Figure S3. Flowchart of participant inclusion and DBS testing across the Coquelicot surveys (2004, 2013, and 2023). The figure shows the number of total respondents, available DBS samples, valid test results, and final sample sizes (restricted to 5 cities or excluding general partitioners for 2004) for each survey year.

4. How many participants were excluded from the 2013 and 2023 surveys after restricting the analysis to Bordeaux, Lille, Marseille, Paris, and Strasbourg?
In the 2013 survey, the initial sample included 1,468 participants with available and valid test results. After restricting the analysis to Bordeaux, Lille, Marseille, Paris, and Strasbourg, 1,307 participants remained (89%), meaning that 161 participants were excluded.
In the 2023 survey, the initial sample included 1,669 participants with available and valid test results. After applying the same geographic restriction, 1,022 participants (60%) were retained, corresponding to 647 excluded participants.
We added a flow diagram in the **SI** (**Figure S3**) detailing participant selection and exclusions for each survey wave to improve transparency.

5. Suggest using colour-blind friendly palettes in figures, avoiding combining green and red
We modified it according to the reviewer's suggestion.

6. I'm confused about what figure 1. (c) is showing. Is the percentage of participants who were PWID in 2023 around 40%, and the percentage who were non-PWID around 25%? Shouldn't these percentages add to 100%? Can you please clarify
The percentages in **Figure 1c** are calculated using the total number of participants in each survey year as the denominator. Each symbol in the plot therefore represents the proportion of survey samples belonging to a specific combination of city and PWID status.
For example, in 2023, 36.22% (filled circles in **Figure 1c**) corresponds to the share of all participants in that year who were PWID recruited in Paris. Similarly, other values represent the corresponding proportions for each city/ group combination.
Because the denominator is the total sample for each survey year, percentages are not intended to sum to 100% within a city or within a subgroup (PWID and non-PWID). Instead, they

sum to 100% across all cities and both PWID/non-PWID groups for each survey year (see **Table** below).
We clarify this in the figure legend to avoid confusion.

"Percentages represent the distribution of the total sample by city and PWID status within each survey year".

Table. Percentage of injecting drug users (PWID) and non-PWID in five cities (Bordeaux, Lille, Marseille, Paris, and Strasbourg) for the years 2004, 2013 and 2023.

		Bordeaux	Lille	Marseille	Paris	Strasbourg	subtotal
2004	Not PWID	2.19	1.19	1.56	22.13	0.87	27.94
	PWID	4.57	2.19	4.31	54.40	6.59	72.06
2013	Not PWID	2.11	3.54	2.65	24.50	0.91	33.71
	PWID	4.36	3.35	9.73	45.16	3.69	66.29
2023	Not PWID	3.51	5.60	4.56	24.07	0.58	38.32
	PWID	4.96	6.18	13.56	36.22	0.76	61.68

7. The model parameters look reasonable, although should there also be a parameter for the death rate among HCV infected?
To parameterize the model, we used published estimates describing HCV disease progression and adapted them to the compartmental structure considered here. In particular, mortality among HCV-infected PWUDs was modeled as the sum of a baseline mortality rate and an excess mortality attributable to HCV infection, following Refs[2–4]. Specifically, the total mortality rate for HCV-infected individuals was defined as $\mu_2 = \mu_1 + \sigma$, where $\mu_1 = 0.022$ represents the baseline mortality rate among PWUDs, estimated from Refs.[5–7], and $\sigma = 0.03$ denotes the additional mortality associated with HCV infection, based on Refs.[5,8–10].
All parameters are now reported in **Table 2** and **Figure S4**.

8. Can you clarify what the DAA treatment parameter includes? Is it that 23% of participants reported completing DAA treatment in 2023? I looked at reference 12 and couldn't find any information on this
We provide Reference 12 as the source for Coquelicot survey data, as they are not publicly available. The percentages reported in **Table 2** of the main paper (x(t) and z(t)) are derived from self-reported responses in our dataset.
In the questionnaire, participants were asked: *"Have you ever received treatment for hepatitis C?"* Those who answered affirmatively were then asked to specify the most recent treatment they had received. Respondents indicated whether the treatment was interferon/ribavirin, DAAs, or another type of therapy. We subsequently calculated the weighted percentages for the most recent treatments received among HCV infected individuals. Despite relying on self-reported

data, our estimates align with European data[11–13]. Additionally, we performed sensitivity analyses to assess the impact of the fraction of the population on treatments on our results (**Figure S10**) We modified the Methods section as follows [lines 342-344]:

"*Parameters, values, and sources used to define the compartmental models are listed in **Table 2**. In particular, the percentages reported in **Table 2** for the different treatment PWUD received (interferon/ribavirin, direct-acting antivirals) were derived from weighted calculations based on self-reported responses in our dataset."*

(Remarks on code availability) I ran the code and it worked fine and was easy to follow
We thank the reviewer for checking the code.

Reviewer #2 (Remarks to the Author):

The paper considers an important question of whether France has progressed to achieving HCV elimination among PWID through the scale up in HCV treatment and changes in other interventions. The paper uses data from three large surveys, with modelling being used to translate data on prevalence trends into incidence trends. I have the following comments which need addressing:

We would like to thank the reviewer for the careful review of our manuscript. Their comments helped us improve the quality and presentation of our work. We have significantly expanded and deepened the analyses to strengthen the robustness of our findings and better support both aims of the study.

All point-by-point responses are reported here below.

1. In the introduction, when you say you increase accuracy of estimates using HCV RNA-based data, I think you need to say more clearly what you mean. I think the RNA data is used to estimate real decreases in HCV transmission, which HCV Ab prevalence is not so good at doing.

We thank the reviewer for the comment. We revised the introduction section of the main text [lines 70-71] as follows:

"(i) assess anti-HCV prevalence and incidence by age and survey edition, with a focus on recent trends in 2023; and (ii) leverage HCV RNA data to characterize active infections and infer changes in ongoing HCV transmission."

2. What bias is introduced by just recruiting from service settings. What proportion of people do not attend these settings and so would not be represented in your surveys. Does this make your projections of impact optimistic? This should be discussed in a limitations section.

We thank the reviewer for raising this important point. It has now been added to the **limitations** at the end of the **Discussion** section [lines 242-247].

Our recruitment strategy relied on a broad network of specialized services, spanning low- and high-threshold facilities, as well as outreach and street-based teams aimed at reaching highly marginalized PWUDs. These approaches are expected to capture a substantial proportion of the PWUD population and to reflect its diversity. Nevertheless, selection bias may persist, as individuals who are more socially integrated, less vulnerable, or only occasional users may be less likely to attend such services. The exact direction of the resulting bias on HCV prevalence and incidence estimates remains difficult to determine.

However, other studies that consider complementary recruitment strategies, such as respondent-driven sampling (RDS) and a different population, have reported estimates of HCV prevalence comparable to ours (Donnadieu and al., Harm Reduction Journal, 2023). Finally, our Time-Location Sampling strategy ensures a representative sample PWUDs based on randomized selection. This approach yields more robust estimates than non-random, convenience samples (Leon and al., Biostatistics, 2015).

3. It is good to see the comparisons between rounds being limited to the common cities. However, how do you think other changes in scope and eligibility to include crack smoking and including different languages, likely affected your results?

While the core inclusion criteria of the Coquelicot surveys remained broadly consistent across

waves, some modifications were introduced to better capture evolving drug use practices and improve representativeness (Jauffret-Roustide, Int J Drug Policy, 2024). In the 2013 and 2023 surveys, eligibility was expanded to also include individuals reporting crack cocaine smoking only. This modification was introduced in response to the increasing prevalence of crack use in France, which exceeded 30% among participants in the 2004 survey and was not anticipated at the time the survey was launched (Jauffret-Roustide et al., BMC Inf Dis, 2009). It was further supported by growing evidence suggesting that crack smoking practices may contribute to HCV transmission through the indirect sharing of smoking equipment and through their association with other high-risk behaviors (Macias et al., Liver Int ., 2008; Fischer et al.,Eur J Gastroenterol Hepatol., 2008).

Similarly, the expansion of questionnaire languages reflects increasing linguistic diversity within the PWUD population and migration flows. In 2004, the questionnaire was administered only in French, as fewer than 5% of participants reported not speaking French. By 2013, this proportion had increased substantially in some sites (e.g., up to 20% Russian-speaking participants in Paris), leading to the addition of a Russian version. In 2023, further translations were introduced to improve inclusivity and reduce participation barriers among non-French-speaking individuals. **Excluding these groups would likely have introduced selection bias and reduced the relevance of the findings for the contemporary PWUD population in France.**

To assess the potential impact of these adaptations, we compared here estimates from the 2013 and 2023 surveys across four recruitment scenarios:

- French- and non-French-speaking participants, with eligibility including a lifetime history of injecting or snorting drugs or crack smoking (main analysis);
- French-speaking participants only, with eligibility including a lifetime history of injecting or snorting drugs or crack smoking.
- French-speaking participants only, with eligibility restricted to individuals reporting a lifetime history of injecting or snorting drugs (replicating 2004 conditions);
- French- and non-French-speaking participants, with eligibility restricted to individuals reporting a lifetime history of injecting or snorting drugs;

Across all scenarios, the estimates remained consistent with the main findings of the paper (see **Figure** below). Prevalence estimates among French-speaking participants alone were slightly lower in 2023. However, restricting the study population to replicate the 2004 conditions would reduce the representativeness of the contemporary PWUD population which has undergone important demographic and behavioral changes over the past two decades. A dedicated analysis of non-French-speaking and on crack-smokers could be the subject of a future work.

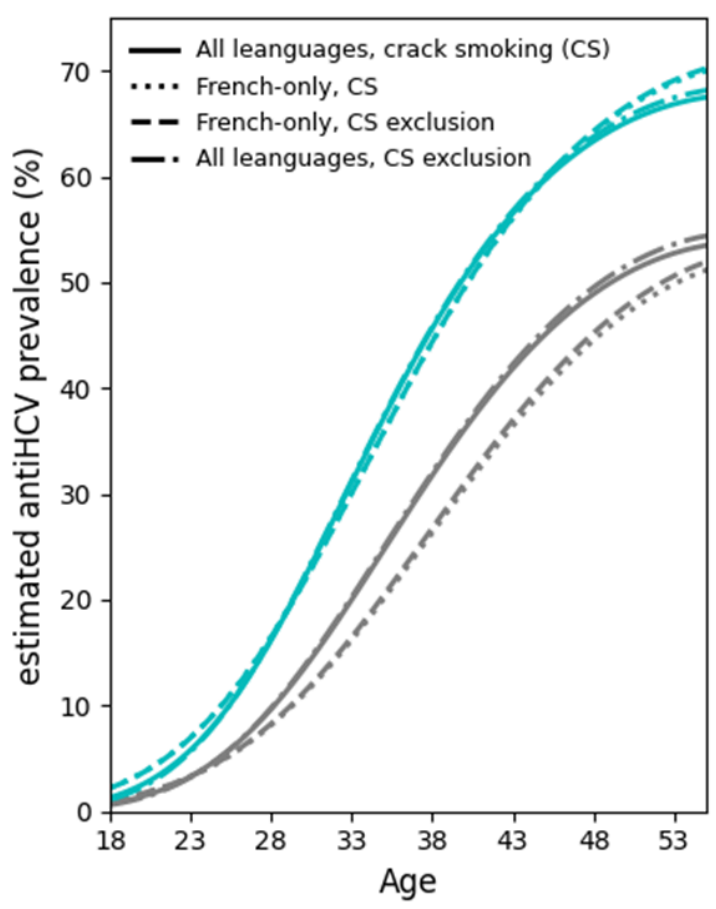

Figure. Impact on inclusion criteria on anti-HCV prevalence among PWUD. Anti-HCV prevalence by age, estimated from the regression model applied to PWUD in 2013 (light blue) and 2023 (grey). Line color indicates survey year. Line type indicates inclusion criteria: French- and non-French-speaking participants; eligibility includes a lifetime history of injecting or snorting drugs or smoking crack (solid line). French-speaking participants only; eligibility includes a lifetime history of injecting or snorting drugs or smoking crack (dotted line). French-speaking participants only; eligibility restricted to a lifetime history of injecting or snorting drugs (dashed line). French- and non-French-speaking participants; eligibility restricted to a lifetime history of injecting or snorting drugs (dash-dotted line).

4. The model for using antibody prevalence data seems ok, although I couldn't see mu2 defined in table 2.

We report here the same answer to **Reviewer #1**:

To parameterize the model, we used published estimates describing HCV disease progression and adapted them to the compartmental structure considered here. In particular, mortality among HCV-infected PWUDs was modeled as the sum of a baseline mortality rate and an excess mortality attributable to HCV infection, following Refs[2–4]. Specifically, the total mortality rate for HCV-infected individuals was defined as $\mu_2 = \mu_1 + \sigma$, where $\mu_1 = 0.022$ represents the baseline mortality rate among PWUDs, estimated from Refs.[5–7], and $\sigma = 0.03$ denotes the additional mortality associated with HCV infection, based on Refs.[5,8–10].
All parameters are now reported in **Table 2** and **Figure S4**.

5. More explanation is needed on how you go from the equations in line 186 to equations on line 189 in the supplementary materials. I can see some of what you may have done but more of the steps need to be included. Also, mu2 does not appear in the equations on line 189. You have also gone from ODE to PDE with no explanation.

We provide here a single reply to all concerns, since they are related to one another. Some of these concerns refer to the choices we made for the structure of the compartmental scheme to model the disease progression, others are the result of an unclear presentation of such a model, which we now have fully revised (**Supplementary Information SI** lines 213-233). The model is formulated as a system of partial differential equations:

*"The HCV transmission model presented in **Figure S4a** is then expressed in the form of partial differential equations by the following system:*

$$\begin{cases} \frac{\partial N(a,t)}{\partial a} + \frac{\partial N(a,t)}{\partial t} = -\lambda(a,t)N(a,t) - \mu_1 N(a,t) + \beta(t)N(a,t) + \epsilon[1-x(t)-z(t)]P(a,t) + \epsilon'[x(t)+z(t)]P(a,t) \\ \frac{\partial P(a,t)}{\partial a} + \frac{\partial P(a,t)}{\partial t} = \lambda(a,t)N(a,t) - (\mu_1+\sigma)P(a,t) - \epsilon[1-x(t)-z(t)]P(a,t) - \epsilon'[x(t)+z(t)]P(a,t) \end{cases} \quad (1)$$

The model is formulated in terms of normalized proportions among the population, such that: N(a,t)+P(a,t)=1, as previously done in literature[8–12]. We can then substitute N(a,t)=1-P(a,t) in the first equation of (1), and express the incidence of HCV infection, λ(a,t), in terms of the derivative of the prevalence function:

$$\frac{\partial(1-P(a,t))}{\partial a} + \frac{\partial(1-P(a,t))}{\partial t} = -\lambda(a,t)(1-P(a,t)) - \mu_1(1-P(a,t)) + \beta(t)(1-P(a,t)) + \epsilon[1-x(t)-z(t)]P(a,t) + \epsilon'[x(t)+z(t)]P(a,t)$$

Then:

$$\lambda(a,t)(1-P(a,t)) = \frac{\partial P(a,t)}{\partial a} + \frac{\partial P(a,t)}{\partial t} - \mu_1(1-P(a,t)) + \beta(t)(1-P(a,t)) + \epsilon[1-x(t)-z(t)]P(a,t) + \epsilon'[x(t)+z(t)]P(a,t)$$

So:

$$\lambda(a,t)(1-P(a,t)) = \frac{\partial P(a,t)}{\partial a} + \frac{\partial P(a,t)}{\partial t} - \mu_1(1-P(a,t)) + \beta(t)(1-P(a,t)) + \epsilon[1-x(t)-z(t)]P(a,t) + \epsilon'[x(t)+z(t)]P(a,t)$$

where $P(a,t) = P(Y=1|a,t)$.

By performing the calculations, we obtain the expression for the incidence as a function of age a at a given fixed time t.

$$\lambda(a|t) = \frac{\frac{\partial P(a|t)}{\partial a} + (\beta-\mu_1) - \left(\beta - \mu_1 - \epsilon\left(1-x(t)-z(t)\right) - \epsilon' x(t) - \epsilon' z(t)\right)P(a|t)}{1-P(a|t)}$$

Thus, with the logit link, the derivative of the prevalence by age a, at a time t*, can be deduced from equation (1) in the* ***Method*** *section of the main paper:*

$$\lambda(a|t) = \frac{\eta'(a)P(a|t)\left(1-P(a|t)\right) + (\beta-\mu_1) - \left(\beta - \mu_1 - \epsilon\left(1-x(t)-z(t)\right) - \epsilon' x(t) - \epsilon' z(t)\right)P(a|t)}{1-P(a|t)}$$

”

As a consequence, μ_2 does not appear explicitly in the final incidence expression because it has been absorbed through the conservation constraint imposed by the normalized population structure. We include these intermediate derivation steps in the revised version to improve clarity. Following Refs[2–4] we defined for completeness $\mu_2 = \mu_1 + \sigma$ where we redefined μ_1 as the mortality rate among PWUDs and σ the mortality rate for HCV-infected PWUDs. Specifically μ_1 =0.022 years^{-1} form refs[5–7] and σ=0.03 form refs[5,8–10]. All parameters are reported in **Table 2** and **Figure S4**.

6. You say similar calculations are used for the HCV RNA model but no details are given. I think this needs to be included to understand what you did because the model will be different in

some important ways. Indeed, I am unsure if the derivative of the prevalence will still be a good representation of the incidence because treatment directly effects the prevalence profile. It is important to see how the authors accounted for the rate of treatment other time.

From the questions raised by the reviewer, we understand that the description of the HCV RNA model was not sufficiently clear.

To parametrize our model we initially considered the $S - I_{ac} - I_{ch}$ compartmental scheme shown in **Figure S5**, consisting of one susceptible compartment (S) and two infectious compartments corresponding to acute (I_{ac}) and chronic (I_{ch}) infection. Individuals in the acute infection compartment may spontaneously clear the virus and return to the susceptible compartment (RNA-), progress to chronic infection, or exit the system due to death. Individuals in the chronic infection compartment may either exit because of death or return to the susceptible compartment following spontaneous viral clearance during the chronic phase or successful treatment.

To parameterize the model, we used available literature describing HCV disease progression (see **Table 2**): for example the model accounts for sustained virological response after treatment, assuming rates of 60% for interferon-based therapies (γ') and 98% for DAAs (γ''), as well as the proportion of individuals reporting having received treatment over time (*x(t), z(t)*). However, because our data do not allow us to distinguish between acute and chronic infection stages and only provide information on RNA positivity versus negativity, we consider a model with a modified-single infectious compartment. **This compartment incorporates all possible pathways through which individuals may return to the susceptible state.** Such simplifications are quite common and several previous HCV modeling studies have adopted similar approaches (Pitcher, J Theor Biol., 2018 - Stone, Plos ONE, 2016).

We have therefore revised the text in the **SI** to provide a more detailed description of the model and mathematical equations to derive incidence [lines 234-264].

As a consequence of our modeling assumptions, the inferred incidence (λ'(a,t)) includes both primary infections and reinfections, the latter occurring among individuals who cleared the infection following sustained virologic response or successful treatment. We acknowledge that this modeling framework assumes that successfully treated individuals re-enter the susceptible compartment with the same risk of infection as before treatment. This simplification may not fully capture the real-world dynamics of DAA treatment programs, where treatment access and post-treatment behavioral changes could modify the risk of reinfection. In practice, DAA treatment is often accompanied by educational initiatives, harm-reduction interventions (e.g., needle and syringe programs or opioid agonist therapy), and behavioral counseling, all of which may reduce the risk of re-infection among treated individuals (Hajarizadeh, J Hepatol, 2022). Conversely, successful treatment may also increase the pool of susceptible individuals and thereby contribute to higher re-infection rates through mechanisms such as risk compensation, reduced re-testing, or population-specific behavioral factors, as found in (Yeung, J. Hepatol, 2022). Because of these complex and potentially opposing effects, many HCV transmission modelling studies assume that the risk of reinfection is the same as primary infection, and therefore do not explicitly incorporate behavioral changes following treatment (Pitcher, J Theor Biol., 2018 - Stone, Plos ONE, 2016 - Martin, Hepatology 2013 - Gountas, Addiction, 2017). We further discuss these points in the **limitations section** of the main manuscript [lines 250–255], as suggested also by **Reviewer #3**.

“Our model also assumes that individuals cured by DAA treatment re-enter the susceptible compartment with unchanged re-infection risk. However, DAA scale-up is often paired with harm reduction strategies (e.g., syringe services, OAT) and education that may lower re-infection rates[45]. Conversely, successful treatment could increase the pool of susceptible individuals, potentially leading to higher re-infection rates if risk behaviors persist or re-testing declines, as suggested in previous studies[33,46]. Future extensions of the model could explore heterogeneous re-infection risks to better reflect empirical observations.”

7. I am surprised antibody prevalence has decreased between the survey rounds. This has not been observed in other settings like England and Scotland. In those settings antibody prevalence has stayed stable but RNA prevalence has decreased considerably. Only in Australia, have they seen large decreases in antibody prevalence since the scale up in treatment and they decided that this was due to more rapid rates of loss of antibody response, with the decrease disappearing when they accounted for people who had lost their Ab response after treatment. See the paper https://pubmed.ncbi.nlm.nih.gov/39159531/ (maher IJDP 2024). Are you sure your decrease in prevalence reflects a decrease in incidence and not something else? Do you have any data to do a similar correction to what they did in Australia? They looked to see what proportion of those with negative antibody response reported ever having been HCV treated.
We thank the reviewer for this insightful comment. To address potential misclassification, we conducted post-hoc adjustment for the 2013 and 2023 survey waves, correcting the data using the method described by Maher et al[14].
More specifically, participants who tested negative on the HCV antibody screening test but reported a history of HCV treatment were reclassified as previously exposed to HCV (anti-HCV+). No data on treatment history were available for the 2004 survey. However, this is unlikely to substantially affect the results, given the very limited access to HCV treatment in France during that period[15], as well as the high burden of adverse effects, which frequently led to treatment discontinuation.

The comparison between the original and corrected estimates is now presented in the **SI** (lines 109-118, **Figure S2**).
All main analyses were performed using the modified prevalence estimates. We have also expanded the corresponding section of the **Methods** to provide a more detailed description of the applied corrections and analytical procedures [lines 297-301].

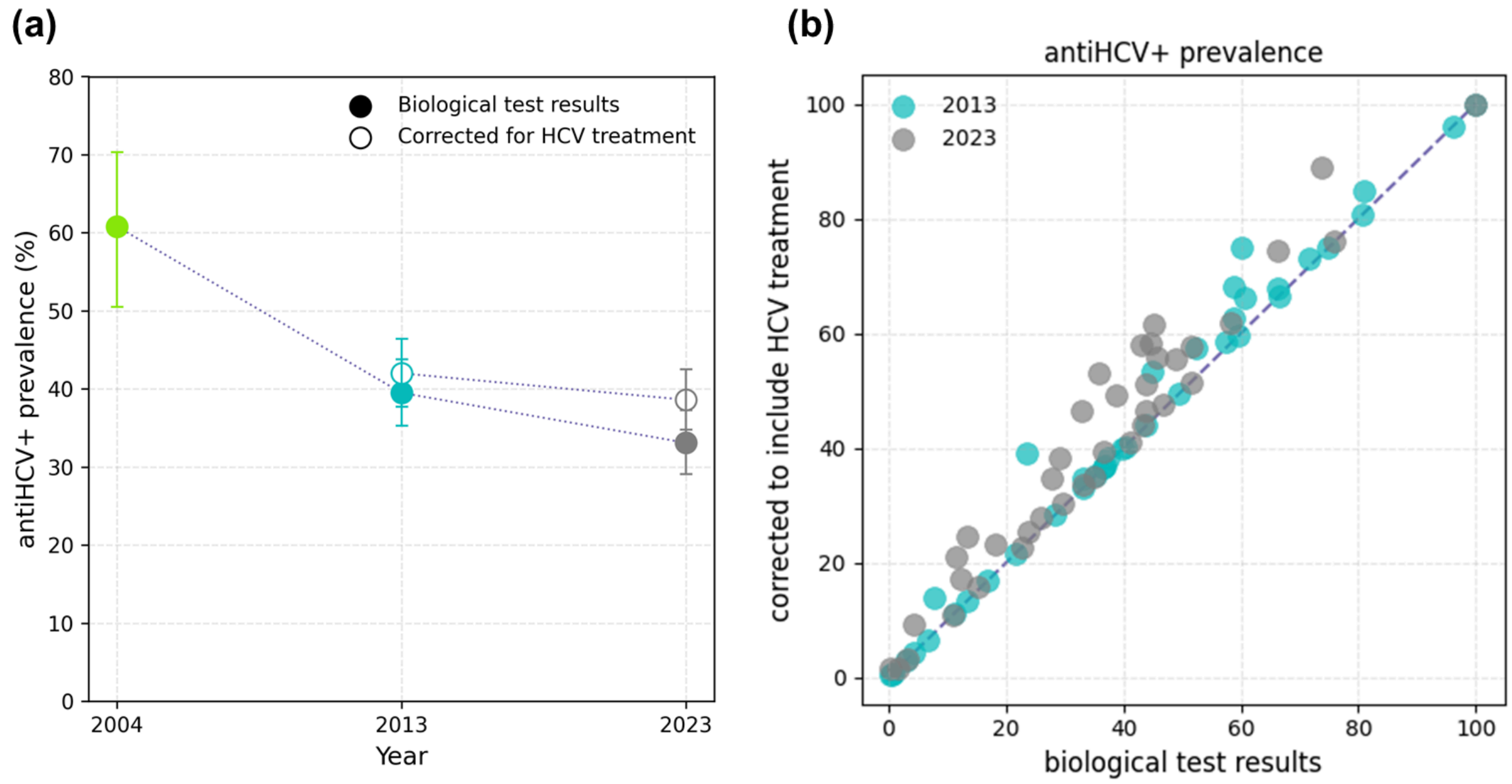

Figure S2. Comparison of anti-HCV+ prevalence data: biological test results vs. corrected for HCV treatment. (a) AntiHCV+ prevalence data for 2004, 2013 and 2023. FIlled circles represent biological test results. Void circles represent corrected data to include HCV treatment. **(b)** Scattered plot between corrected antiHCV+ prevalence data and antiHCV+ prevalence data from biological test results for different ages. Data are shown for 2013 (light blue) or 2023 (grey). Results for these indicators refer to the national scale.

Although the correction slightly attenuated the decline in antibody prevalence, the overall trends and conclusions remained unchanged. The corrected estimates increased in absolute value compared with the original ones. However, unlike Maher et al., we did not observe a relative increase exceeding 50%; instead, we found a more moderate overall median increase of 7.7% in 2013 and 15.2% in 2023.

We agree that the interpretation of declining HCV antibody prevalence deserves further discussion, and we have now expanded and clarified this point in the **Discussion section** [lines 205-216].

First, we would like to emphasize that the decline in HCV antibody prevalence in our data occurred mainly between 2004 and 2013, whereas the difference between 2013 and 2023 was comparatively modest. Antibody prevalence decreased from 60.8% [50.5%, 70.3%] in 2004 to 39.5% [35.3%, 43.8%] (42.0% [37.7%, 46.4%] after correction) in 2013, and to 33.1% [29.1%, 37.3%] (38.6% [34.8%, 42.5%] corrected) in 2023. Furthermore, estimates for 2013 and 2023 appear broadly consistent with observations reported in several other European settings[16]. Unlike France, where harm reduction policies have been progressively expanded, trends in HCV epidemiology among PWUD have been more heterogeneous across countries. For example, some regions in the United States and the United Kingdom have reported stable or even increasing HCV antibody prevalence among PWUD (Bardsley et al., J Viral Hepatitis, 2021).

Second, several additional factors may also contribute to the observed decline in antibody prevalence. Over time, newer generations of people who use drugs may have benefited from increased awareness of HCV risks, broader access to harm reduction interventions, and safer injecting practices, leading to lower cumulative exposure to HCV (**SI** section 5.1, **Figure S6**). At the same time, individuals with longstanding HCV infection who contributed substantially to the

high antibody prevalence observed in 2004 may no longer be represented in later surveys because of aging, mortality, or cessation of drug use.
The Coquelicot surveys are repeated cross-sectional surveys rather than a longitudinal cohort; the observed changes may also reflect shifts in the composition of the sampled PWUD population over time rather than serological changes within the same individuals. We also acknowledge that the study includes only three time points, limiting our ability to precisely determine what may have occurred between survey rounds.
Another possible explanation relates to differences in the policy environment across countries. France has maintained sustained public investment in harm reduction services over the past decades, whereas the availability and funding of such programs have been more variable in other settings. Comparative analyses of harm reduction policies in France and other European countries have highlighted these differences and their potential implications for health outcomes among PWUD (Jauffret-Roustide et al., American Journal of Public Health, 2022).

8. The authors discuss that the difference between antibody and RNA prevalence remained substantial over time. It would be good to see how the prevalence of RNA positivity among people with positive antibody response changes over time. In other settings this percentage has decreased dramatically because of the scale up in treatment.
We appreciate the suggestion of the reviewer. **Table S1** presents the number of individuals meeting these criteria (n=260 in 2013; n=87 in 2023); the absence of 2004 data limits the robustness of temporal comparisons. The weighted prevalence decreased from 43.6% [38.2%, 49.2%] in 2013 to 22.9% [17.9%, 28.8%] in 2023.
Nonetheless, this refers to a rather different research question that deserves a full investigation on its own. For this reason, we feel that it falls beyond the scope of this article.

9. I think the difference between the estimates of incidence from the antibody prevalence data and the RNA prevalence data needs careful consideration. Why do you think it is different?
We thank the reviewer for this comment. Although HCV incidence is a key indicator for monitoring progress toward HCV elimination, it remains challenging to estimate, particularly using repeated cross-sectional data. The gold-standard approach would consist of prospectively identifying incident HCV infections within a longitudinal cohort of at-risk individuals and following them over time. However, such data were not available in our setting.
We therefore explored two approaches based on anti-HCV antibody prevalence and HCV RNA prevalence, respectively. In the antibody-based framework, individuals transition from seronegative (*N*) to seropositive (*P*) status, where *P* includes all anti-HCV-positive individuals regardless of RNA status, i.e. both chronically infected individuals and those who cleared infection spontaneously or following treatment. Consequently, the parameter $\lambda(a, t)$ estimates the force of infection associated with anti-HCV seroconversion, namely the rate at which individuals become ever-infected with HCV.
The discrepancy between incidence estimates derived from antibody prevalence and those derived from RNA prevalence reflects the different stages of infection captured by these markers. Anti-HCV antibodies typically persist following viral clearance, whether spontaneous or treatment-induced, and therefore represent cumulative lifetime exposure to HCV. In contrast, HCV RNA reflects current viraemic infection and is more closely related to ongoing transmission. Consequently, antibody-based estimates may remain relatively high even when active infections are declining, whereas RNA-based estimates are expected to decrease as transmission is reduced and treatment coverage increases. This distinction becomes particularly important following the scale-up of DAA treatment. Successful treatment substantially reduces

the number of viraemic individuals but has little impact on antibody prevalence, as cured individuals generally remain anti-HCV positive. As a result, incidence inferred from antibody prevalence may overestimate contemporary transmission dynamics, while RNA-based estimates are likely to provide a more accurate reflection of ongoing infection risk. This is precisely the phenomenon illustrated in Figures 5a and 5b. For this reason, we consider the RNA-based estimates to provide a more appropriate measure of ongoing HCV transmission in the context of widespread DAA treatment. We have now expanded the **Discussion section** to clarify these points [lines 218–236].

10. Did the modelling account for the changing age distribution of the population in each survey year and what that means for the recruitment rate of new PWID. Similarly, did you control for changes in the population distribution when you assessed the changes in antibody prevalence and RNA prevalence over time.
We thank the reviewer for this question. The age distribution of participants changed across survey waves, reflecting the evolving demographics of the PWUD population over time [lines 22-23 of the abstract, **Table S2 SI**].
Our analyses did not standardize prevalence estimates to a common age distribution. Such standardization would require reliable external information on the underlying age distribution of the target PWUD population, for example from census-based estimates or other representative population-level data. However, no such external reference distribution is currently available for this population. The survey samples themselves therefore represent the best available proxy for the evolving PWUD population structure. The changing age distribution in our samples indirectly reflects shifts in the population. However, we acknowledge that we cannot fully disentangle the effects of demographic changes from other factors influencing prevalence trends. We added a sentence in the main text [lines 215-216].

11. I was also surprised that incidence increases with age – this is contrary to a recent systematic review that found incidence to be highest in young PWID (https://pubmed.ncbi.nlm.nih.gov/36996853/).
We thank the reviewer for raising this important point. In our study, we did not observe a continuous increase in incidence with age; rather, incidence peaked in middle-aged groups and subsequently declined. The discrepancy between our findings and those of the systematic review (Artenie, Lancet Gastroenterol Hepatol., 2023) may be explained by several factors:

- **Context**: the review aggregated global data, which may not accurately reflect the specific epidemiological context of France. Older PWID in France have likely experienced prolonged exposure to HCV risk factors and belong to cohorts in which HCV prevalence was historically high, potentially increasing their likelihood of exposure through long-standing injecting networks. Conversely, younger cohorts have benefited from broader access to sterile injecting equipment and harm reduction interventions. Specifically, the data for France included in the Artenie et al. review are based on an old study (Lucidarme et al.,Epidemiol Infect .,2004), which relied on data from the late 1990s in a single region (northeastern France with specific drug use practices, less injectors compared to other French regions) and small cohorts (fewer than 200 PWUD), potentially failing to reflect more recent trends.
- **Methodological differences:** our incidence estimates were estimated using a mathematical model calibrated specifically to recent French epidemiological data. Consequently, they reflect local assumptions, transmission dynamics, and the current healthcare context which can differ from those underlying global meta-analytic estimates.

Direct comparisons should therefore be interpreted with caution. This variability is further supported by Table S16 in the Supplementary Information of Artenie et al., where Incidence Rate Ratios (IRRs) for HCV acquisition by age group range from negative to positive associations across studies.

As suggested by **Reviewer #3**, we added the following sentence to the **Discussion** section [lines 171–177]:

"The observed age-dependency in incidence likely reflects both the aging and mortality of individuals with HCV antibodies, and a lower rate of new infections among recent PWUD. Younger PWUD are also less exposed to older ones, who have historically acted as reservoirs of transmission. Many young people began injecting at a time when access to sterile equipment and harm reduction services was more widespread. This is particularly true in France, where harm reduction policies were introduced relatively late (in the 1990's[35]) but have since benefited from substantial and consistent investment from the mid-1990s to the present[27]."

It would be good to change figure 2 so that it uses a more standard non logarithmic scale for showing prevalence from 0 to 100% on the y axis. This would help the reader see how the prevalence gradient increases over age up to the ages stated in the text.
We thank the reviewer for the suggestion. We have revised **Figure 2** and **Figure 4** using a linear scale for the y-axis, ranging from 0% to 100%.

Minor comments:
1. Two sentences in a row in introduction second paragraph start with however. I would suggest removing the however at the start of the second sentence
Done.

2. Swop 'ones' for surveys when first mention ANRS-Coquelicot in the introduction
Done.

3. Swop evaluate with evaluating in the last line of introduction
Done.

(Remarks on code availability)
I reviewed the modelling maths which was in insufficient detail.

Reviewer #3 (Remarks to the Author):

In this manuscript the authors apply regression and dynamic compartmental modelling methods to data from three surveys conducted in 5 French cities spaced approximately 10 years apart, to estimate HCV prevalence and incidence over time. The prevalence of HCV positivity (both antibody and RNA) declined over time, and in particular HCV incidence among PWID was estimated to have decreased to 2.4 per 100 person-years, consistent with encouraging progress towards the WHO's 2030 HCV elimination targets. The authors' findings provide a useful addition to the evidence base on the evaluation of progress towards achievement of HCV elimination within the high-risk population of PWUD.
We would like to thank the reviewer for the positive assessment of our manuscript and for the constructive comments. Their feedback helped us substantially improve the presentation of the paper and better highlight the novelty and significance of our findings in the context of existing literature.
A detailed reply to all comments is provided here below.

General comments:

I missed discussion regarding how the authors'results fit within the international context. In any eventual revision, I recommend adding some text comparing/contrasting the current estimates for PWUD in France with evaluation of progress towards the WHO targets reported by other countries. Although an English modelling study is cited (Ward et al., 2025), I believe that the reader would benefit from additional international discussion.
We thank the reviewer for this suggestion. In the revised manuscript, we have expanded the **Discussion section** to place our findings in a broader international context. Specifically, we compare the prevalence and incidence estimates observed among PWUD in France with those reported in other European and high-income countries. We also discuss our findings in relation to progress toward the WHO hepatitis C elimination targets [lines 152–166].

The principal limitations of the modelling techniques are adequately documented. Like similar modelling studies, the modelled estimates of HCV prevalence and incidence do depend on important assumptions and parameter values derived from the data source, some of which may be subject to bias and so could affect the conclusions.
We thank the reviewer for this positive assessment. In the revised manuscript, we have expanded the **Discussion section** and the **Supplementary Information (SI)** to further clarify these assumptions.

Is the effect of DAA treatment on estimated HCV RNA incidence realistically modelled? As infected individuals who are successfully treated simply re-enter the susceptible compartment, the assumption is that there no modification of the incidence rate of re-infection. But in reality, DAA treatment is accompanied with education and harm reduction initiatives that could lead to reduced re-infection rates. In contrast, increased reinfection rates could be observed as a consequence of a growing susceptible pool of PWUD/PWID following successful treatment and/or reduced rates of re-testing, and any increases may be setting/population specific (see e.g. Yeung et al., 2022, J Hep). The authors should discuss this potential limitation further.
We acknowledge that this modeling framework assumes that successfully treated individuals re-enter the susceptible compartment with the same risk of infection as before treatment. We

agree with the reviewer that this simplification may not fully capture the reinfection dynamics; however, such assumptions are quite common in HCV transmission modeling as reinfection risk can either increase (e.g., due to reduced risk perception) or decrease (e.g., due to behavioral changes), making it inherently complex to model. Many studies assume that the risk of reinfection is the same as primary infection, and therefore do not explicitly incorporate behavioral changes following treatment (e.g. Pitcher, J Theor Biol., 2018 - Stone, Plos ONE, 2016 - Martin, Hepatology 2013 - Gountas, Addiction, 2017). Future model extensions could explore time-dependent or subgroup-specific reinfection parameters but this would require robust, context-specific data, as reinfection rates vary across settings[17].
We further discuss these points in the limitations section of the main manuscript [lines 250–255]:

"Our model also assumes that individuals cured by DAA treatment re-enter the susceptible compartment with unchanged re-infection risk. However, DAA scale-up is often paired with harm reduction strategies (e.g., syringe services, OAT) and education that may lower re-infection rates[45]. Conversely, successful treatment could increase the pool of susceptible individuals, potentially leading to higher re-infection rates if risk behaviors persist or re-testing declines, as suggested in previous studies[33,46]. Future extensions of the model could explore heterogeneous re-infection risks to better reflect empirical observations. "

Specific comments:

L96-97: The authors 'note that, regardless of age, antibody prevalence significantly decreases between 2004, 2013, and 2023', and modelled HCV Ab incidence declined over time (consistent with declining prevalence in a study population consisting of different samples each survey round), but the reason for this is not discussed, at least in the main text. In 5.2 of the Suppl ageing and mortality is mentioned. The authors may wish to consider adding a sentence to the main text, rather than locating this only in this Suppl.
We thank the reviewer for this comment, which was also raised by **Reviewer #2**.
We conducted a post-hoc adjustment for the 2013 and 2023 survey waves, correcting the data using the method described by Maher et al[14]. Specifically, participants who tested negative on the HCV antibody screening test but reported a history of HCV treatment, were reclassified as previously exposed to HCV (anti-HCV+).
The comparison between the original and corrected estimates is now presented in the **SI** (lines 109-118, **Figure S2**). Additionally, **all main analyses were repeated using the modified anti-HCV prevalence data**, and we expanded the **Methods** to provide a detailed description of the applied corrections and analytical procedures [lines 297-301].
Although the correction slightly attenuated the decline in antibody prevalence, the overall trends and conclusions remained unchanged. We agree that the interpretation of declining HCV antibody prevalence deserves further discussion, and we have now expanded and clarified this point in the **Discussion section** [lines 205-216]:

"The decline in HCV antibody prevalence observed between 2004, 2013 and 2023 should be interpreted cautiously and likely reflects the combined effect of several epidemiological and methodological factors. Most of the decrease occurred between 2004 and 2013, whereas the difference between 2013 and 2023 was comparatively modest, particularly after correcting prevalence estimates for previously treated individuals who tested anti-HCV negative. The

estimates for the latter survey waves remain broadly consistent with observations reported in other European settings[22]. In addition, generational changes among people who use drugs may have contributed to lower cumulative exposure to HCV over time, through increased awareness of transmission risks, broader access to harm reduction interventions, and safer injecting practices. At the same time, individuals with longstanding HCV infection, who likely contributed substantially to the high antibody prevalence observed in 2004, may no longer be represented in later surveys because of aging, mortality, or cessation of drug use. The Coquelicot surveys are repeated cross-sectional studies rather than a longitudinal cohort; the observed temporal changes may also reflect differences in the composition of the sampled PWUD population across survey waves."

L112-114 The modelled peak incidence at relatively older ages (36-41 years) is interesting, as in contrast to previous studies of PWID populations where acquisition of infection occurs at a much younger age (possibly driven by riskier injection practices). However, in 5.1 of the Suppl the authors note that exposure in their younger-age population could well differ from the older studies regarding access to sterile equipment. As with the preceding comment, adding a sentence to the main text should be considered.
We thank the reviewer for pointing this out. We included in the discussion section the following sentence [lines 171-177]:

"The observed age-dependency in incidence likely reflects both the aging and mortality of individuals with HCV antibodies, and a lower rate of new infections among recent PWUD. Younger PWUD are also less exposed to older ones, who have historically acted as reservoirs of transmission. Many young people began injecting at a time when access to sterile equipment and harm reduction services was more widespread. This is particularly true in France, where harm reduction policies were introduced relatively late (in the 1990's[35]) but have since benefited from substantial and consistent investment from the mid-1990s to the present[27]."

Suppl L191-193: Is there an error in notation? lambda(a,t) is first used to denote incidence of HCV infection, but in line 191 it is also used for prevalence, "we obtain the expression for prevalence for a given time t [as lambda(a,t) = ...]". Please edit if necessary.
Thanks for spotting this. The mistake has been corrected.

Suppl section 4.2: Can you justify why a bootstrapped procedure without replacement was used to determine 95% CIs around outcomes, rather than bootstrapping involving sampling with replacement? The option chosen may lead to underestimation of the width of the 95% CI.
We apologize for the lack of clarity in the original description. The procedure we implemented is effectively a bootstrap with replacement.
Specifically, individuals were replicated according to their survey weights, creating an expanded pseudo-population in which the same original participant could appear multiple times. Bootstrap samples were then generated by randomly reordering this expanded dataset and selecting the first *n* observations for each survey year. Because the expanded pseudo-population contained replicated records, the same original individual could be selected multiple times within a bootstrap sample.
We revised the text in the **SI** to make it clear [lines 266-275]:

"We used a bootstrap approach to assess the variances of the estimates, generating 2000

resampled datasets combining data from the three surveys. First, individuals were replicated proportionally to their survey weights, creating an expanded pseudo-population. For each bootstrap iteration, we assigned a random number to each record (including the copies generated by duplication). Records were then reordered by survey year and by these random numbers. We then selected the first n observations for each survey-year-specific ordered list, with sample sizes of n=573 for 2004, n=1307 for 2013, and n=1022 for 2023. This procedure was repeated 2000 times. For each resample dataset, we calculated prevalence while accounting for the survey design. To estimate incidence, we randomly drew the parameters following the approach of Ref.[4]. Finally, we determined the 95% confidence intervals by extracting the 2.5th and 97.5th percentiles from the distribution of estimated prevalences and incidences across the 2000 generated samples."

Suppl L310-11: The sentence "Results remained robust: assuming 0% or 50% leads to differences in the ballpark of estimations" conveys the opposite of what I believe the author intended. Please edit (is it not correct this analysis suggests no substantial difference in estimated incidence when the spontaneous clearance percentage is varied?).

We agree that the original wording was unclear. We have revised the sentence to clarify that varying the spontaneous clearance percentage produced differences (e.g., the median incidence varied by approximately 30%). However, these differences remained within the confidence intervals of our main analyses.

References

(1) Leon, L.; Jauffret-Roustide, M.; Le Strat, Y. Design-Based Inference in Time-Location Sampling. *Biostat. Oxf. Engl.* **2015**, *16* (3), 565–579. https://doi.org/10.1093/biostatistics/kxu061.

(2) Stocks, T.; Martin, L. J.; Kühlmann-Berenzon, S.; Britton, T. Dynamic Modeling of Hepatitis C Transmission among People Who Inject Drugs. *Epidemics* **2020**, *30*, 100378. https://doi.org/10.1016/j.epidem.2019.100378.

(3) Helikumi, M.; Mushanyu, J.; Mhlanga, A. A Mathematical Model of HCV Transmission Dynamics with Sex Stratification and Environmental Effects. *PLOS ONE* **2025**, *20* (12), e0336374. https://doi.org/10.1371/journal.pone.0336374.

(4) Leon, L.; Kasereka, S.; Barin, F.; Larsen, C.; Weill-Barillet, L.; Pascal, X.; Chevaliez, S.; Pillonel, J.; Jauffret-Roustide, M.; Strat, Y. L. Age- and Time-Dependent Prevalence and Incidence of Hepatitis C Virus Infection in Drug Users in France, 2004–2011: Model-Based Estimation from Two National Cross-Sectional Serosurveys. *Epidemiol. Infect.* **2016**, *145* (5), 895. https://doi.org/10.1017/S0950268816002934.

(5) Meijerink, H.; White, R. A.; Løvlie, A.; de Blasio, B. F.; Dalgard, O.; Amundsen, E. J.; Melum, E.; Kløvstad, H. Modelling the Burden of Hepatitis C Infection among People Who Inject Drugs in Norway, 1973-2030. *BMC Infect. Dis.* **2017**, *17* (1), 541. https://doi.org/10.1186/s12879-017-2631-2.

(6) Mathers, B. M.; Degenhardt, L.; Bucello, C.; Lemon, J.; Wiessing, L.; Hickman, M. Mortality among People Who Inject Drugs: A Systematic Review and Meta-Analysis. *Bull. World Health Organ.* **2013**, *91* (2), 102–123. https://doi.org/10.2471/BLT.12.108282.

(7) Gjersing, L.; Bretteville-Jensen, A. L. Gender Differences in Mortality and Risk Factors in a 13-Year Cohort Study of Street-Recruited Injecting Drug Users. *BMC Public Health* **2014**, *14*, 440. https://doi.org/10.1186/1471-2458-14-440.
(8) Hutchinson, S. J.; Bird, S. M.; Goldberg, D. J. Modeling the Current and Future Disease Burden of Hepatitis C among Injection Drug Users in Scotland. *Hepatol. Baltim. Md* **2005**, *42* (3), 711–723. https://doi.org/10.1002/hep.20836.
(9) Sangiovanni, A.; Prati, G. M.; Fasani, P.; Ronchi, G.; Romeo, R.; Manini, M.; Del Ninno, E.; Morabito, A.; Colombo, M. The Natural History of Compensated Cirrhosis Due to Hepatitis C Virus: A 17-Year Cohort Study of 214 Patients. *Hepatol. Baltim. Md* **2006**, *43* (6), 1303–1310. https://doi.org/10.1002/hep.21176.
(10) Wang, H.-W.; Wang, Y.-C.; Huang, Y.-T.; Jiang, M.-Y. All-Cause and Cause-Specific Mortality Risk among Men and Women with Hepatitis C Virus Infection. *PloS One* **2024**, *19* (9), e0309819. https://doi.org/10.1371/journal.pone.0309819.
(11) Palmateer, N.; Taylor, A.; Goldberg, D.; Munro, A.; Aitken, C.; Samantha, J. Rapid Decline in HCV Incidence among People Who Inject Drugs Associated with National Scale-Up in Coverage of a Combination of Harm Reduction Interventions. *Plos One 9(8)* (e104515).
(12) Rolland, B.; Lions, C.; Di Beo, V.; Carrieri, P.; Authier, N.; Barré, T.; Delorme, J.; Mathurin, P.; Bailly, F.; Protopopescu, C.; Marcellin, F. Adherence to Opioid Agonist Therapy Predicts Uptake of Direct-Acting Antivirals in People Who Use Drugs: Results from the French National Healthcare Database (the ANRS FANTASIO Study). *Harm. Reduct. J.* **2022**, *19* (1), 119. https://doi.org/10.1186/s12954-022-00702-9.
(13) O’Sullivan, M.; Jones, A.-M.; Mourad, A.; Haddadin, Y.; Verma, S. Excellent Hepatitis C Virus Cure Rates despite Increasing Complexity of People Who Use Drugs: Integrated-Test-Stage Treat Study Final Outcomes. *J. Viral Hepat.* **2024**, *31* (2), 66–77. https://doi.org/10.1111/jvh.13897.
(14) Maher, L.; Wand, H.; Heard, S.; Starr, M.; Zolala, F.; Kemp, R.; Cunningham, P.; Drummer, H.; Hellard, M.; Kaldor, J.; Dore, G. Utilising Integrated Bio-Behavioural Surveillance (IBBS) to Investigate Declining Hepatitis C Antibody Prevalence among People Who Inject Drugs in the Australian Needle and Syringe Program Survey. *Int. J. Drug Policy* **2024**, *131*, 104545. https://doi.org/10.1016/j.drugpo.2024.104545.
(15) Direction de la recherche, des études, de l’évaluation et des statistiques (DREES). *La prise en charge des patients atteints d’hépatite C chronique*. https://drees.solidarites-sante.gouv.fr/sites/default/files/2020-10/er470.pdf.
(16) The European Union Drugs Agency (EUDA). *Viral hepatitis elimination barometer among people who inject drugs in Europe*. https://www.euda.europa.eu/publications/data-factsheet/viral-hepatitis-elimination-barometer-among-people-who-inject-drugs-in-europe_en.
(17) Hajarizadeh, B.; Cunningham, E. B.; Valerio, H.; Martinello, M.; Law, M.; Janjua, N. Z.; Midgard, H.; Dalgard, O.; Dillon, J.; Hickman, M.; Bruneau, J.; Dore, G. J.; Grebely, J. Hepatitis C Reinfection after Successful Antiviral Treatment among People Who Inject Drugs: A Meta-Analysis. *J. Hepatol.* **2020**, *72* (4), 643–657. https://doi.org/10.1016/j.jhep.2019.11.012.

Reviewer #1 (Remarks to the Author):

Thanks for your thorough revisions. I am satisfied with the responses.

We sincerely thank the reviewer for their positive and constructive evaluation throughout the review process.

Reviewer #2 (Remarks to the Author):

The authors have replied in detail to all my comments. My only remaining query is their response to comment 10. I agree that it his hard to find a reference population age distribuition, however it would be standard practise when comparing prevalence estimates to adjust for any socio-demographic differences. Because prevalence increases with age it would be important to estimate an adjusted prevalence ratio for each survey round compared to the 2004 survey round that adjusts for differences in age and other factors.

We agree that adjusting for age and other socio-demographic factors is generally preferable to crude prevalence comparisons. However, we remain cautious about applying an age-adjustment without a robust, representative reference population, as this could introduce its own bias rather than resolve it.
New approaches to identify a suitable reference population are currently in our pipeline. We will prioritize this analysis in future work and consider it for a follow-up study.

Reviewer #3 (Remarks to the Author):

I commend the authors on their careful revision of this important manuscript. All of my previous comments have been satisfactorily addressed and I have no further substantive comments.

A very minor point, only noticed in this round : Fig S6 (left panel); the x-axis label is incorrect.

We thank the reviewer for their positive evaluation and for having spotted a typo, which has now been corrected.
